# The rational design of ARUK2007145, a dual inhibitor of the α and γ isoforms of the lipid kinase phosphatidylinositol 5-phosphate 4-kinase (PI5P4K)†

**DOI:** 10.1039/d3md00355h

**Published:** 2023-08-23

**Authors:** Gregory G. Aldred, Timothy P. C. Rooney, Henriette M. G. Willems, Helen K. Boffey, Christopher Green, David Winpenny, John Skidmore, Jonathan H. Clarke, Stephen P. Andrews

**Affiliations:** a The ALBORADA Drug Discovery Institute, University of Cambridge Island Research Building, Cambridge Biomedical Campus, Hills Road Cambridge CB2 0AH UK spa26@cam.ac.uk

## Abstract

The phosphatidylinositol 5-phosphate 4-kinases (PI5P4Ks) are therapeutic targets for diseases such as cancer, neurodegeneration and immunological disorders as they are key components in regulating cell signalling pathways. In an effort to make probe molecules available for further exploring these targets, we have previously reported PI5P4Kα-selective and PI5P4Kγ-selective ligands. Herein we report the rational design of PI5P4Kα/γ dual inhibitors, using knowledge gained during the development of selective inhibitors for these proteins. ARUK2007145 (39) is disclosed as a potent, cell-active probe molecule with ADMET properties amenable to conducting experiments in cells.

## Abstract 2

We report the rational design of PI5P4Kα/γ dual inhibitors. ARUK2007145 (39) is disclosed as a potent, cell-active probe molecule with ADMET properties amenable to conducting experiments in cells.

## Graphical abstract

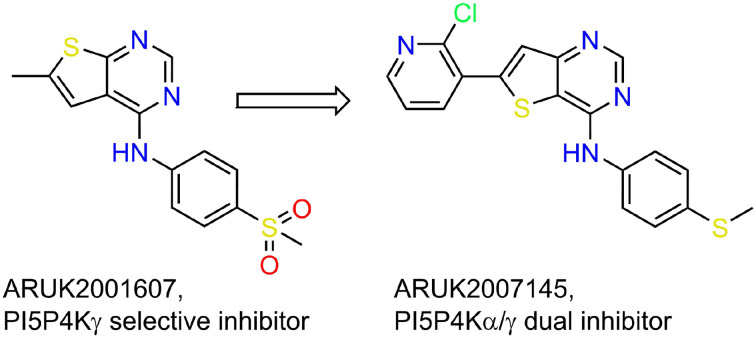

## Introduction

Cellular phospholipids are structural components that delineate the outer boundary of the cell as well as all membrane-bound organelles and compartments. In addition to this structural role these lipids have also been shown to have various other functions, and the phosphoinositides, whilst a minor component of the total cellular phospholipid content, are prolific in their involvement in additional essential cellular processes such as membrane-trafficking, cell proliferation, cytoskeletal organisation, channel regulation and involvement in cell stress and death responses.^1–4^ The canonical route for phosphoinositide signalling results in the production of inositol trisphosphate (InsP3) from phosphatidylinositol (4,5) bisphosphate (PI(4,5)P_2_) as a result of lipase activity, to facilitate cellular calcium release.^5^ Subsequently each of the seven different phosphatidylinositol phosphate species (defined by the phosphorylation state (mono-, bis- or tris-) of positions 3–5 of the 6-carbon ring that constitutes the hydrophilic phosphatidylinositol headgroup^1^) have also been shown to have specific cellular functions, which can be location and cell-type specific.^1,6^ The interconversion of this phosphorylation state is controlled by a group of kinases and phosphatases that may act synergistically to remove or create localised pools of phosphoinositide that can directly (allosteric activation of proteins, regulation of membrane proteins such as ion channels) or indirectly (protein recruitment to membranes *via* specific phosphoinositide-binding or adapter proteins) result in downstream signalling events.^1,2^

Phosphatidylinositol 5-phosphate (PI5P) is converted to PI(4,5)P_2_ by a family of phosphatidylinositol 5-phosphate 4-kinases (PI5P4Ks) consisting of three distinct isoforms; alpha, beta and gamma. Heterogeneity exists within the family; the alpha isoform, *in vitro*, is the most catalytically active,^7^ whereas the gamma isoform has very low intrinsic activity.^8^ The beta isoform is the only one to contain a recognised nuclear localisation sequence.^9^ Although all isoforms are generally ubiquitously expressed, tissue specific differences have been observed.^8^ Diverse cellular functions have also been associated with the different isoforms, for example PI5P4Kβ and PI5P4Kγ may have roles as GTP sensors,^10^ and both PI5P4Kα and PI5P4Kγ have been shown to associate to autophagic structures.^11,12^ Hence the different specific roles reported for each of the isoforms could be functions that are tissue or cell-type specific, or spatially or temporally differentiated in response to activation of different metabolic pathways. Structurally there are also differences between the isoforms^9,13^ but it is clear that each is able to heterodimerise,^7,8^ which leads to the intriguing possibility that isoforms are able to regulate the localisation, specific function or *in vivo* activity of each other.

The promiscuity of the phosphoinositides in cellular signalling pathways has implications for disease as enzyme dysfunction or misregulation may be causative in various developmental disorders, inflammation and infection.^13–16^ As oncology targets, PI5P4Ks have become increasingly associated with a number of diseases.^17^ Interestingly the involvement of PI5P4Kα alone has been documented in glioblastoma, acute myeloid leukemia (AML) and prostate cancer^18–21^ but other examples suggest that there may be a combinatorial role for the PI5P4Ks. Both PI5P4Kα and PI5P4Kβ are involved in p53-deficient breast cancer and soft tissue sarcomas^22,23^ and high expression of both PI5P4Kα and PI5P4Kγ associate with unfavourable clinical outcome in AML.^24^

Several recent reports detail inhibitors for PI5P4Ks, not only pan-specific^25–29^ but also isoform specific.^30–34^ Emerging tools are also being developed for dual-specific inhibitors for PI5P4Kα and PI5P4Kβ^22,35^ and methods have also been reported for removing protein completely in cells using PROTAC systems, such as JWZ-1-80 for PI5P4Kγ,^36^ which will be useful to interrogate non-catalytic roles. Here we present a new tool that can be used to investigate the impact of dual inhibition of both the PI5P4Kα and PI5P4Kγ isoforms.

We have previously reported PI5P4Kγ-selective inhibitors including ARUK2001607 (1) which was derived from virtual screening hit 2,^30^ as well as PI5P4Kα-selective inhibitors including tool compound ARUK2002821 (3) which was derived from virtual screening hit 4 (ref. 33) (Table 1). During the course of those studies, we also became interested in developing dual PI5P4Kα–PI5P4Kγ inhibitors as probe molecules. In particular, we had observed small amounts of PI5P4Kα inhibition in some analogues of 1 which we were able to further optimise through rational design. Herein we describe efforts to further increase the level of PI5P4Kα activity in the 1 chemotype towards the development PI5P4Kα/γ dual inhibitors with IC_50_ fold-selectivities within approximately 10-fold in either direction.

**Table 1:** Previously reported PI5P4K ligands 1–5

Compound		Inhibition of PI5P4K			Physicochemical properties	
		PI5P4Kα pIC_50_	PI5P4Kγ+ pIC_50_	Fold-selectivity (α IC_50_/γ IC_50_)	*M*_W_	*X* log *P*
1	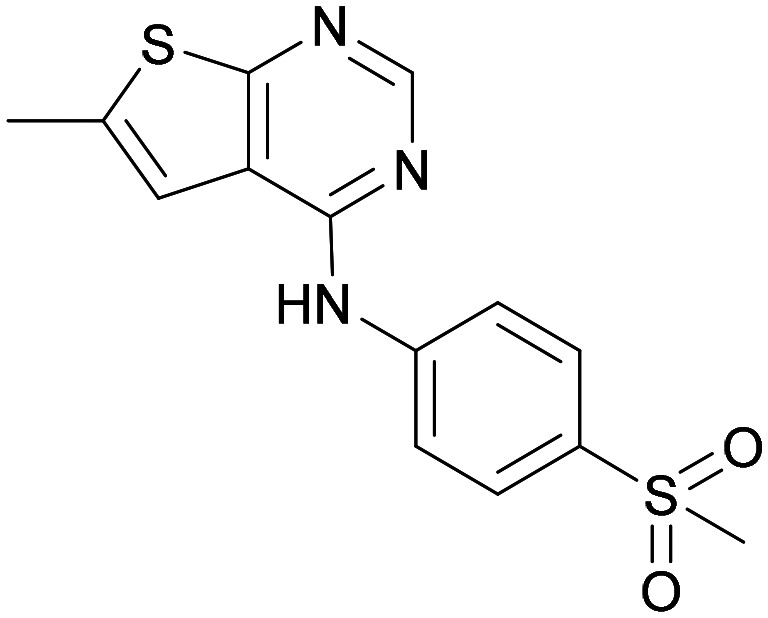	<4.8	7.1	>210	319	2.8
2	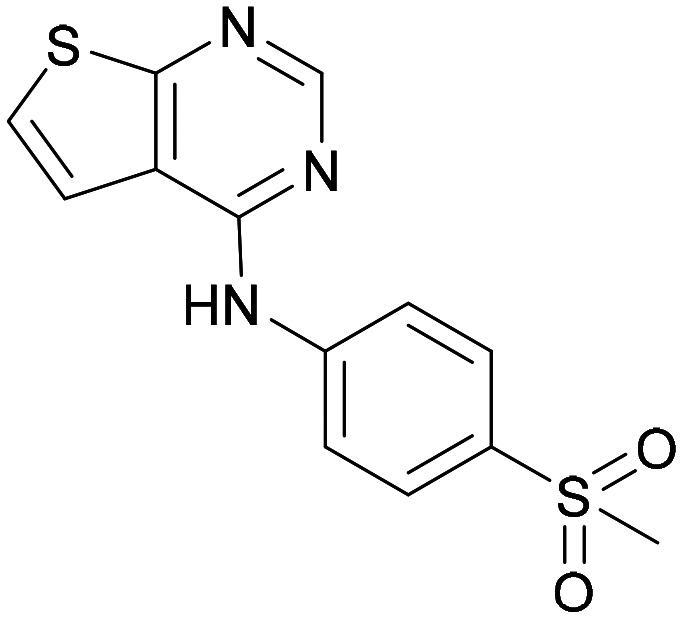	<4.3	6.5	>170	305	2.6
3	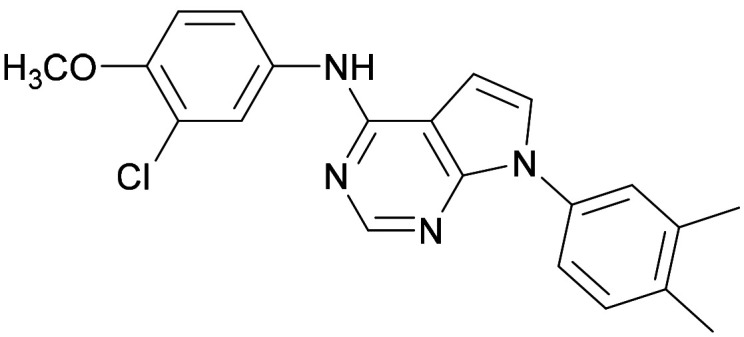	8.0	<4.3	<0.0002	379	5.8
4	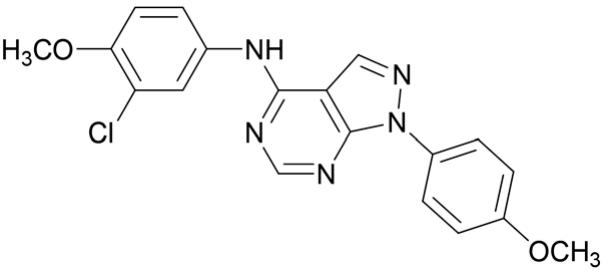	6.4	4.9	0.032	382	4.2
5	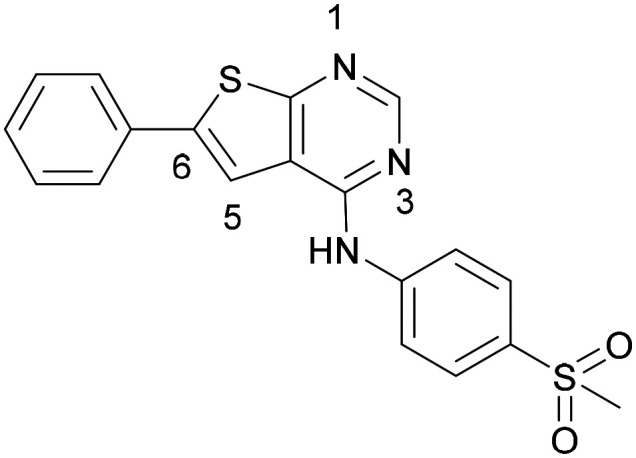	<5.3	7.4	>130	381	4.5

## Results

Compound 5 was a lead in the development of our novel dual PI5P4K inhibitors. Compared to 1 and many of the other analogues previously explored in this chemical series,^30,37^5 was differentiated by having a larger substituent at the 6-position of the thienylpyrimidine core (Table 1). This group gave a point of diversity for further SAR exploration and this compound appeared to be on the threshold of detection in the PI5P4Kα ADP-Glo assay, sometimes giving a partial inhibition curve leading to an inconsistent pIC_50_ with hints that the vector at the 6-position of the thienylpyrimidine core may be of value introducing further PI5P4Kα activity.

**Fig. 1:**
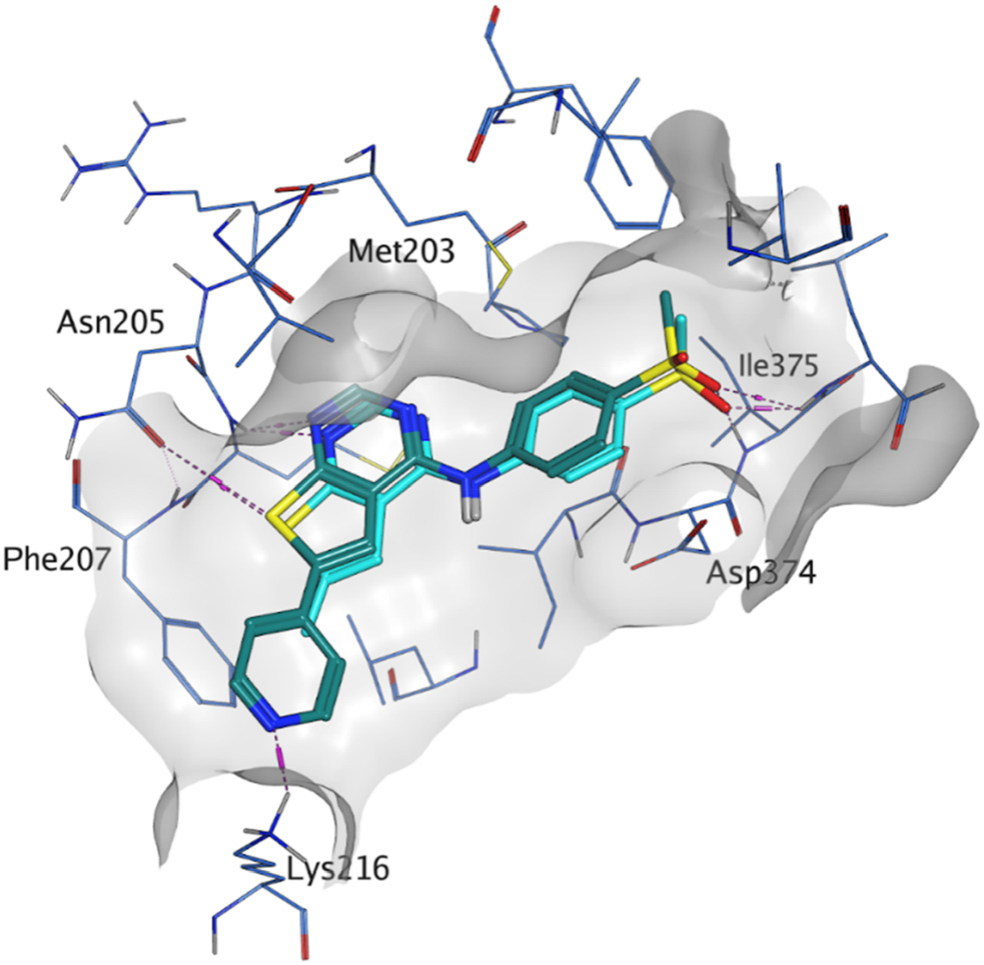
Compound 6 (green) docked into the 8BQ4 structure of PI5P4Kγ–1 (cyan).

As the phenyl group of 5 had an increased *M*_W_ and *X* log *P* compared to 1 we also focussed on improving physicochemical properties during iterations of compound design. In particular, we were keen to introduce heteroatoms in this ring to modulate *X* log *P*. The first synthesised compound of this type, 6, shows a marked decrease in *X* log *P* compared to 5 and was found to have increased potency for PI5P4Kα and PI5P4Kγ+ (Table 2).^37^ When the 8BQ4 crystal structure of PI5P4Kγ was later solved with 1 bound in the ATP binding site, docking of 6 suggested that its pyridyl N might interact with Lys216 of PI5P4Kγ (Fig. 1).

**Table 2:** Variation of *R*^1^ to introduce heteroaryl groups

	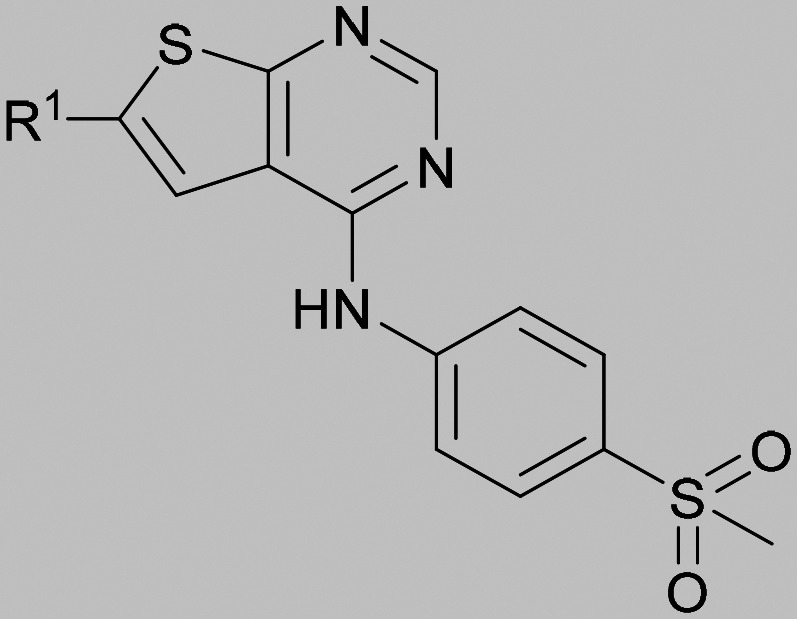	Inhibition of PI5P4K			Physicochemical properties	
	*R* ^1^	PI5P4Kα pIC_50_	PI5P4Kγ+ pIC_50_	Fold-selectivity (α IC_50_/γ IC_50_)	*M*_W_	*X* log *P*
5	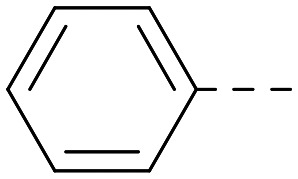	<5.3	7.4	>130	381	4.5
6	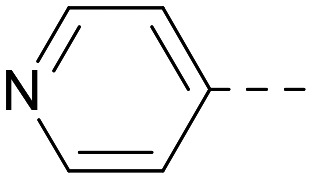	5.9	7.7	63	382	3.1
7	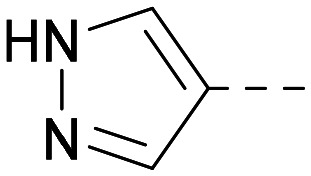	5.9	7.8	79	371	3.7
8	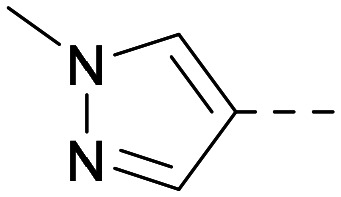	5.2	7.4	160	385	3.1
9	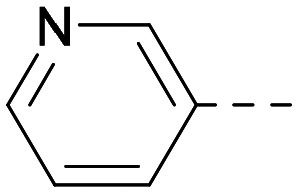	5.3	7.5	130	382	3.1
10	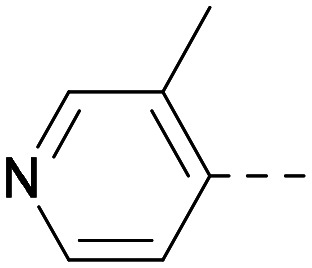	6.2	7.9	50	396	3.4
11	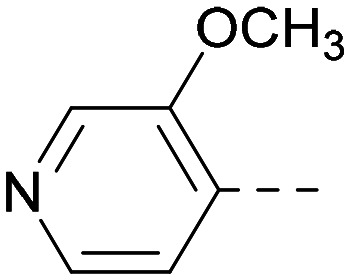	5.6^a^	7.5^a^	79	412	3.1
12	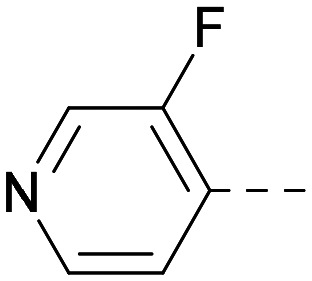	<4.6	7.0	>250	400	3.2
13	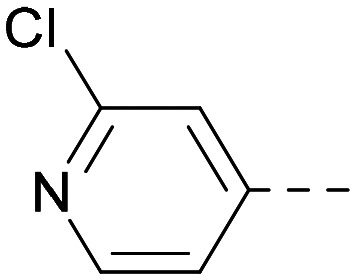	5.1^a^	7.2	130	417	4.2
14	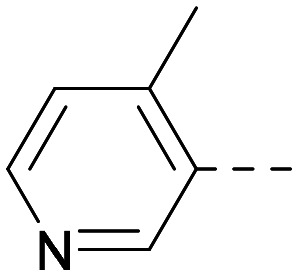	6.0	7.9	79	396	3.4
15	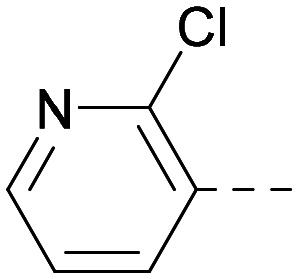	6.2	7.9	50	417	4.2
16	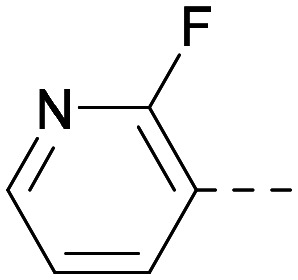	5.8^a^	7.5	50	400	3.7
17	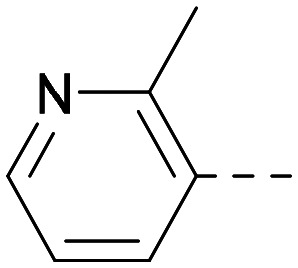	6.0	7.7	50	396	3.3

a Inconsistent pIC_50_ measurements between replicates; value shown is upper limit.

Indeed, a range of heterocycles was tolerated at the 6-position of the thienylpyrimidine, with PI5P4Kγ+ activity being retained when compared to 5 and many showed some level of PI5P4Kα inhibition as well as a reduction in *X* log *P* (Table 2). Pyrazoles 7 and 8 retained good levels of PI5P4Kγ+ inhibition whilst showing some PI5P4Kα inhibition (Table 2). Pyridine 9 showed higher selectivity towards PI5P4Kγ+ than 6 (160 *vs.* 63-fold, respectively). The relative PI5P4Kα activity of 6 could be increased by methylation of the pyridyl 3-position (10) more effectively than by addition of OMe (11), or F (12) at the same position, or by chlorination at the pyridyl 2-position (13; Table 2). A range of small substituents was also trialled on template 6 and these were generally well tolerated (see 14–17). Compound 17 was a useful lead from this set with a good balance of activity at PI5P4Kα and γ+ as well as one of the lower *X* log *P*s in this series. Compound 17 was therefore selected as a template for further exploration around the sulfone group.

**Fig. 2:**
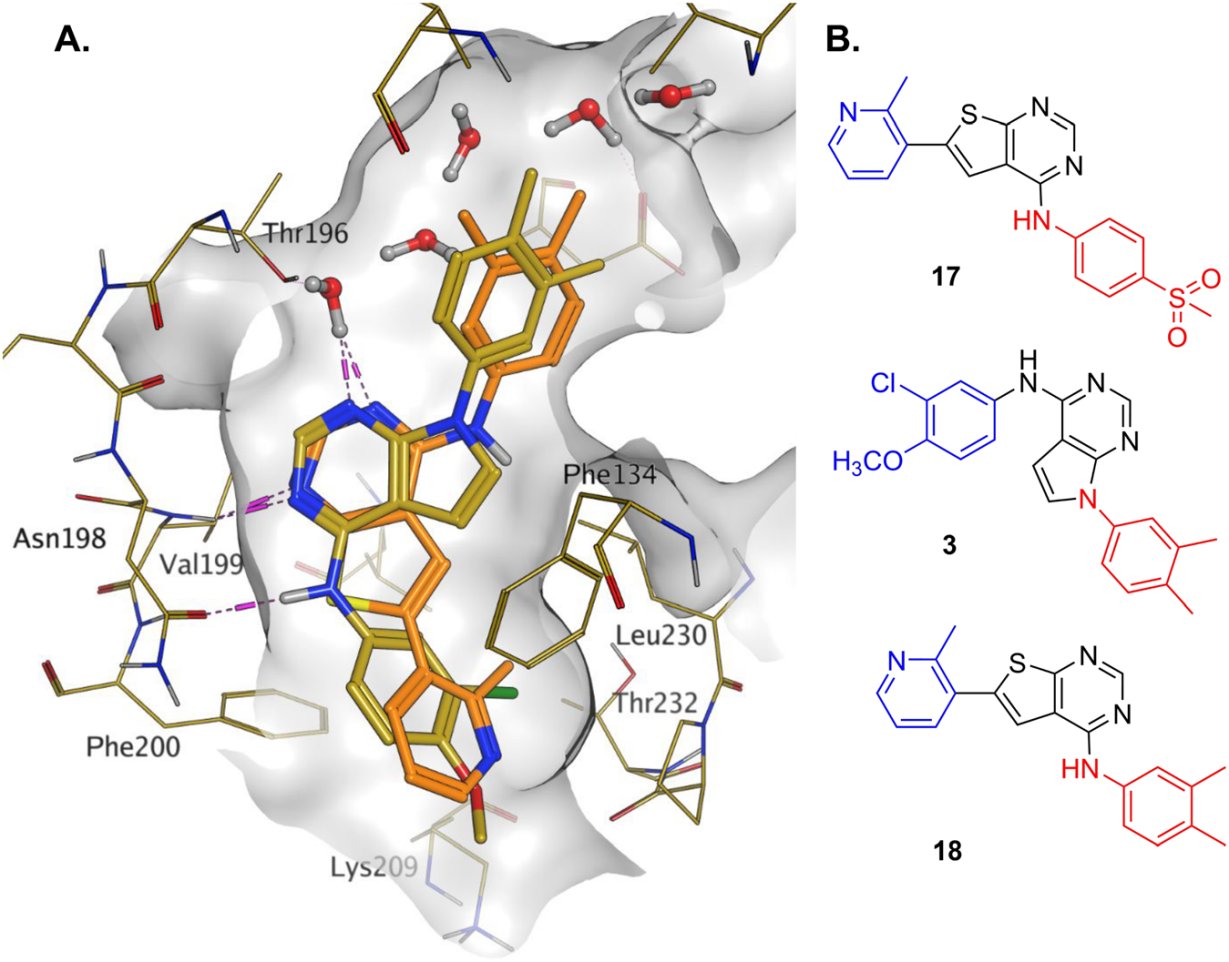
A) Compound 18 (orange) docked into the 8C8C structure of PI5P4Kα–3 (gold). The dimethylphenyl group of 3 overlays well with that of 18; B) 2D comparison of 3, 17 and 18.

Fig. 1 shows that the arylsulfone of dual inhibitor 6 is predicted to overlay well with the corresponding group of the PI5P4Kγ-selective molecule 1 in the PI5P4Kγ structure (8BQ4). A question remained as to whether the corresponding group of PI5P4Kα-selective 3 (*i.e.* 3,4-dimethylphenyl) would transpose onto this thienylpyrimidine template (Fig. 2). Using 17 as the optimal compound from Table 2, the dimethylphenyl group of 3 was transposed to provide compound 18, which was docked into the 3 structure (8C8C, Fig. 2).

The rationally-designed compound 18 was found to have improved PI5P4Kα activity compared to 17 but diminished PI5P4Kγ+ activity (Table 3). An alternative potent PI5P4Kα inhibitor with a 2,4-dimethylphenyl group, “compound 25”,^33^ was also merged onto the thienylpyrimidine core to give the resulting compound 19. Both 18 and 19 are dual PI5P4Kα/γ+ inhibitors, as predicted. Indeed, the designed modifications increased the PI5P4Kα activity of this chemotype to the point that, relative to PI5P4Kγ-selective starting point 1, both 18 and 19 showed higher levels of PI5P4Kα than PI5P4Kγ+ inhibition (for reference, compound 1 showed no detectable inhibition of PI5P4Kα under the same assay conditions, Table 1).

**Table 3:** Exploration of *R*^2^

	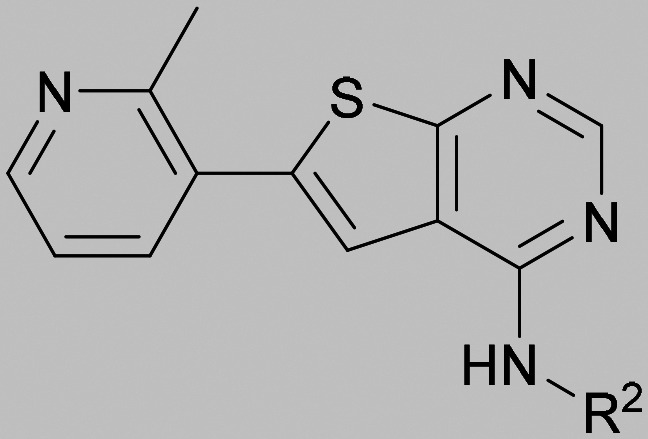	Inhibition of PI5P4K			Physicochemical properties	
		PI5P4Kα pIC_50_	PI5P4Kγ+ pIC_50_	Fold-selectivity (α IC_50_/γ IC_50_)	*M*_W_	*X* log *P*
17	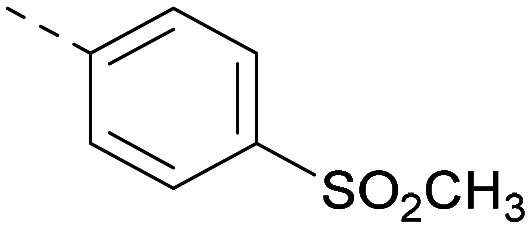	6.0	7.7	50	396	3.3
18	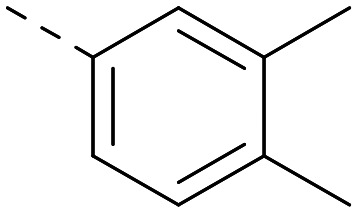	6.5	5.9	0.25	346	4.7
19	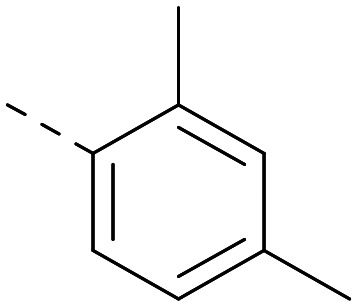	5.5	5.4	0.79	346	4.7
20	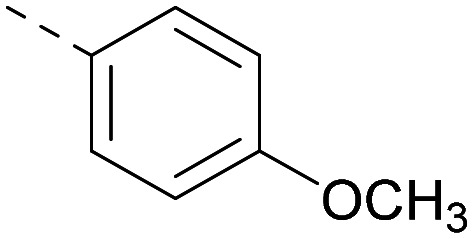	5.9	5.7	0.69	348	4.1
21	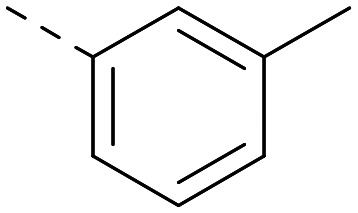	5.5	5.3	0.63	332	4.4
22	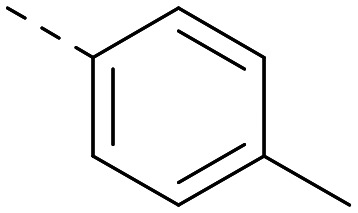	6.2	5.8	0.4	332	4.4
23	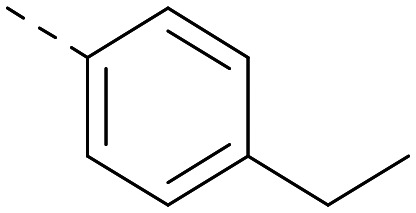	6.2	6.4	1.6	346	4.8
24	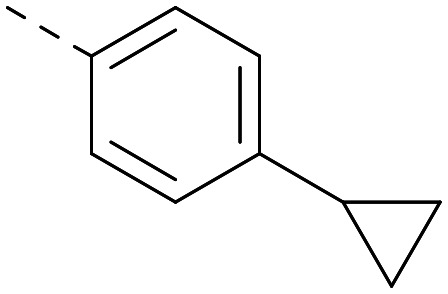	6.6	7.0	2.5	358	4.9
25	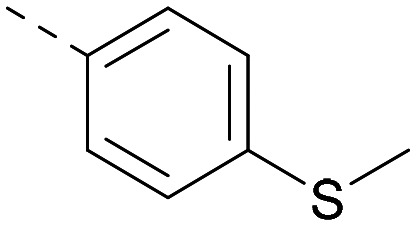	6.6	7.3	5.0	364	4.8

Lipophilic substituents at *R*^2^ have previously been found to be important for PI5P4Kα inhibition.^33^ As such, a variety of other lipophilic groups was tested at this position (Table 3). Compounds 20 and 21 were modest dual inhibitors and an interesting SAR trend was observed for the series of small aliphatic groups for 22–24, which increased in both PI5P4Kα and PI5P4Kγ+ activity with increasing size of the *para* substituent. In agreement with the hypothesis that the polar sulfone of 17 was not favourable for PI5P4Kα inhibition, apolar sulphide 25 was synthesised. This molecule showed an expected increase in PI5P4Kα inhibition with a modest reduction in PI5P4Kγ+ inhibition to afford a potent dual inhibitor.

Following on from this, combinations of groups which had been productive at *R*^1^ and *R*^2^ were explored (Table 4). Owing to the preference for lipophilic groups at *R*^2^, it was desirable to use polar heterocyclic groups at position *R*^1^, where possible, to help offset *X* log *P*. Nonetheless, these compounds were generally high in *X* log *P*, typically in the range 4–5 (compounds 26–30).

**Table 4:** Combinations of *R*^1^ and *R*^2^ groups which, individually, had given advantages in Tables 2 and 3

	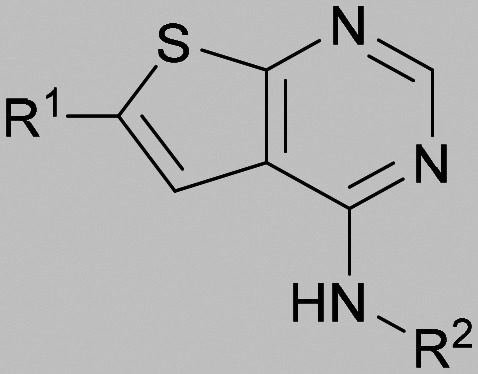		Inhibition of PI5P4K			Physicochemical properties	
	*R* ^1^	*R* ^2^	PI5P4Kα pIC_50_	PI5P4Kγ+ pIC_50_	Fold-selectivity (α IC_50_/γ IC_50_)	*M*_W_	*X* log *P*
20	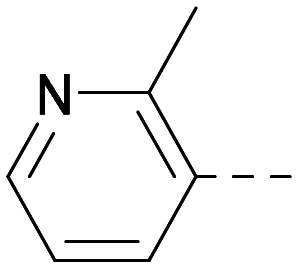	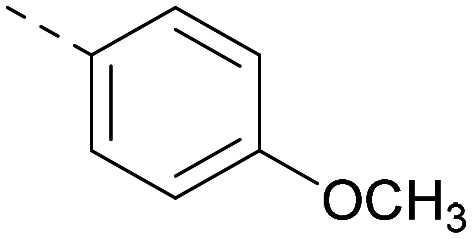	5.9	5.7	0.69	348	4.1
26	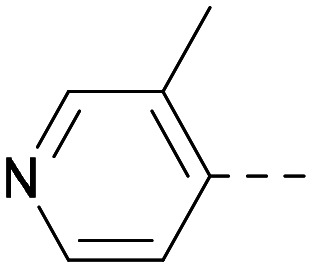	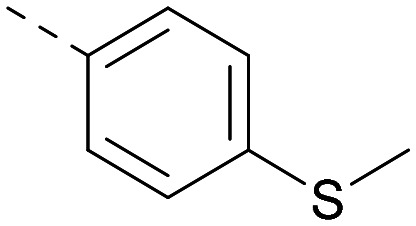	6.9	7.7	6.9	364	4.8
27	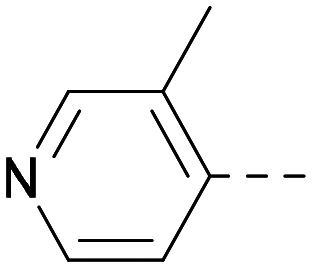	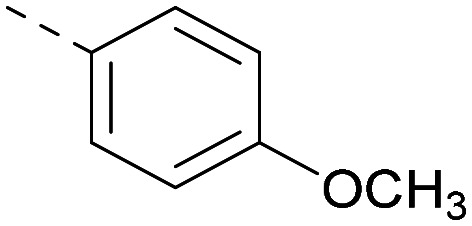	6.2	6.2	1.1	348	4.2
28	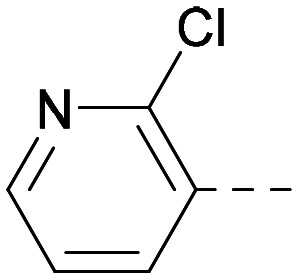	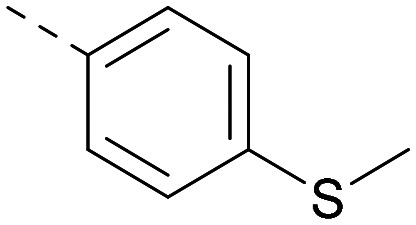	6.6	7.7	12	385	5.6
29	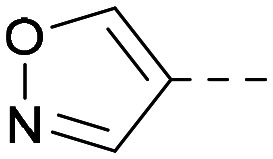	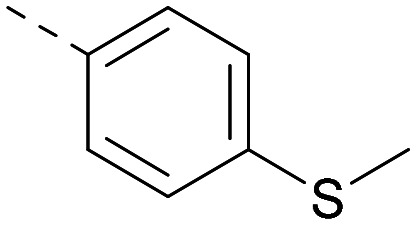	6.6	7.4	6.0	340	4.0
30	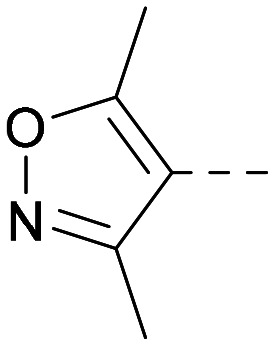	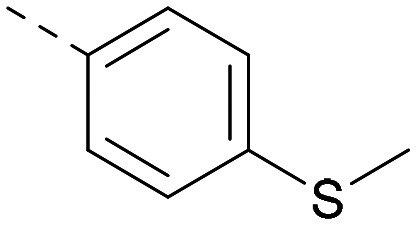	6.8	7.6	6.3	368	4.4

Substitution at the 5-position of the thienopyrimidine core was also investigated (Table 5). In general, this position was tolerant of a wide range of substituents from small lipophilic groups such as methyl (31) and chloro (32), to larger substituents such as a small ring (33) or a much larger group incorporating a polar amide moiety (34). A 5,6-disubstituted thiophene was also tolerated (35). However, these derivatives did not offer an advantage over 6-substituted thienylpyrimidines and were not pursued further.

**Table 5:** 5-Substituted thieno[2,3-*d*]pyrimidine analogues

	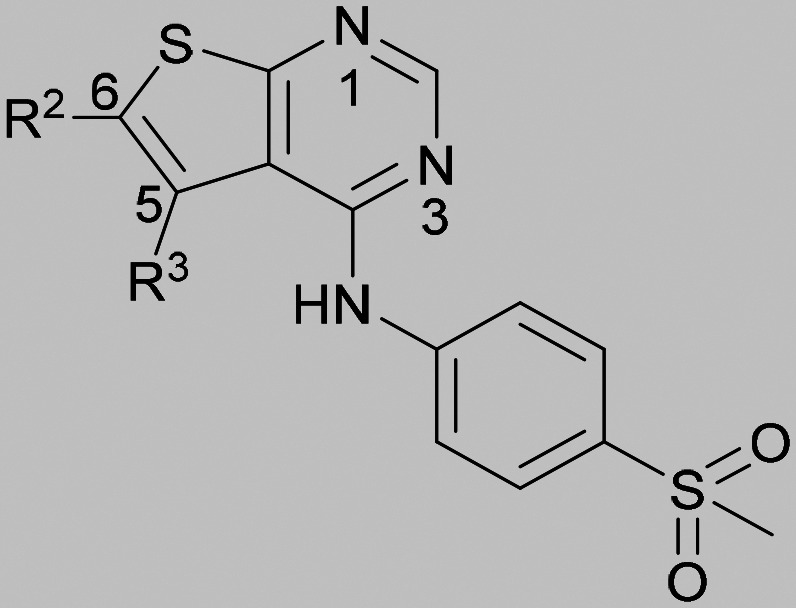		Inhibition of PI5P4K			Physicochemical properties	
	*R* ^2^	*R* ^3^	PI5P4Kα pIC_50_	PI5P4Kγ+ pIC_50_	Fold-selectivity (α IC_50_/γ IC_50_)	*M*_W_	*X* log *P*
9	3-Pyridyl	H	5.3	7.5	130	382	3.1
31	H	CH_3_	<4.3	7.3	>980	319	2.9
32	H	Cl	<6.2	7.3	11	340	3.2
33	H	*c*-Pr	<4.8	7.0	140	345	3.4
34	H	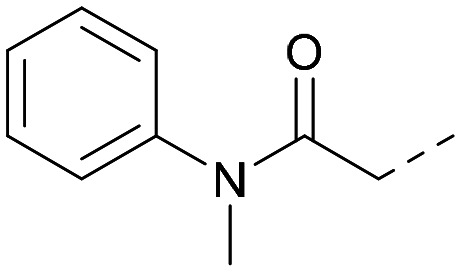	5.6	7.3	50	439	4.2
35	CH_3_	CH_3_	<4.3	6.7	>250	333	3.1

The thiophene was also ‘reversed’ from thieno[2,3-*d*]pyrimidines to afford thieno[3,2-*d*]pyrimidine derivatives such as 36, which is a direct analogue of 2 but with the sulfur atom moved within the thiophene ring (Table 6). Compound 36 shows a higher PI5P4Kγ+ pIC_50_ than 2 (7.3 *vs.* 6.5, respectively) and both compounds had undetectable levels of PI5P4Kα inhibition. Matched pairs of molecules in which the regiochemistry of the thienopyridine was alternated while the R groups were kept constant showed that thieno[3,2-*d*]pyrimidine was generally the more active regioisomer. For example, compound 38 forms a matched pair with 24 with the former being slightly more active at both isoforms (PI5P4Kα 7.0 *vs.* 6.6, respectively, and PI5P4Kγ+ 7.3 *vs.* 7.0). Similarly, 39 forms a matched pair with 28, with 39 being slightly more active at both PI5P4K isoforms. The thieno[3,2-*d*]pyrimidine template was also used to explore further alternative lipophilic groups at *R*^2^ (40, 41) and a group from potent PI5P4Kγ inhibitor ‘compound 7’ (42).^30^

**Table 6:** Thieno[3,2-*d*]pyrimidine analogues

	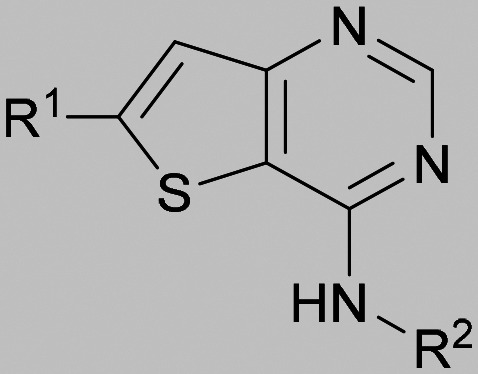		Inhibition of PI5P4K			Physicochemical properties	
	*R* ^1^	*R* ^2^	PI5P4Kα pIC_50_	PI5P4Kγ+ pIC_50_	Fold-selectivity (α IC_50_/γ IC_50_)	*M*_W_	*X* log *P*
36	H- -	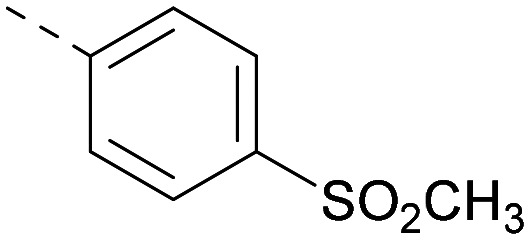	<4.3	7.3	1000	305	2.6
37	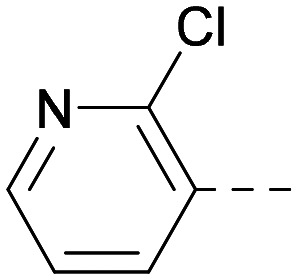	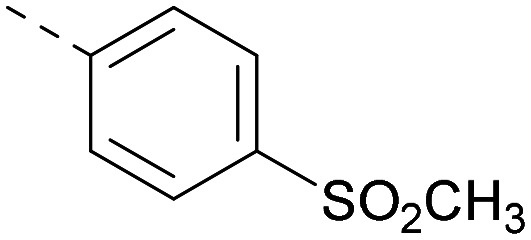	6.5	7.9	5.4	417	4.2
38	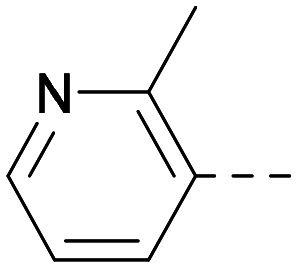	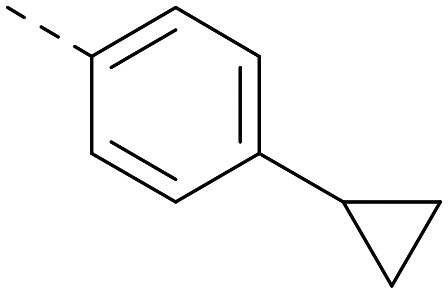	7.0	7.3	1.9	358	4.9
ARUK2007145 (39)	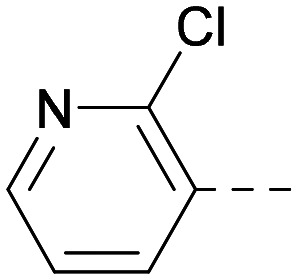	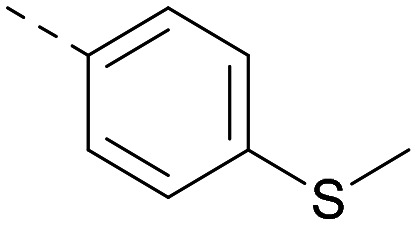	7.3	8.1	5	385	5.6
40	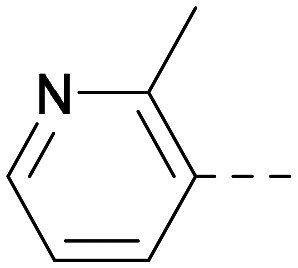	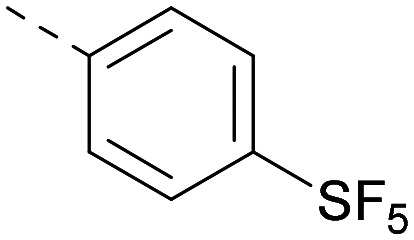	6.2	7.0	6.3	444	6.2
41	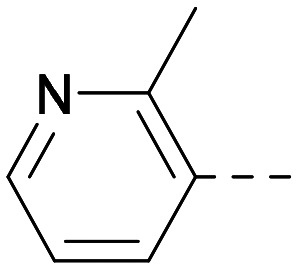	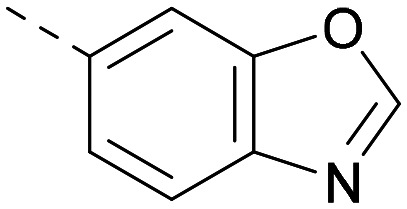	5.5	6.3	5.9	359	4.6
42	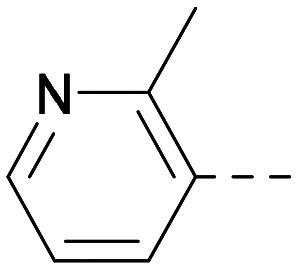	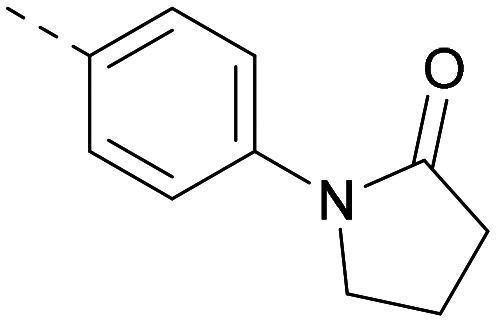	7.0	8.1	13	401	4.4

A selection of the more active compounds identified to this point was profiled for PI5P4Kβ activity as well as PI5P4Kγ WT activity and cell penetration in a PI5P4K-WT InCELL Pulse cellular target engagement assay (Table 7). Both assays have previously been described for screening PI5P4Kγ inhibitors.^38^ All compounds tested in the PI5P4Kβ ADP-Glo assay returned IC_50_s > 10 μM (Table 7). Furthermore, there is a good correlation between the pIC_50_s obtained in the PI5P4Kγ+ ADP-Glo assay and the PI5P4K-WT InCELL Pulse assay, confirming that these compounds are *bona fide* inhibitors of the wild-type form of PI5P4Kγ. In general, there is a slight drop in pIC_50_ in the cellular assay, possibly owing to the requirement for cell permeability and/or the non-specific binding of the compounds to cellular components; however, these compounds such as 10 and 39 are potent PI5P4Kγ inhibitors in live cells.

**Table 7:** PI5P4Kα, β and γ inhibition (ADP-Glo) and PI5P4Kγ cellular target engagement (InCELL Pulse) for selected compounds

	ADP-Glo pIC_50_			InCELL Pulse pIC_50_
	PI5P4Kα	PI5P4Kβ	PI5P4Kγ+	PI5P4Kγ-WT
5	<5.3	<4.6	7.4	6.6
8	5.2	4.9	7.4	6.7
10	6.2	4.9	7.9	7.7
15	6.2	4.9	7.9	7.1
17	6.0	4.7	7.7	7.3
37	6.5	ND	7.9	7.4
39	7.3	ND	8.1	7.3

Compounds with interesting profiles were selected for screening in further assays to assess ADMET properties with the aim of identifying potent dual inhibitors that would make suitable probes for applications in cellular experiments (Table 8). As such, cell permeability and efflux were measured in MDCK-MDR1 cells and aqueous solubility was measured at pH 7.4. To give an indication as to whether any of these probes may be suitable for *in vivo* testing, stability in mouse liver microsomes (MLMs) was also measured. In general, there was a wide range of values in each of the ADMET assays for this series of molecules. Efflux ratios varied from good (0.91) to very high (64). Cell permeabilities were generally good, ranging from low at 1.3 × 10^−6^ cm s^−1^ to high at 14.2 × 10^−6^ cm s^−1^. Solubilities ranged from low to moderate (1–30 μM). Half-lives in MLMs ranged from very poor to very good (0.94–383 minutes).

**Table 8:** ADMET data for selected compounds

	Efflux ratio^a^	*P*_app_ a (A2B, 10^−6^ cm s^−1^)	Solubility^b^ (μM)	MLM *t*^½^^c^ (min)
5	1.3	6.23	1	54.5
8	26.4	2.88	30	73.3
10	25.2	3.13	30	9.23
14	34.1	2.45	30	8.2
15	15.7	4.73	3	383
17	64.3	1.33	10	35.5
26	1.1^d^	3.7^d^	3	0.94
32	3.7	14.2	10	51.4
37	31.8	1.33	10	70.8
38	0.91	9.95	3	1.94
39	0.98	12.9	10	0.44
42	27.4	3.15	10	5.39

a Permeability and efflux ratios determined in MDCK-MDR1 cells.

b Aqueous solubility at pH 7.4.

c Mouse liver microsome stability (half-life).

d Recovery low A2B; possible solubility or binding issues; accuracy of result questionable.

Compound 39 showed one of the most balanced profiles required for a cell-active probe: good permeability, no efflux and moderate solubility. This molecule shows good activity in the InCELL Pulse target engagement assay (PI5P4Kγ pIC_50_ = 7.3) and a good dual activity in the ADP-Glo assays showing similar activities for PI5P4Kγ+ and PI5P4Kα (fold-selectivity = 5). However, 39 has a very high turnover in MLMs and is unlikely to make a useful *in vivo* tool (MLM *t*^½^ = 0.44 min). Compound 39 (ARUK2007145) is therefore nominated as a PI5P4Kα/γ dual inhibitor probe molecule for cell experiments.

Other compounds of note are 32, which shows good ADMET properties in general, but is slightly less active than 39, particularly at PI5P4Kα, and the cluster of compounds 8, 15 and 37, which fall into the category of having a high MLM half-life and good PI5P4Kγ+ activity, but lower PI5P4Kα activity. All of these compounds have a 4-(methylsulfonyl)phenyl group at position *R*^2^. The polar methyl sulfonyl group is detrimental for PI5P4Kα activity but positively modulates microsomal stability, whereas more lipophilic groups in place of the methylsulfonyl group positively modulate PI5P4Kα activity but are metabolically liable. Nonetheless, compounds 8, 15 and 37, all have higher PI5P4Kα activities than previously reported compounds from this class^30^ and may make useful complementary probes.

## Discussion

**Fig. 3:**
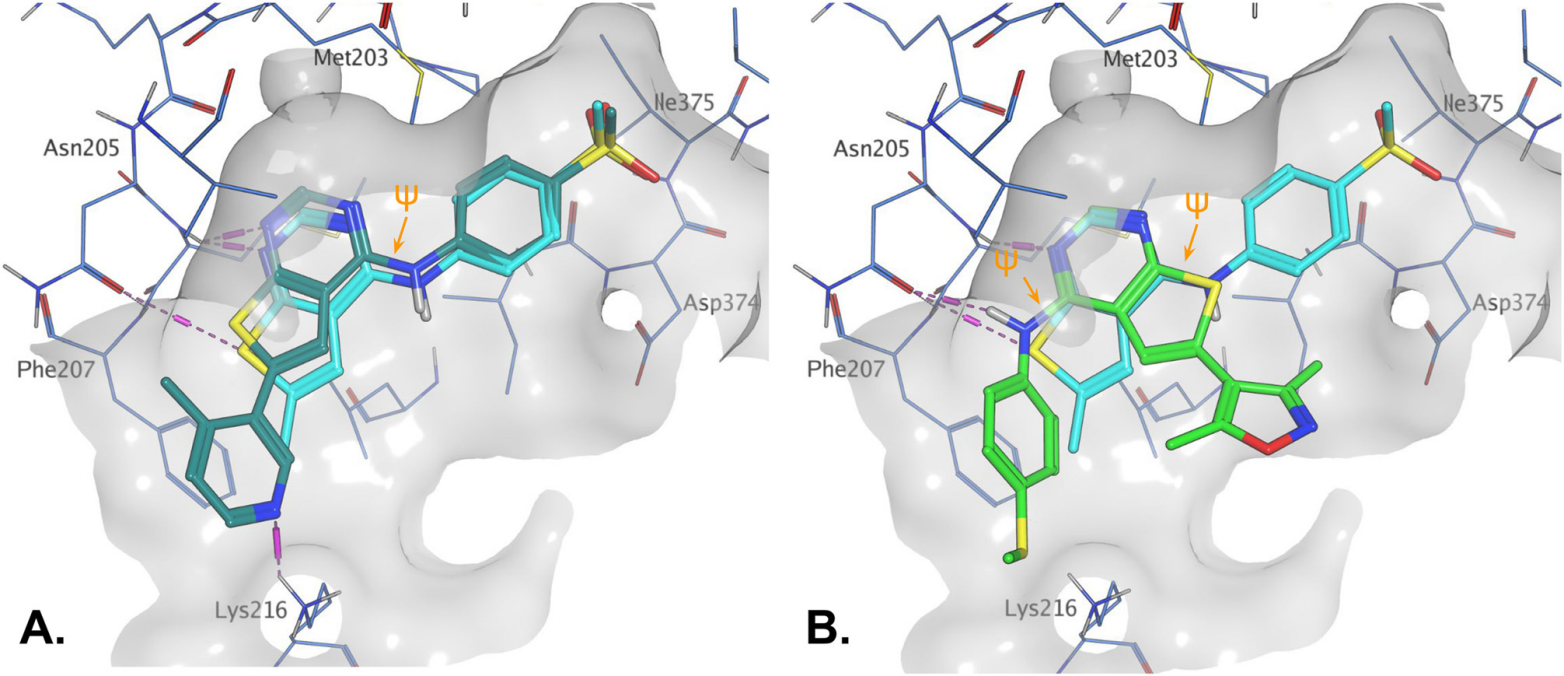
A) Compound 14 (teal); B) compound 30 (green) docked to PI5P4Kγ structure (8BQ4). Compound 1 shown for reference in both (cyan). The *ψ* dihedral angle is indicated in orange, this changes from 4° for 1 and 14 to 170° for 30.

Rational design of dual PI5P4Kα–PI5P4Kγ inhibitors such as 39 began with the observation that 5 offers a new vector for SAR exploration. Using the novel vector provided by the aryl ring at position 6 of 5's core, further iterations of design led to PI5P4Kα-active compounds with improved physicochemical properties over 5, particularly *X* log *P* (Table 2). For example, 14 is a more polar derivative which docked well in the PI5P4Kγ structure, 8BQ4 (Fig. 3A). However, not all compounds docked well in this orientation, *e.g.*30 shows different docking pose in 8BQ4 (Fig. 3B). The thioether of 30 appears to prefer a conformation where the aniline has rotated 180 degrees from the 8BQ4 orientation (Fig. 3B), so that the NH is pointing outwards. Thus, it is possible that the nature of the substituent on the aniline may influence the conformational preference at the *ψ* dihedral angle, and therefore also impact on the predicted binding pose.

**Fig. 4:**
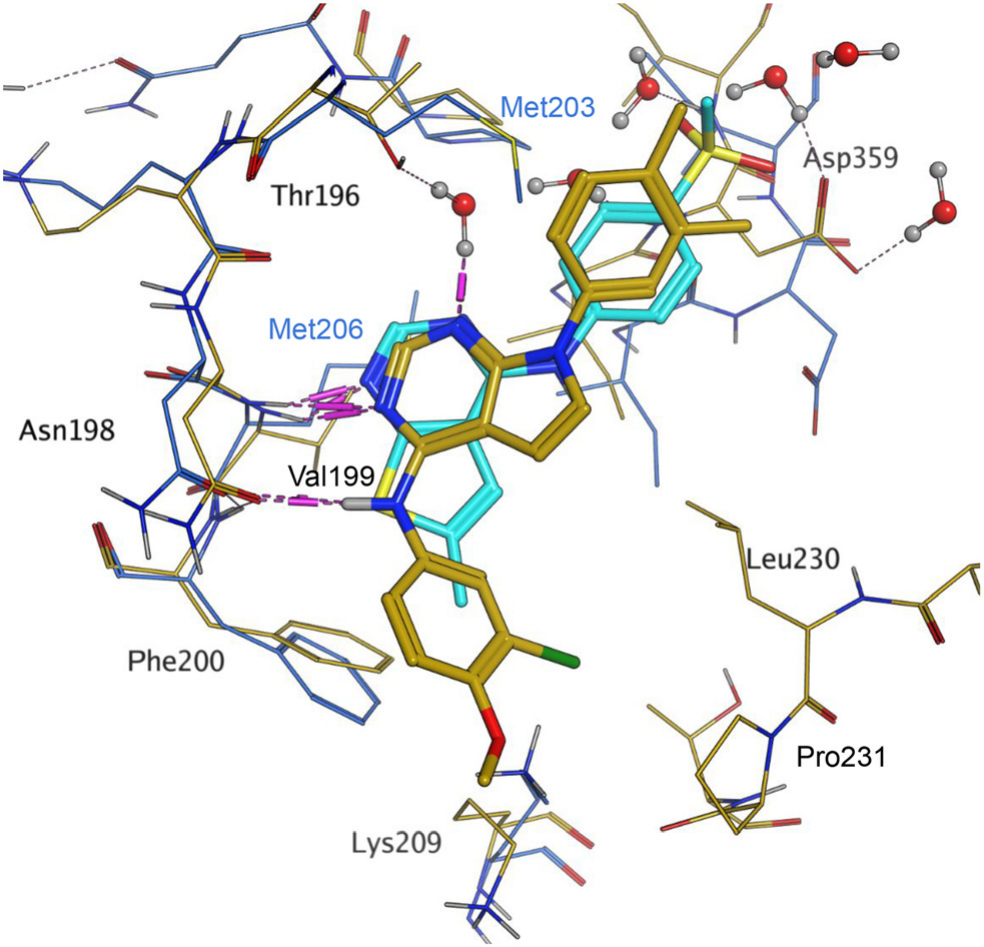
Compound 3 and PI5P4Kα protein (pdb 8C8C; gold) superimposed onto 1 and PI5P4Kγ protein (pdb 8BQ4; cyan), aligned using the hinge residues Thr196–Phe200 (numbering from PI5P4Kα sequence). This shows that the pyrimidine moiety does not bind in the same orientation in the PI5P4Kα and PI5P4Kγ ligands. PI5P4Kα residues are labelled in black and PI5P4Kγ residues in blue.

**Fig. 5:**
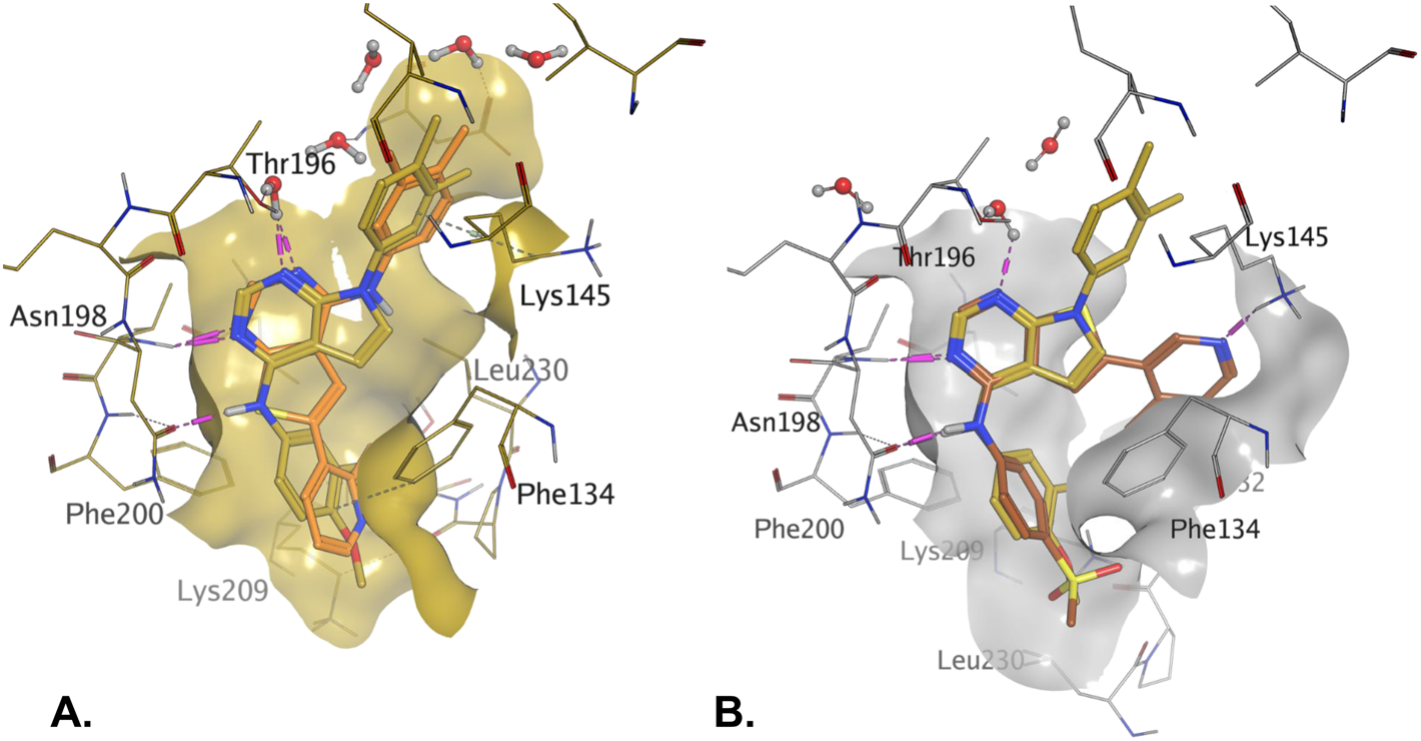
A) The dimethylphenyl group of 3 bound to PI5P4Kα (pdb 8C8C; gold) overlays well with that of 18 (orange) as positioned by docking to the 8C8C structure; B) 14 (brown) docked to alternative PI5P4Kα structure pdb 6YM4 (grey); 3 (pdb 8C8C; gold) was transposed onto the 6YM4 structure by alignment.

Later in the program, liganded PI5P4Kα crystal structures became available which allowed further structural assessment. The 8C8C PI5P4Kα structure shows ligand 3 in a binding mode in which, compared to 1 in the 8BQ4 PI5P4Kγ structure, has the aminopyrimidine flipped so that N2 rather than N4 is making an interaction with the hinge (Val199NH in PI5P4Kα, Met206 in PI5P4Kγ; Fig. 4). In the PI5P4Kα structure (8C8C) the aniline NH of 3 is also interacting with the hinge (Asn98OD1), whereas in the PI5P4Kγ structure (8BQ4) the aniline NH of 1 is not near the hinge residues. Retrospective docking suggested that our dual PI5P4Kα/PI5P4Kγ ligands 14 and 18 can interact with PI5P4Kα in two different poses, one is 1-like (Fig. 5A) and the other 3-like (Fig. 5B). For most of the dual inhibitors discussed here, the binding mode for 14 (shown in Fig. 5B) is only accessible in a protein conformation where Leu230 and Pro231 are not near the active site (seen, for example, in the PI5P4Kα structure solved with BAY297 bound, pdb 6YM4),^31^ as these two residues clash with the *R*^1^ extension in the 8C8C protein structure. Docking favours the pose shown in Fig. 5B for thieno[2,3-*d*] pyrimidines with an *R*^1^ 3-pyridine and an acceptor atom in *R*^2^, *e.g.*9, 14, 15, 16 and 17. This appears to be driven by interactions with Lys145 and Lys209. In contrast, the thieno[3,2-*d*]pyrimidines (Table 6) all adopt the pose shown in Fig. 5A in preference. This may stem from a lower conformational energy for the extended conformation shown in Fig. 5A for this core, or perhaps the thiophene C–H offers a better interaction with the side chain of Asn198 than the sulphur.

Interestingly, all of the ligands with good PI5P4Kα activities (pIC_50_ > 6.5), with the exception of 42, have a much better docking score in the PI5P4Kα–3 structure (8C8C) than the PI5P4Kα–BAY297 (6YM4) structure (see ESI† Table S1).

## Conclusions

The phosphatidylinositol 5-phosphate 4-kinases (PI5P4Ks) play a central role in regulating cell signalling pathways and, as such, have become therapeutic targets for diseases such as cancer, neurodegeneration and immunological disorders but useful probe molecules have only recently become available to further study these systems. To expand the tools available to the field, herein we disclose compound 39 and a series of related PI5P4Kα/γ dual inhibitors which were developed from a PI5P4Kγ-selective series, using rational approaches starting from PI5P4Kα-selective ligands and PI5P4Kγ-selective ligands. We anticipate that these tools will enable further discoveries in the field.

## Experimental details

### Biochemical assays

Assays to determine kinase activity of PI5P4Kα and PI5P4Kγ+ in the presence of inhibitors using an ADP-Glo assay (Promega) were performed as described previously.^38^ Recombinant PI5P4K protein was prepared as described previously.^8^*E. coli* BL21(DE3) clones harbouring PI5P4Kα (*PIP4K2A*; UniGene 138363), PI5P4Kβ (*PIP4K2B*; UniGene 171988) or PI5P4Kγ (*PIP4K2C*; UniGene 6280511), cloned into pGEX6P plasmid (Cytiva), were used to overexpress these proteins. PI5P4Kγ protein (designated “PI5P4Kγ+”) was a genetically modified chimera of PI5P4Kγ with a specific activity close to that of the active PI5P4Kα isoform^8^ and carries a number of PI5P4Kα-like mutations; insertion of three amino acids (QAR) at 139 plus an additional 11 amino acid mutations: S132L, E133P, S134N, E135D, G136S, D141G, G142A, E156T, N198G, E199G and D200E. Cultures were induced with 0.4 mM IPTG overnight and probe-sonicated in the presence of protease inhibitors. GST fusion protein was harvested using a GSTrap FF affinity column (Cytiva) and the GST tag removed *in situ* with 50 U of PreScission protease (Cytiva) for 4 h at 4 °C. The cleaved protein was further purified by size-exclusion chromatography (ÄKTA Pure, Cytiva). The protein purity was confirmed by sodium dodecyl sulfate–polyacrylamide gel electrophoresis, and the concentration was determined by colorimetric assay (Bio-Rad).

Binding of compounds to PI5P4Kγ in intact cells was assessed using an InCELL Pulse thermal stabilisation assay (DiscoverX) as described previously.^38^ PI5P4Kγ was cloned into the pICP vector (DiscoverX) to allow overexpression of the ePL-tagged target. Hek293 cells stably expressing ePL-tagged PI5P4Kγ were incubated with 25 nL of test compound in 100% DMSO in a black skirted PCR plate for 60 minutes at 38 °C. After incubation for 3 minutes at 46 °C, followed by cooling for 3 minutes at room temperature, 12 μL of EA-3 reagent (prepared as per the manufactures guidelines) was added to each well. The plate was then incubated for 60 minutes in the dark prior to luminescence reading on a Pherastar FSX plate reader (BMG Labtech).

### Data analysis

Statistical analysis was performed using nonparametric testing in Prism 8 (GraphPad). Activity pIC_50_ values and *in vivo* binding pEC_50_ values were estimated using a 4-parameter fit (Dotmatics).

### Computational modelling

The virtual screening procedure was performed as previously described.^30^ Dockings were performed using Glide SP (release 2022-3, Schrodinger, https://www.schrodinger.com). No constraints were used for docking. For 6YM4 docking the Lys209 conformation was changed to the rotamer that created the largest binding pocket using the MOE protein builder (release 22.02, Chemical Computing Group, https://www.chemcomp.com). Water molecules that are located in similar positions in 6YM4 and 8C8C were left in the site during docking. The structures shown in Fig. 1 and 3 resulted from minimization of the binding site residues after docking to allow H-bonds with lysines to form.

## Abbreviations

ADME: Absorption, distribution, metabolism, excretion and toxicity
ATP: Adenosine triphosphate
GTP: Guanosine triphosphate
MDCK-MDR1: Madin Darby canine kidney-multidrug resistance mutation 1
MLM: Mouse liver microsomes
*M*_W_
: Molecular weight
ND: Not determined
*P*_app_
: Apparent permeability coefficient
PI5P4K: Phosphatidylinositol 5-phosphate 4-kinase
QM: Quantum mechanics
SAR: Structure–activity relationship
WT: Wild-type
*X* Log *P*: Log partition coefficient

## Author contributions

The manuscript was written through contributions of all authors. All authors have given approval to the final version of the manuscript. Stephen Andrews and Jonathan Clarke were programme leaders, John Skidmore offered further project leadership; together all three designed the project plan. Gregory Aldred, Timothy Rooney and Helen Boffey designed and synthesised the compounds. Henriëtte Willems performed computational chemistry and docked compounds. David Winpenny and Christopher Green ran ADP-Glo and InCELL Pulse assays and screened the compounds.

## Conflicts of interest

There are no conflicts of interest to declare.

## Supplementary Material

MD-014-D3MD00355H-s001
