# Supplementary material for: The rational design of ARUK2007145, a dual inhibitor of the α and γ isoforms of the lipid kinase phosphatidylinositol 5-phosphate 4-kinase (PI5P4K)
Source: RSC Med Chem. 2023 Aug 23;14(10):2035–47. doi: 10.1039/d3md00355h (PMC10583824; doi:10.1039/d3md00355h)
Supplement: MD-014-D3MD00355H-s001 [file MD-014-D3MD00355H-s001.pdf]

Electronic Supplementary Material (ESI) for RSC Medicinal Chemistry.
This journal is © The Royal Society of Chemistry 2023

Supporting information

The Rational Design of ARUK2007145, a Dual Inhibitor of the α and γ Isoforms of the Lipid Kinase Phosphatidylinositol 5-phosphate 4-kinase (PI5P4K)

Gregory G. Aldred,† Timothy P. C. Rooney,† Henriette M. G. Willems, Helen K. Boffey, Christopher Green, David Winpenny, John Skidmore, Jonathan H. Clarke, Stephen P. Andrews*

Affiliations

The ALBORADA Drug Discovery Institute, University of Cambridge, Island Research Building, Cambridge Biomedical Campus, Hills Road, Cambridge, CB2 0AH, United Kingdom

Corresponding Author

*E-mail: spa26@cam.ac.uk

Table of contents

	Page
Abbreviations	S2
Chemical synthesis	S3
General experimental	S3
General and example schemes	S4
General procedures 1, 2 and 3	S4
Synthesis procedures	S5
Spectra for key compounds **8, 10, 14, 15, 17, 26, 32, 37, 38, 39 and 42**	S31
**Table S1.** Glide docking scores.	S37
ADMET experimental methods	S39

Abbreviations: DCM: dichloromethane, DiPEA: diisopropylethylamine, DME: 1,2-dimethoxyethane, eq: equivalents, DMSO: dimethylsulfoxide, ESI: electrospray ionisation, HPLC: high-performance liquid chromatography IPA: *iso*-propanol, MeCN: acetonitrile, MeOH: methanol, min: minutes, MIDA: methyliminodiacetic acid, MS: mass spectrometry, NMR: nuclear magnetic resonance, rt: room temperature, SCX: strong cation exchange, UPLC: ultra high-performance liquid chromatography.

Chemical Synthesis

General Experimental

Reagents and solvents were of commercially available reagent grade quality and used without further purification. Reactions requiring anhydrous conditions were carried out in oven dried glassware under an atmosphere of N_2. Reactions were monitored by thin-layer chromatography (TLC) on silica gel 60 F_{254} aluminium or glass supported sheets, or by liquid chromatography-mass spectrometry (LC-MS). Flash column chromatography was carried out on a Biotage Isolera One system using normal phase (SiO_2) or reverse phase (C18) cartridges. Compounds were loaded in solution or adsorbed onto Celite® 545 or ISOLUTE® HM-N and eluted using a linear gradient of the specified solvents. Purification by C18 reverse phase HPLC was carried using an Agilent 1260 Infinity machine and a Waters XBridge BEH C18 OBD column (130 Å, 5 µm, 30 mm × 100 mm) with a linear gradient of H_2O (with 0.1% NH_3) and MeCN (with 0.1% NH_3). LCMS analysis was performed on a Waters Aquity HClass UPLC system with a Aquity QDa for mass detection. NMR spectra were recorded on a Bruker Avance III (^1H = 300 MHz, ^{19}F = 282 MHz) or a Bruker Avance III with Dual $^{13}C/^1H$ Cryoprobe (^{13}C = 126 MHz) spectrometer using the requisite solvent as a reference for internal deuterium lock. The chemical shift data for each signal are given as δ chemical shift (multiplicity, *J* values in Hz, integration) in units of parts per million (ppm) relative to tetramethylsilane (TMS) where δH (TMS) = 0.00 ppm. The multiplicity of each signal is indicated by: s (singlet), d (doublet), t (triplet), q (quartet), quin (quintet), or m (multiplet). Signals from exchangeable protons are not always detected. UPLC analysis of final compounds was performed on a Waters Aquity HClass UPLC system and is reported as method name, retention time, UV % purity. The method parameters are as follows:

Method	Column	Additive	Flow rate	Gradient (time, %MeCN in H_2O)
A	BEH C18 (130 Å, 1.7 µm, 2.1 mm × 50 mm)	10 mM NH_3	0.6 mL/min	0 min, 5%; 0.8 min, 5%; 3.3 min, 95%; 4.3 min, 95%; 4.5 min, 5%; 5.5 min, 5%.
B	HSS C18 (100 Å, 1.8 µm, 2.1 mm × 50 mm)	0.1% HCO_2H	0.6 mL/min	0 min, 5%; 0.8 min, 5%; 3.3 min, 95%; 4.3 min, 95%; 4.5 min, 5%; 5.5 min, 5%.
C	BEH C18 (130 Å, 1.7 µm, 2.1 mm × 50 mm)	10 mM NH_3	0.6 mL/min	0 min, 5%; 0.8 min, 5%; 8.3 min, 95%; 9.3 min, 95%; 9.5 min, 5%; 10.5 min, 5%.
D	HSS C18 (100 Å, 1.8 µm, 2.1 mm × 50 mm)	0.1% HCO_2H	0.6 mL/min	0 min, 5%; 0.8 min, 5%; 8.3 min, 95%; 9.3 min, 95%; 9.5 min, 5%; 10.5 min, 5%.
E	BEH C18 (130 Å, 1.7 µm, 2.1 mm × 50 mm)	1 mM NH_3	0.6 mL/min	0 min, 5%; 0.8 min, 5%; 3.3 min, 95%; 4.3 min, 95%; 4.5 min, 5%; 5.5 min, 5%.
F	BEH C18 (130 Å, 1.7 µm, 2.1 mm × 50 mm)	1 mM NH_3	0.6 mL/min	0 min, 5%; 0.8 min, 5%; 8.3 min, 95%; 9.3 min, 95%; 9.5 min, 5%; 10.5 min, 5%.

Scheme 1:

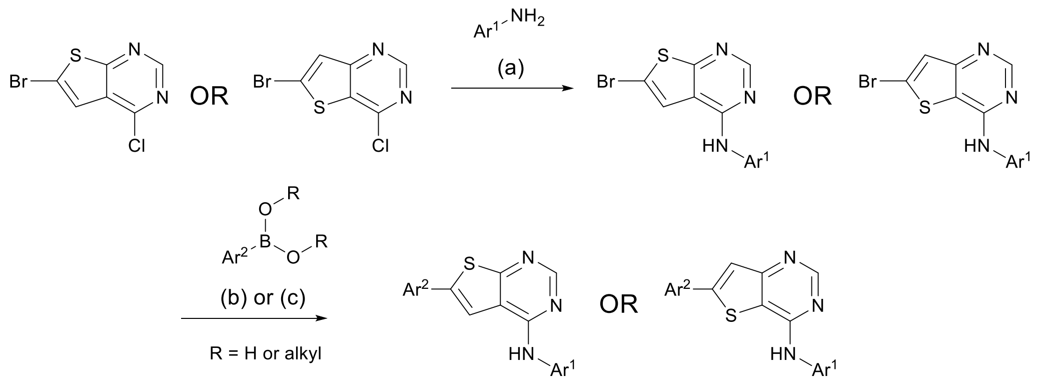

General scheme. Reagents and conditions: (a) Aniline, IPA, 120 °C μW, 1-2 h. (b) boronic acid/ester, $Pd(dppf)Cl_2 \cdot CH_2Cl_2$, DME, 120 °C μW, 30 min or (c) boronic acid/ester, $Pd(PPh_3)_4$, dioxane:H_2O, 100 °C, 16 h.

Scheme 2:

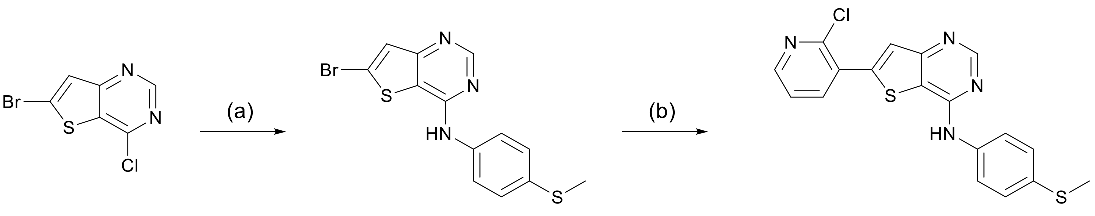

Example scheme for key compound **39.** Reagents and conditions: (a) 4-(methylthio)aniline, IPA, 120 °C μW, 2 h. (b) (2-chloropyridin-3-yl)boronic acid, $Pd(dppf)Cl_2 \cdot CH_2Cl_2$, DME, 120 °C μW, 30 min.

General procedure 1: Microwave Suzuki coupling

A microwave flask was charged with 6-bromo-N-(4-methylsulfonylphenyl)thieno[2,3-*d*]pyrimidin-4-amine (**43**) (1.0 eq), dichloro[1,1'-bis(diphenylphosphino)ferrocene]palladium(II), complex with dichloromethane (1:1) (0.1 eq), potassium carbonate (4.0 eq) and requisite boronic acid/ester (2.5 eq). The flask sealed and contents suspended in 1,4-dioxane (Caution! Carcinogenic):water (3:1, 0.2 M) before heating at 120 °C in a microwave (Biotage Initiator+) for the stated time. The reaction was cooled and placed on to an SCX-II cartridge, washed with DCM (Caution! Carcinogenic), followed by MeOH before eluting with 0.5 N methanolic ammonia. The methanolic ammonia fraction was concentrated *in vacuo* and purified by the stated method.

General procedure 2: Thermal Suzuki coupling

A microwave flask was charged with 6-bromo-N-(4-methylsulfonylphenyl)thieno[2,3-*d*]pyrimidin-4-amine (**43**) (1.0 eq), tetrakis(triphenylphosphine)palladium (0.1 eq), potassium carbonate (4.0 eq) and requisite boronic acid/ester (2.0 eq). The flask sealed and contents suspended in 1,4-dioxane (Caution! Carcinogenic):water (3:1, 0.2 M) before heating at 100 °C thermally for 16 hours. The reaction was cooled and placed on to an SCX-II cartridge, washed with DCM (Caution! Carcinogenic), followed by MeOH before eluting with 0.5 N methanolic ammonia. The methanolic ammonia fraction was concentrated *in vacuo* and purified by the stated method.

General procedure 3: Microwave chloro displacement

A suspension of 4-chloro-6-(2-methyl-3-pyridyl)thieno[2,3-*d*]pyrimidine (1.0 eq) and requisite aniline (1.0 eq) in IPA (0.14 M) was sealed in a microwave flask and heating at stated temperature in a microwave (Biotage Initiator+) for 30 mins. The reaction was cooled, a small amount of DMSO added and purified by preparative HPLC (elution gradient 40-80% of MeCN in H_2O with 0.1% NH_3).

6-bromo-*N*-(4-methylsulfonylphenyl)thieno[2,3-*d*]pyrimidin-4-amine (43)

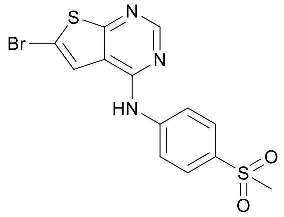

To a suspension of 6-bromo-4-chloro-thieno[2,3-*d*]pyrimidine (0.50 g, 2.00 mmol) in 4 M HCl in 1,4-dioxane (Caution! Carcinogenic; 32 mL, 127.6 mmol) was added 4-(methylsulfonyl)aniline hydrochloride (0.42 g, 2.00 mmol) and the mixture was stirred at 100 °C for 40 hours. Reaction mixture was cooled to rt and diluted with EtOAc, washed with $NaHCO_3$ (x 1) and water (x2). The organic layer was then dried over Mg_2SO_4 and washed through with MeOH/DCM (Caution! Carcinogenic), combined washes were concentrated in vacuo and the crude material purified by flash column chromatography (elution gradient 20-100% EtOAc in petroleum ether) yielding 6-bromo-*N*-(4-methylsulfonylphenyl)thieno[2,3-*d*]pyrimidin-4-amine **43** (311 mg, 0.809 mmol, 40% yield). MS (ESI+) *m/z* calcd for $C_{13}H_{10}BrN_3O_2S_2^+$ $[M+H]^+$ 383.9 and 385.9, found 384.1 and 386.1. ^1H NMR (300 MHz, DMSO-d_6) δ 9.99 (s, 1H), 8.62 (s, 1H), 8.20 – 8.08 (m, 3H), 7.99 – 7.87 (m, 2H), 3.20 (s, 3H). UPLC analysis (method C), 4.58 min, >98% purity.

N-(4-methylsulfonylphenyl)-6-(4-pyridyl)thieno[2,3-d]pyrimidin-4-amine (6)

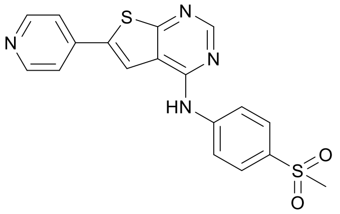

6-bromo-N-(4-methylsulfonylphenyl)thieno[2,3-*d*]pyrimidin-4-amine **43** (30.0 mg, 0.078 mmol) and 4-pyridylboronic acid hydrate (16.5 mg, 0.120 mmol) were reacted together following general procedure 2. Crude material purified by flash column chromatography (elution gradient 0-10% MeOH in DCM (Caution! Carcinogenic)) yielding *N*-(4-methylsulfonylphenyl)-6-(4-pyridyl)thieno[2,3-*d*]pyrimidin-4-amine **6** (7.7 mg, 0.020 mmol, 34% yield). MS (ESI+) *m/z* calcd for $C_{18}H_{14}N4O_2S_2^+$ $[M + H]^+$ 383.1, found 383.3. ^{1}H NMR (300 MHz, DMSO-d_6) δ 10.19 (s, 1H), 8.73 (d, *J* = 6.1 Hz, 2H), 8.68 (s, 1H), 8.60 (s, 1H), 8.19 (d, *J* = 8.9 Hz, 2H), 7.96 (d, *J* = 8.8 Hz, 2H), 7.73 (d, *J* = 6.2 Hz, 2H), 3.22 (s, 3H). UPLC analysis (method C), 4.15 min, 97% purity.

N-(4-methylsulfonylphenyl)-6-(1H-pyrazol-4-yl)thieno[2,3-d]pyrimidin-4-amine (7)

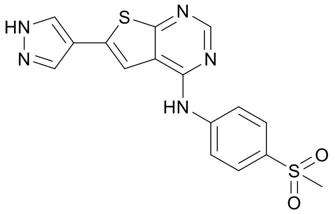

6-bromo-N-(4-methylsulfonylphenyl)thieno[2,3-*d*]pyrimidin-4-amine **43** (37.0 mg, 0.1 mmol) and 1-Boc-4-(4,4,5,5-tetramethyl-1,3,2-dioxaborolan-2-yl)pyrazole (70.8 mg, 0.24 mmol) were reacted together following general procedure 1 for 1 hour. Crude material purified by preparative HPLC (elution gradient 5-95% of MeCN in H_2O with 0.1% NH_3) yielding *N*-(4-methylsulfonylphenyl)-6-(1H-pyrazol-4-yl)thieno[2,3-*d*]pyrimidin-4-amine **7** (9.1 mg, 0.024 mmol, 25% yield). MS (ESI+) *m/z* calcd for $C_{16}H_{13}N_5O_2S_2^+$ $[M + H]^+$ 372.1, found 372.3. ^{1}H NMR (300 MHz, DMSO-d_6) δ 8.55 (s, 1H), 8.21 – 8.13 (m, 2H), 8.09 (s, 2H), 7.96 – 7.87 (m, 3H) and 3.20 (s, 3H). UPLC analysis (method C), 3.72 min, >98% purity.

6-(1-methylpyrazol-4-yl)-*N*-(4-methylsulfonylphenyl)thieno[2,3-*d*]pyrimidin-4-amine (8)

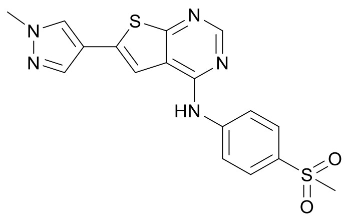

6-bromo-N-(4-methylsulfonylphenyl)thieno[2,3-*d*]pyrimidin-4-amine **43** (30.0 mg, 0.078 mmol) and 1-methylpyrazole-4-boronic acid pinacol ester (24.4 mg, 0.120 mmol) were reacted together following general procedure 2. Crude material purified by preparative HPLC (elution gradient 5-95% of MeCN in H_2O with 0.1% NH_3) yielding 6-(1-methylpyrazol-4-yl)-*N*-(4-methylsulfonylphenyl)thieno[2,3-*d*]pyrimidin-4-amine **8** (7.6 mg, 0.020 mmol, 34% yield). MS (ESI+) *m/z* calcd for $C_{17}H_{15}N_5O_2S_2^+$ $[M + H]^+$ 386.1, found 386.4. ^1H NMR (300 MHz, DMSO-*d*$_6$) δ 10.00 (bs, 1H), 8.56 (s, 1H), 8.25 – 8.09 (m, 3H), 7.97 – 7.88 (m, 3H), 7.86 (d, *J* = 0.8 Hz, 1H), 3.92 (s, 3H), and 3.20 (s, 3H). UPLC analysis (method C), 4.06 min, >98% purity.

***N*-(4-methylsulfonylphenyl)-6-(3-pyridyl)thieno[2,3-*d*]pyrimidin-4-amine (9)**

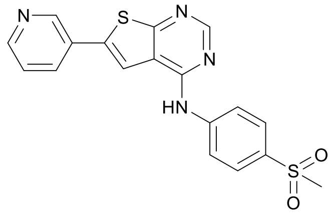

6-bromo-N-(4-methylsulfonylphenyl)thieno[2,3-*d*]pyrimidin-4-amine **43** (30.0 mg, 0.078 mmol) and pyridine-3-boronic acid (19.2 mg, 0.156 mmol) were reacted together following general procedure 2. Crude material purified by preparative HPLC (elution gradient 5-95% of MeCN in H_2O with 0.1% NH_3) yielding *N*-(4-methylsulfonylphenyl)-6-(3-pyridyl)thieno[2,3-*d*]pyrimidin-4-amine **9** (6.1 mg, 0.016 mmol, 20% yield). MS (ESI+) *m/z* calcd for $C_{18}H_{14}N4O_2S_2^+$ $[M + H]^+$ 383.1, found 383.3. ^1H NMR (300 MHz, DMSO-*d*$_6$) δ 9.00 (dd, *J* = 2.4, 0.8 Hz, 1H), 8.63 (dd, *J* = 4.7, 1.5 Hz, 1H), 8.60 (s, 1H), 8.39 (s, 1H), 8.19 – 8.10 (m, 3H), 7.93 (d, *J* = 8.8 Hz, 2H), 7.58 (dd, *J* = 7.7, 4.7 Hz, 1H), and 3.20 (s, 3H). UPLC analysis (method C), 4.20 min, >98% purity.

6-(3-methyl-4-pyridyl)-*N*-(4-methylsulfonylphenyl)thieno[2,3-*d*]pyrimidin-4-amine (10)

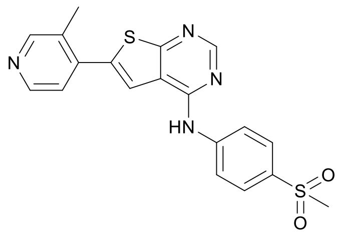

6-bromo-N-(4-methylsulfonylphenyl)thieno[2,3-*d*]pyrimidin-4-amine **43** (60.0 mg, 0.156 mmol) and (3-methylpyridin-4-yl)boronic acid (42.8 mg, 0.310 mmol) were reacted together following general procedure 1 for 1 hour. Crude material purified by preparative HPLC (elution gradient 5-95% of MeCN in H_2O with 0.1% NH_3) yielding 6-(3-methyl-4-pyridyl)-*N*-(4-methylsulfonylphenyl)thieno[2,3-*d*]pyrimidin-4-amine **10** (28.0 mg, 0.071 mmol, 45% yield) as a brown solid. MS (ESI+) *m/z* calcd for $C_{19}H_{16}N_4O_2S_2^+$ $[M + H]^+$ 397.1, found 397.3. ^1H NMR (300 MHz, DMSO-d_6) δ 10.24 (s, 1H), 8.68 (s, 2H), 8.59 (s, 1H), 8.33 (s, 1H), 8.19 (d, J = 8.9 Hz, 2H), 7.96 (d, J = 8.9 Hz, 2H), 7.62 (d, J = 5.0 Hz, 1H), 3.22 (s, 3H), 2.59 (s, 3H).. UPLC analysis (method C), 4.35 min, >98% purity.

6-(3-methoxy-4-pyridyl)-*N*-(4-methylsulfonylphenyl)thieno[2,3-*d*]pyrimidin-4-amine (11)

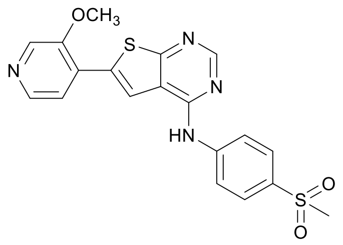

6-bromo-N-(4-methylsulfonylphenyl)thieno[2,3-*d*]pyrimidin-4-amine **43** (30.0 mg, 0.080 mmol) and 3-methoxy-4-(4,4,5,5-tetramethyl-1,3,2-*d*ioxaborolan-2-yl)pyridine (27.5 mg, 0.120 mmol) were reacted together following general procedure 1 for 30 mins. Crude material purified by preparative HPLC (elution gradient 5-95% of MeCN in H_2O with 0.1% NH_3) yielding (3-methoxy-4-pyridyl)-*N*-(4-methylsulfonylphenyl)thieno[2,3-*d*]pyrimidin-4-amine **11** (11.0 mg, 0.027 mmol, 34% yield) as a white solid. MS (ESI+) *m/z* calcd for $C_{19}H_{16}N_4O_3S_2^+$ $[M + H]^+$ 413.1, found 413.2. ^1H NMR (300 MHz, DMSO-d_6) δ 10.12 (s, 1H), 8.65 (s, 1H), 8.65 (s, 1H), 8.61 (s, 1H), 8.38 (d, J = 5.0 Hz, 1H), 8.23 – 8.13 (m, 2H), 8.01 – 7.88 (m, 2H), 7.76 (d, J = 5.1 Hz, 1H), 4.13 (s, 3H) and 3.21 (s, 3H). UPLC analysis (method C), 4.32 min, >98% purity.

6-(3-fluoro-4-pyridyl)-*N*-(4-methylsulfonylphenyl)thieno[2,3-*d*]pyrimidin-4-amine (12)

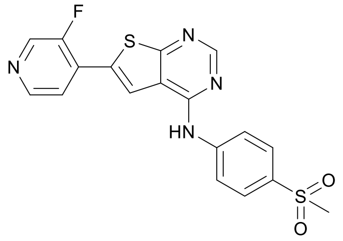

6-bromo-N-(4-methylsulfonylphenyl)thieno[2,3-*d*]pyrimidin-4-amine **43** (30.0 mg, 0.078 mmol) and 3-fluoro-4-(4,4,5,5-tetramethyl-1,3,2-*d*ioxaborolan-2-yl)pyridine (26.1 mg, 0.120 mmol) were reacted together following general procedure 1 for 90 mins. Crude material purified by preparative HPLC (elution gradient 40-80% of MeCN in H_2O with 0.1% NH_3) yielding 6-(3-fluoro-4-pyridyl)-*N*-(4-methylsulfonylphenyl)thieno[2,3-*d*]pyrimidin-4-amine **12** (8.0 mg, 0.020 mmol, 26% yield) as a white solid. MS (ESI+) *m/z* calcd for $C_{18}H_{13}FN_4O_2S_2^+$ $[M + H]^+$ 401.1, found 401.2. ^{1}H NMR (300 MHz, DMSO-*d*$_6$) δ 8.76 (d, *J* = 3.1 Hz, 1H), 8.58 (s, 1H), 8.55 (s, 1H), 8.54 (d, *J* = 3.9 Hz, 1H), 8.05 (d, *J* = 8.6 Hz, 2H), 7.91 – 7.86 (m, 2H), 7.84 (dd, *J* = 7.0, 5.1 Hz, 1H), 3.19 (s, 3H), exchangeable N*H* signal not observed. 19F NMR (282 MHz, DMSO-*d*$_6$) δ -129.3. UPLC analysis (method C), 4.43 min, >98% purity.

6-(2-chloro-4-pyridyl)-*N*-(4-methylsulfonylphenyl)thieno[2,3-*d*]pyrimidin-4-amine (**13**)

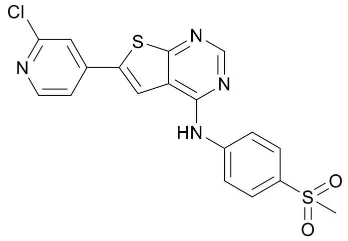

6-bromo-N-(4-methylsulfonylphenyl)thieno[2,3-*d*]pyrimidin-4-amine **43** (30.0 mg, 0.080 mmol) and (2-chloropyridin-4-yl)boronic acid (25.2 mg, 0.160 mmol) were reacted together following general procedure 1 for 90 mins. Crude material purified by preparative HPLC (elution gradient 40-80% of MeCN in H_2O with 0.1% NH_3) yielding 6-(2-chloro-4-pyridyl)-*N*-(4-methylsulfonylphenyl)thieno[2,3-*d*]pyrimidin-4-amine **13** (18.0 mg, 0.043 mmol, 55% yield) as an off-white solid. MS (ESI+) *m/z* calcd for $C_{18}H_{13}ClN_4O_2S_2^+$ $[M + H]^+$ 417.0, found 417.2. ^{1}H NMR (300 MHz, DMSO-*d*$_6$) δ 8.62 (d, *J* = 4.9 Hz, 2H), 8.53 (d, *J* = 5.3 Hz, 1H), 8.11 (d, *J* = 8.8 Hz, 2H), 7.93 (d, *J* = 8.8 Hz, 2H), 7.82 (d, *J* = 1.6 Hz, 1H), 7.72 (dd, *J* = 5.3, 1.6 Hz, 1H) and 3.21 (s, 3H), exchangeable N*H* signal not observed. UPLC analysis (method D), 5.04 min, 97% purity.

6-(4-methyl-3-pyridyl)-*N*-(4-methylsulfonylphenyl)thieno[2,3-*d*]pyrimidin-4-amine (14)

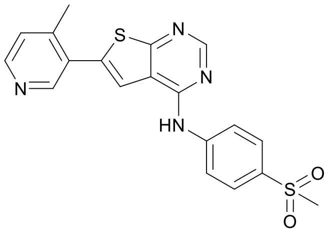

6-bromo-N-(4-methylsulfonylphenyl)thieno[2,3-*d*]pyrimidin-4-amine **43** (30.0 mg, 0.080 mmol) and (4-methylpyridin-3-yl)boronic acid (16.0 mg, 0.120 mmol) were reacted together following general procedure 1 for 60 mins. Crude material purified by preparative HPLC (elution gradient 5-95% of MeCN in H_2O with 0.1% NH_3) yielding 6-(4-methyl-3-pyridyl)-*N*-(4-methylsulfonylphenyl)thieno[2,3-*d*]pyrimidin-4-amine **14** (29.0 mg, 0.073 mmol, 94% yield) as a white solid. MS (ESI+) *m/z* calcd for $C_{19}H_{16}N_4O_2S_2^+$ $[M + H]^+$ 397.1, found 397.2. ^1H NMR (300 MHz, DMSO-d_6) δ 10.09 (s, 1H), 8.70 (s, 1H), 8.67 (s, 1H), 8.53 (d, J = 5.0 Hz, 1H), 8.22 – 8.14 (m, 2H), 8.10 (s, 1H), 7.99 – 7.91 (m, 2H), 7.47 (dt, J = 5.0, 0.7 Hz, 1H), 3.21 (s, 3H), and 2.54 (s, 3H). UPLC analysis (method F), 4.33 min, 97% purity.

6-(2-chloro-3-pyridyl)-*N*-(4-methylsulfonylphenyl)thieno[2,3-*d*]pyrimidin-4-amine (15)

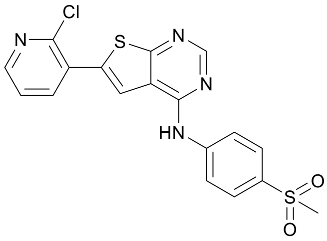

6-bromo-N-(4-methylsulfonylphenyl)thieno[2,3-*d*]pyrimidin-4-amine **43** (30.0 mg, 0.080 mmol) and (2-chloropyridin-3-yl)boronic acid (18.4 mg, 0.120 mmol) were reacted together following general procedure 1 for 180 mins. Crude material purified by preparative HPLC (elution gradient 40-80% of MeCN in H_2O with 0.1% NH_3) yielding 6-(2-chloro-3-pyridyl)-*N*-(4-methylsulfonylphenyl)thieno[2,3-*d*]pyrimidin-4-amine **15** (20.0 mg, 0.048 mmol, 62% yield) as a white solid. MS (ESI+) *m/z* calcd for $C_{18}H_{13}ClN_4O_2S_2^+$ $[M + H]^+$ 417.0, found 417.2. ^1H NMR (300 MHz, DMSO-d_6) δ 8.59 (s, 1H), 8.51 (dd, J = 4.7, 1.9 Hz, 1H), 8.28 (s, 1H), 8.21 (dd, J = 7.7, 1.9 Hz, 1H), 8.09 (d, J = 8.7 Hz, 2H), 7.94 – 7.86 (m, 2H), 7.62 (dd, J = 7.7, 4.7 Hz, 1H) and 3.19 (s, 3H). UPLC analysis (method D), 4.67 min, 98% purity.

6-(2-fluoro-3-pyridyl)-*N*-(4-methylsulfonylphenyl)thieno[2,3-*d*]pyrimidin-4-amine (16)

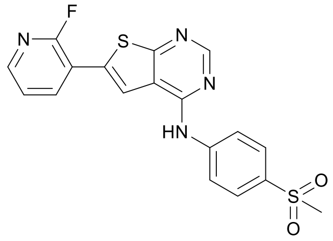

6-bromo-N-(4-methylsulfonylphenyl)thieno[2,3-*d*]pyrimidin-4-amine **43** (30.0 mg, 0.080 mmol) and 2-fluoropyridine-3-boronic acid (16.5 mg, 0.120 mmol) were reacted together following general procedure 1 for 90 mins. Crude material purified by preparative HPLC (elution gradient 40-80% of MeCN in H_2O with 0.1% NH_3) yielding 6-(2-fluoro-3-pyridyl)-*N*-(4-methylsulfonylphenyl)thieno[2,3-*d*]pyrimidin-4-amine **16** (2.0 mg, 0.005 mmol, 6% yield) as a white solid. MS (ESI+) *m/z* calcd for $C_{18}H_{13}FN_4O_2S_2^+$ $[M + H]^+$ 401.1, found 401.2. ^1H NMR (300 MHz, DMSO-d_6) δ 10.42 (s, 1H), 8.68 (d, *J* = 14.1 Hz, 2H), 8.39 (ddd, *J* = 9.8, 7.6, 1.9 Hz, 1H), 8.31 (dt, *J* = 4.9, 1.5 Hz, 1H), 8.28 – 8.19 (m, 2H), 7.99 – 7.89 (m, 2H), 7.58 (ddd, *J* = 7.3, 4.8, 1.9 Hz, 1H), 3.21 (s, 3H). UPLC analysis (method C), 4.62 min, >98% purity.

6-(2-methyl-3-pyridyl)-*N*-(4-methylsulfonylphenyl)thieno[2,3-*d*]pyrimidin-4-amine (17)

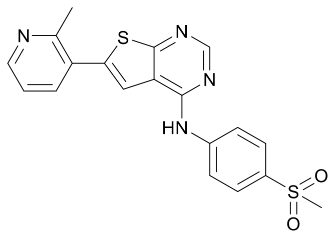

6-bromo-N-(4-methylsulfonylphenyl)thieno[2,3-*d*]pyrimidin-4-amine **43** (30.0 mg, 0.080 mmol) and (2-methylpyridin-3-yl)boronic acid (21.4 mg, 0.160 mmol) were reacted together following general procedure 1 for 60 mins. Crude material purified by preparative HPLC (elution gradient 5-95% of MeCN in H_2O with 0.1% NH_3) yielding 6-(2-methyl-3-pyridyl)-*N*-(4-methylsulfonylphenyl)thieno[2,3-*d*]pyrimidin-4-amine **17** (9.1 mg, 0.023 mmol, 29% yield). MS (ESI+) *m/z* calcd for $C_{19}H_{16}N_4O_2S_2^+$ $[M + H]^+$ 397.1, found 397.2. ^1H NMR (300 MHz, DMSO-d_6) δ 10.05 (bs, 1H), 8.65 (s, 1H), 8.56 (dd, *J* = 4.8, 1.7 Hz, 1H), 8.20 – 8.13 (m, 2H), 8.08 (s, 1H), 7.98 – 7.89 (m, 3H), 7.41 (dd, *J* = 7.8, 4.8 Hz, 1H), 3.21 (s, 3H), 2.72 (s, 3H). UPLC analysis (method C), 4.31 min, >98% purity.

4-Chloro-6-(2-methyl-3-pyridyl)thieno[2,3-*d*]pyrimidine (44)

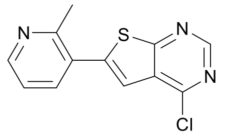

6-Bromo-4-chlorothieno[2,3-*d*]pyrimidine (350 mg, 1.40 mmol), dichloro[1,1'-bis(diphenylphosphino) ferrocene]palladium(II), complex with dichloromethane (1:1) (57.4 mg, 0.070 mmol), potassium carbonate (582 mg, 4.21 mmol) and (2-methylpyridin-3-yl)boronic acid (230 mg, 1.68 mmol) were suspended in 1,2-dimethoxyethane (10.5 mL) and water (1.5 mL) before heating for 1 hour at 120 °C. The crude reaction mixture was passed through sodium sulfate to remove the water before washing with EtOAc. Resulting solution was dried under reduced pressure to yield 4-chloro-6-(2-methyl-3-pyridyl)thieno[2,3-*d*]pyrimidine **44** (300 mg, 1.15 mmol, 82% yield) as a black solid and carried forward without further purification. MS (ESI+) *m/z* calcd for $C_{12}H_{8}ClN_{3}S^{+}$ $[M + H]^{+}$ 262.1, found 262.2. UPLC analysis (method E), 2.82 min, 75% purity.

***N*-(3,4-dimethylphenyl)-6-(2-methyl-3-pyridyl)thieno[2,3-*d*]pyrimidin-4-amine (18)**

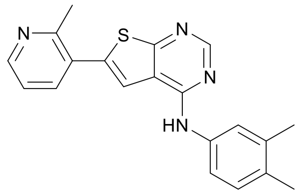

4-Chloro-6-(2-methyl-3-pyridyl)thieno[2,3-*d*]pyrimidine **44** (30.0 mg, 0.080 mmol) and 3,4-dimethylaniline (9.72 mg, 0.080 mmol) were reacted following general procedure 3 at 140 °C to yield *N*-(3,4-dimethylphenyl)-6-(2-methyl-3-pyridyl)thieno[2,3-*d*]pyrimidin-4-amine **18** (10.5 mg, 0.030 mmol, 38% yield). MS (ESI+) *m/z* calcd for $C_{20}H_{18}N_{4}S^{+}$ $[M + H]^{+}$ 347.1, found 347.2. ^{1}H NMR (300 MHz, DMSO-d_6) δ 9.61 (s, 1H), 8.54 (dd, *J* = 4.8, 1.7 Hz, 1H), 8.49 (s, 1H), 8.01 (s, 1H), 7.90 (dd, *J* = 7.8, 1.7 Hz, 1H), 7.60 – 7.52 (m, 2H), 7.39 (dd, *J* = 7.8, 4.8 Hz, 1H), 7.15 (d, *J* = 8.0 Hz, 1H), 2.71 (s, 3H), 2.25 (s, 3H), 2.22 (s, 3H). UPLC analysis (method F), 3.59 min, >98% purity.

N-(2,4-dimethylphenyl)-6-(2-methyl-3-pyridyl)thieno[2,3-d]pyrimidin-4-amine (19)

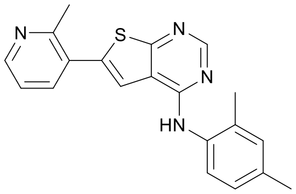

4-Chloro-6-(2-methyl-3-pyridyl)thieno[2,3-*d*]pyrimidine **44** (30.0 mg, 0.080 mmol) and 2,4-xylidine (19.5 mg, 0.160 mmol) were reacted following general procedure 3 at 140 °C to yield *N*-(2,4-dimethylphenyl)-6-(2-methyl-3-pyridyl)thieno[2,3-*d*]pyrimidin-4-amine **19** (1.70 mg, 0.049 mmol, 62% yield). MS (ESI+) *m/z* calcd for $C_{20}H_{18}N_4S^+$ $[M + H]^+$ 347.1, found 347.2. ^{1}H NMR (300 MHz, DMSO-*d*$_6$) δ 9.50 (s, 1H), 8.53 (dd, *J* = 4.8, 1.7 Hz, 1H), 8.32 (s, 1H), 7.89 (dd, *J* = 7.8, 1.7 Hz, 1H), 7.80 (s, 1H), 7.39 (dd, *J* = 7.7, 4.6 Hz, 1H), 7.22 (d, *J* = 7.9 Hz, 1H), 7.14 (d, *J* = 1.6 Hz, 1H), 7.07 (d, *J* = 8.0 Hz, 1H), 2.68 (s, 3H), 2.32 (s, 3H) and 2.17 (s, 3H). UPLC analysis (method C), 5.02 min, >98% purity.

6-Bromo-N-(4-methoxyphenyl)thieno[2,3-d]pyrimidin-4-amine 45

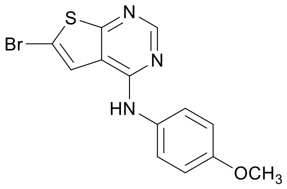

6-Bromo-4-chlorothieno[2,3-*d*]pyrimidine (500 mg, 2.00 mmol) and p-anisidine (247 mg, 2.00 mmol) were taken up in IPA (10mL) and heated at 120 °C for 1 hour in the microwave. The resulting solid was filtered and washed with EtOAc and DCM (Caution! Carcinogenic) to yield 6-bromo-*N*-(4-methoxyphenyl)thieno[2,3-*d*]pyrimidin-4-amine **45** (652 mg, 1.94 mmol, 97% yield). MS (ESI+) *m/z* calcd for $C_{13}H_{10}BrN_3OS^+$ $[M + H]^+$ 336.0 and 338.0, found 336.1 and 338.1. ^{1}H NMR (300 MHz, DMSO-*d*$_6$) δ 9.88 (s, 1H), 8.44 (s, 1H), 8.12 (s, 1H), 7.69 – 7.57 (m, 2H), 7.03 – 6.90 (m, 2H), 3.76 (s, 3H). UPLC analysis (method A), 3.07 min, >98% purity.

***N*-(4-methoxyphenyl)-6-(2-methyl-3-pyridyl)thieno[2,3-*d*]pyrimidin-4-amine (20)**

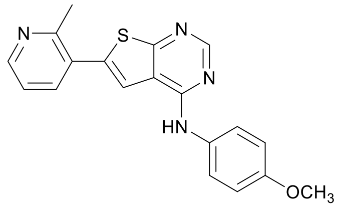

6-Bromo-*N*-(4-methoxyphenyl)thieno[2,3-*d*]pyrimidin-4-amine **45** (30.0 mg, 0.090 mmol) and (2-methylpyridin-3-yl)boronic acid (24.4 mg, 0.180 mmol) were reacted following general procedure 1 for 30 mins. Crude material was purified by preparative HPLC (elution gradient 5-95% of MeCN in H_2O with 0.1% NH_3) to yield *N*-(4-methoxyphenyl)-6-(2-methyl-3-pyridyl)thieno[2,3-*d*]pyrimidin-4-amine **20** (18.0 mg, 0.052 mmol, 58% yield). MS (ESI+) *m/z* calcd for $C_{19}H_{16}N_4OS^+$ $[M + H]^+$ 349.1, found 349.2. ^1H NMR (300 MHz, DMSO-d_6) δ 9.67 (s, 1H), 8.54 (dd, *J* = 4.8, 1.7 Hz, 1H), 8.45 (s, 1H), 7.98 (s, 1H), 7.90 (dd, *J* = 7.8, 1.7 Hz, 1H), 7.67 (d, *J* = 9.0 Hz, 2H), 7.40 (dd, *J* = 7.8, 4.8 Hz, 1H), 6.99 (d, *J* = 9.1 Hz, 2H), 3.77 (s, 3H) and 2.70 (s, 3H). UPLC analysis (method D), 3.68 min, >98% purity.

6-(2-methyl-3-pyridyl)-*N*-(m-tolyl)thieno[2,3-*d*]pyrimidin-4-amine (**21**)

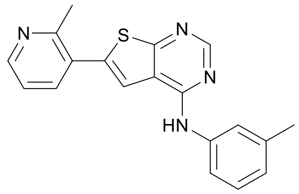

4-Chloro-6-(2-methyl-3-pyridyl)thieno[2,3-*d*]pyrimidine **44** (30.0 mg, 0.080 mmol) and 3-toluidine (0.02 mL, 0.160 mmol) were reacted following general procedure 3 at 140 °C to yield 6-(2-methyl-3-pyridyl)-*N*-(m-tolyl)thieno[2,3-*d*]pyrimidin-4-amine **21** (23.0 mg, 0.069 mmol, 86% yield). MS (ESI+) *m/z* calcd for $C_{19}H_{16}N_4S^+$ $[M + H]^+$ 333.1, found 333.2. ^1H NMR (300 MHz, DMSO-d_6) δ 9.66 (s, 1H), 8.54 (d, *J* = 5.8 Hz, 2H), 8.04 (s, 1H), 7.91 (d, *J* = 7.3 Hz, 1H), 7.66 (d, *J* = 12.0 Hz, 2H), 7.40 (dd, *J* = 7.8, 4.8 Hz, 1H), 7.29 (t, *J* = 7.7 Hz, 1H), 6.96 (d, *J* = 7.5 Hz, 1H), 2.71 (s, 3H), and 2.35 (s, 3H). UPLC analysis (method C), 5.18 min, >98% purity.

6-(2-methyl-3-pyridyl)-*N*-(p-tolyl)thieno[2,3-*d*]pyrimidin-4-amine (22)

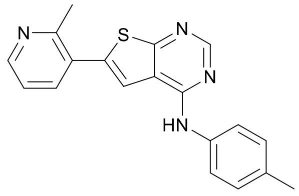

4-chloro-6-(2-methyl-3-pyridyl)thieno[2,3-*d*]pyrimidine **44** (30.0 mg, 0.080 mmol) and p-toluidine (17.2 mg, 0.160 mmol) were reacted following general procedure 3 at 140 °C to yield 6-(2-methyl-3-pyridyl)-*N*-(p-tolyl)thieno[2,3-*d*]pyrimidin-4-amine **22** (20.5 mg, 0.062 mmol, 77% yield). MS (ESI+) *m/z* calcd for $C_{19}H_{16}N_4S^+$ $[M + H]^+$ 333.1, found 333.2. ^1H NMR (300 MHz, DMSO-d_6) δ 9.68 (s, 1H), 8.51 (d, *J* = 12.8 Hz, 2H), 8.01 (s, 1H), 7.90 (d, *J* = 7.9 Hz, 1H), 7.69 (d, *J* = 7.9 Hz, 2H), 7.40 (s, 1H), 7.21 (d, *J* = 8.1 Hz, 2H), 2.71 (s, 3H) and 2.31 (s, 3H). UPLC analysis (method C), 5.18 min, >98% purity.

***N*-(4-ethylphenyl)-6-(2-methyl-3-pyridyl)thieno[2,3-*d*]pyrimidin-4-amine (23)**

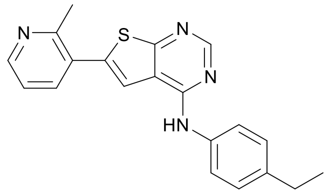

4-chloro-6-(2-methyl-3-pyridyl)thieno[2,3-*d*]pyrimidine **44** (30.0 mg, 0.080 mmol) and 4-ethylaniline (19.5 mg, 0.160 mmol) were reacted following general procedure 3 at 140 °C to yield *N*-(4-ethylphenyl)-6-(2-methyl-3-pyridyl)thieno[2,3-*d*]pyrimidin-4-amine **23** (20.0 mg, 0.058 mmol, 72% yield). MS (ESI+) *m/z* calcd for $C_{20}H_{18}N_4S^+$ $[M + H]^+$ 347.1, found 347.2. ^1H NMR (300 MHz, DMSO-d_6) δ 9.69 (s, 1H), 8.54 (dd, *J* = 4.8, 1.7 Hz, 1H), 8.49 (s, 1H), 8.01 (s, 1H), 7.91 (dd, *J* = 7.8, 1.7 Hz, 1H), 7.71 (d, *J* = 8.5 Hz, 2H), 7.40 (ddd, *J* = 7.8, 4.8, 0.7 Hz, 1H), 7.24 (d, *J* = 8.5 Hz, 2H), 2.71 (s, 3H), 2.61 (q, *J* = 7.6 Hz, 2H) and 1.20 (t, *J* = 7.6 Hz, 3H). UPLC analysis (method C), 5.59 min, >98% purity.

N-(4-cyclopropylphenyl)-6-(2-methyl-3-pyridyl)thieno[2,3-d]pyrimidin-4-amine (24)

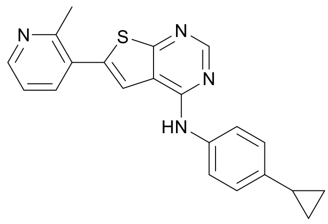

4-chloro-6-(2-methyl-3-pyridyl)thieno[2,3-*d*]pyrimidine **44** (30.0 mg, 0.080 mmol) and 4-cyclopropylaniline (21.4 mg, 0.160 mmol) were reacted following general procedure 3 at 140 °C to yield *N*-(4-cyclopropylphenyl)-6-(2-methyl-3-pyridyl)thieno[2,3-*d*]pyrimidin-4-amine **24** (24.0 mg, 0.067 mmol, 84% yield). MS (ESI+) *m/z* calcd for $C_{21}H_{18}N_4S^+$ $[M + H]^+$ 359.1, found 359.2. ^1H NMR (300 MHz, DMSO-d_6)δ 9.68 (s, 1H), 8.54 (dd, *J* = 4.8, 1.7 Hz, 1H), 8.49 (s, 1H), 8.00 (s, 1H), 7.90 (dd, *J* = 7.8, 1.7 Hz, 1H), 7.67 (d, *J* = 8.6 Hz, 2H), 7.40 (ddd, *J* = 7.8, 4.8, 0.6 Hz, 1H), 7.11 (d, *J* = 8.5 Hz, 2H), 2.70 (s, 3H), 1.92 (ddd, *J* = 13.4, 8.4, 5.0 Hz, 1H), 0.99 – 0.90 (m, 2H) and 0.71 – 0.62 (m, 2H). UPLC analysis (method C), 5.57 min, >98% purity.

6-bromo-N-(4-methylsulfanylphenyl)thieno[2,3-d]pyrimidin-4-amine (46)

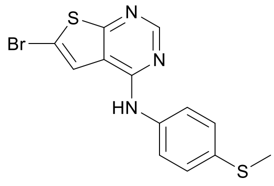

6-Bromo-4-chlorothieno[2,3-*d*]pyrimidine (500 mg, 2.00 mmol) and 4-(methylthio)aniline (279 mg, 2.00 mmol) were taken up in IPA (10 mL) and heated at 120 °C for 1 hour in the microwave. The resulting solid was filtered and washed with EtOAc and DCM (Caution! Carcinogenic) to yield 6-bromo-*N*-(4-methylsulfanylphenyl)thieno[2,3-*d*]pyrimidin-4-amine **46** (678 mg, 1.92 mmol, 96%). MS (ESI+) *m/z* calcd for $C_{12}H_7BrN_3S_2^+$ $[M + H]^+$ 352.0 and 354.0, found 352.0 and 354.0. ^1H NMR (300 MHz, DMSO-d_6) δ 9.79 (s, 1H), 8.48 (s, 1H), 8.14 (s, 1H), 7.85 – 7.73 (m, 2H), 7.37 – 7.24 (m, 2H), 2.48 (s, 3H). UPLC analysis (method A), 3.30 min, >98% purity.

6-(2-methyl-3-pyridyl)-*N*-(4-methylsulfanylphenyl)thieno[2,3-*d*]pyrimidin-4-amine (25)

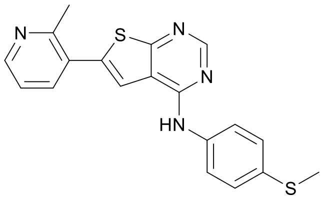

6-Bromo-*N*-(4-methylsulfanylphenyl)thieno[2,3-*d*]pyrimidin-4-amine **45** (39.0 mg, 0.110 mmol) and (2-methylpyridin-3-yl)boronic acid (30.3 mg, 0.220 mmol) were reacted following general procedure 1 for 30 mins. Crude material was purified by preparative HPLC (elution gradient 5-95% of MeCN in H_2O with 0.1% NH_3) to yield 6-(2-methyl-3-pyridyl)-*N*-(4-methylsulfanylphenyl)thieno[2,3-*d*]pyrimidin-4-amine **25** (2.7 mg, 0.007 mmol, 7% yield). MS (ESI+) *m/z* calcd for $C_{19}H_{16}N_4S_2^+$ $[M + H]^+$ 365.1, found 365.2. ^1H NMR (300 MHz, DMSO-d_6) δ 9.75 (s, 1H), 8.55 (dd, *J* = 4.9, 1.6 Hz, 1H), 8.52 (s, 1H), 8.02 (s, 1H), 7.91 (dd, *J* = 7.7, 1.7 Hz, 1H), 7.80 (d, *J* = 8.7 Hz, 2H), 7.40 (dd, *J* = 7.7, 4.8 Hz, 1H), 7.33 (d, *J* = 8.7 Hz, 2H), 2.71 (s, 3H), CH_3 obscured by residual solvent peak. UPLC analysis (method C), 4.68 min, 95% purity.

6-(3-methyl-4-pyridyl)-*N*-(4-methylsulfanylphenyl)thieno[2,3-*d*]pyrimidin-4-amine (**26**)

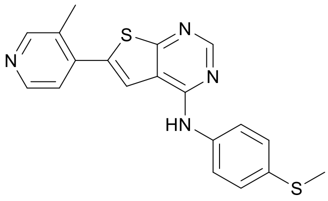

6-Bromo-*N*-(4-methylsulfanylphenyl)thieno[2,3-*d*]pyrimidin-4-amine **46** (30.0 mg, 0.085 mmol) and (3-methylpyridin-4-yl)boronic acid (23.3 mg, 0.170 mmol) were reacted following general procedure 1 for 30 mins. Crude material was purified by preparative HPLC (elution gradient 5-95% of MeCN in H_2O with 0.1% NH_3) to yield 6-(3-methyl-4-pyridyl)-*N*-(4-methylsulfanylphenyl)thieno[2,3-*d*]pyrimidin-4-amine **26** (15.4 mg,0.042 mmol, 50% yield). MS (ESI+) *m/z* calcd for $C_{19}H_{16}N_4S_2^+$ $[M + H]^+$ 365.1, found 365.2. ^1H NMR (300 MHz, DMSO-d_6) δ 9.82 (s, 1H), 8.61 (t, *J* = 0.7 Hz, 1H), 8.56 – 8.48 (m, 2H), 8.19 (s, 1H), 7.83 – 7.75 (m, 2H), 7.53 (d, *J* = 5.1 Hz, 1H), 7.38 – 7.26 (m, 2H),2.56 (s, 3H), CH_3 obscured by residual solvent peak. UPLC analysis (method C), 4.73 min, >98% purity.

N-(4-methoxyphenyl)-6-(3-methylpyridin-4-yl)thieno[2,3-d]pyrimidin-4-amine (27)

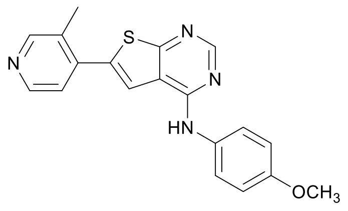

6-Bromo-*N*-(4-methoxyphenyl)thieno[2,3-*d*]pyrimidin-4-amine **45** (30.0 mg, 0.090 mmol) and (3-methylpyridin-4-yl)boronic acid (24.4 mg, 0.180 mmol) were reacted following general procedure 1 for 30 mins. Crude material was purified by preparative HPLC (elution gradient 5-95% of MeCN in H_2O with 0.1% NH_3) to yield *N*-(4-methoxyphenyl)-6-(3-methylpyridin-4-yl)thieno[2,3-*d*]pyrimidin-4-amine **27** (17.5 mg, 0.050 mmol, 56% yield). MS (ESI+) *m/z* calcd for $C_{19}H_{16}N_4OS^+$ $[M + H]^+$ 349.1, found 349.2. ^{1}H NMR (300 MHz, DMSO-d_6) 9.75 (s, 1H), 8.61 (s, 1H), 8.53 (d, J = 5.1 Hz, 1H), 8.47 (s, 1H), 8.15 (s, 1H), 7.66 (d, J = 8.7 Hz, 2H), 7.53 (d, J = 5.1 Hz, 1H), 6.99 (d, J = 9.0 Hz, 2H), 3.78 (s, 3H) and 2.56 (s, 3H). UPLC analysis (method D), 3.69 min, >98% purity.

6-(2-chloro-3-pyridyl)-N-(4-methylsulfanylphenyl)thieno[2,3-d]pyrimidin-4-amine (28)

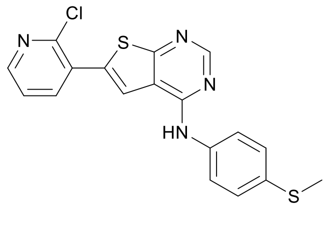

6-Bromo-*N*-(4-methylsulfanylphenyl)thieno[2,3-*d*]pyrimidin-4-amine **46** (30.0 mg, 0.090 mmol) and (2-chloropyridin-3-yl)boronic acid (26.8 mg, 0.170 mmol) were reacted following general procedure 1 for 30 mins. Crude material was purified by preparative HPLC (elution gradient 5-95% of MeCN in H_2O with 0.1% NH_3) to yield 6-(2-chloro-3-pyridyl)-*N*-(4-methylsulfanylphenyl)thieno[2,3-*d*]pyrimidin-4-amine **28** (16.5 mg, 0.043 mmol, 50% yield). MS (ESI+) *m/z* calcd for $C_{18}H_{13}ClN_4S_2^+$ $[M + H]^+$ 385.0, found 385.2. ^{1}H NMR (300 MHz, DMSO-d_6) δ 9.86 (s, 1H), 8.54 (s, 1H), 8.52 (dd, J = 4.7, 1.8 Hz, 1H), 8.30 (s, 1H), 8.20 (dd, J = 7.7, 1.9 Hz, 1H), 7.85 – 7.76 (m, 2H), 7.63 (dd, J = 7.7, 4.7 Hz, 1H), 7.36 – 7.28 (m, 2H), 2.49 (s, 3H). UPLC analysis (method C), 5.64 min, 97% purity.

6-isoxazol-4-yl-*N*-(4-methylsulfanylphenyl)thieno[2,3-*d*]pyrimidin-4-amine (29)

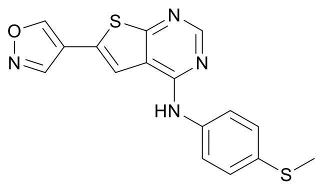

6-Bromo-*N*-(4-methylsulfanylphenyl)thieno[2,3-*d*]pyrimidin-4-amine **46** (30.0 mg, 0.090 mmol) and isoxazole-4-boronic acid (19.2 mg, 0.170 mmol) were reacted following general procedure 1 for 30 mins. Crude material was purified by preparative HPLC (elution gradient 5-95% of MeCN in H_2O with 0.1% NH_3) to yield 6-isoxazol-4-yl-*N*-(4-methylsulfanylphenyl)thieno[2,3-*d*]pyrimidin-4-amine **29** (5.0 mg, 0.015 mmol, 17% yield). MS (ESI+) *m/z* calcd for $C_{16}H_{12}N_4OS_2^+$ $[M + H]^+$ 341.0, found 341.1. ^1H NMR (300 MHz, DMSO-d_6) δ 9.27 (s, 1H), 8.69 (s, 1H), 8.25 (s, 1H), 7.88 (d, J = 8.8 Hz, 2H), 7.27 (d, J = 8.8 Hz, 2H), 7.21 (s, 1H) and 2.47 (s, 3H), exchangeable N*H* signal not observed. UPLC analysis (method C), 4.89 min, 94% purity.

6-(3,5-dimethylisoxazol-4-yl)-*N*-(4-methylsulfanylphenyl)thieno[2,3-*d*]pyrimidin-4-amine (30)

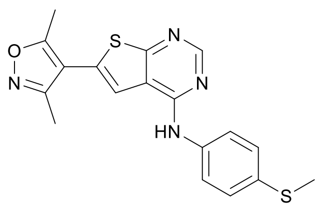

6-Bromo-*N*-(4-methylsulfanylphenyl)thieno[2,3-*d*]pyrimidin-4-amine **46** (30.0 mg, 0.090 mmol) (3,5-dimethyl-1,2-oxazol-4-yl)boronic acid (24.0 mg, 0.170 mmol) were reacted following general procedure 1 for 30 mins. Crude material was purified by preparative HPLC (elution gradient 5-95% of MeCN in H_2O with 0.1% NH_3) to yield 6-(3,5-dimethylisoxazol-4-yl)-*N*-(4-methylsulfanylphenyl)thieno[2,3-*d*]pyrimidin-4-amine **30** (2.0 mg, 0.005 mmol, 6% yield). MS (ESI+) *m/z* calcd for $C_{18}H_{16}N_4OS_2^+$ $[M + H]^+$ 369.1, found 369.2. ^1H NMR (300 MHz, DMSO-d_6) δ 9.73 (s, 1H), 8.50 (s, 1H), 7.92 (s, 1H), 7.77 (d, J = 8.7 Hz, 2H), 7.33 (d, J = 8.7 Hz, 2H), 2.62 (s, 3H), 2.49 (s, 3H), 2.43 (s, 3H). UPLC analysis (method C), 5.77 min, 94% purity.

5-methyl-*N*-(4-methylsulfonylphenyl)thieno[2,3-*d*]pyrimidin-4-amine (31)

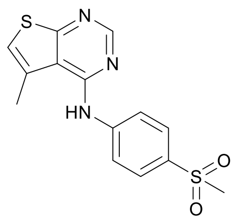

4-Chloro-5-methylthieno[2,3-*d*]pyrimidine (100 mg, 0.540 mmol) and 4-(methylsulfonyl)aniline hydrochloride (124 mg, 0.600 mmol) were suspended in 4N HCl in 1,4-dioxane (Caution! Carcinogenic; 3.0 mL) in a sealed tube and heated to 100 °C for 16 hours. The reaction was cooled and 7N methanolic ammonia added to basify the reaction before concentrating under reduced pressure. The crude material was purified by preparative HPLC (elution gradient 10-50% of MeCN in H_2O with 0.1% NH_3) to yield 5-methyl-*N*-(4-methylsulfonylphenyl)thieno[2,3-*d*]pyrimidin-4-amine **31** (46.8 mg, 0.147 mmol, 27% yield). MS (ESI+) *m/z* calcd for $C_{14}H_{13}N_3O_2S_2^+$ $[M + H]^+$ 320.0, found 320.1. ^1H NMR (300 MHz, Chloroform-*d*) δ 8.66 (s, 1H), 7.98 (s, 4H), 7.56 (s, 1H), 7.10 (q, J = 1.2 Hz, 1H), 3.09 (s, 3H), 2.86 – 2.79 (m, 3H). UPLC analysis (method C), 3.95 min, >98% purity.

5-Chloro-*N*-(4-(methylsulfonyl)phenyl)thieno[2,3-*d*]pyrimidin-4-amine (32)

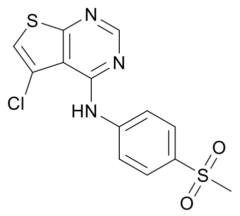

A 2-5 mL MW flask was charged with 4,5-dichlorothieno[2,3-*d*]pyrimidine (50.0 mg, 0.244 mmol) and 4-(methylsulfonyl)aniline (67.4 mg, 0.394 mmol), then dissolved in IPA (2.2 mL). The reaction mixture was heated under MW irradiation at 120 °C for 2 hrs. Upon completion the reaction was diluted with EtOAc (50 mL), washed with sat. aq. $NaHCO_3$ (2 × 50 mL) and brine (50 mL), then dried (hydrophobic frit) and concentrated in vacuo. Purification by preparatory HPLC (elution gradient 35-95% of MeCN in H_2O with 0.1% NH_3) yielded 5-chloro-*N*-(4-(methylsulfonyl)phenyl)thieno[2,3-*d*]pyrimidin-4-amine **32** (17.0 mg, 0.050 mmol, 21% yield) as a lyophilised white solid. MS (ESI+) *m/z* calcd. for $C_{13}H_{11}ClN_3O_2S_2^+$ $[M + H]^+$ 340.0; found 340.1. ^1H NMR (300

MHz, DMSO) δ 10.01 (s, 1H), 8.64 (s, 1H), 8.20 – 8.09 (m, 2H), 8.03 (s, 1H), 7.99 – 7.88 (m, 2H), 3.20 (s, 3H). UPLC analysis (method D), 4.69 min, >98%.

5-Bromo-*N*-(4-(methylsulfonyl)phenyl)thieno[2,3-*d*]pyrimidin-4-amine (47)

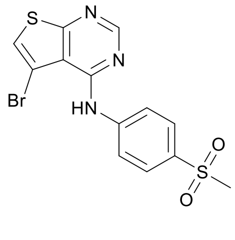

A 2-5 mL MW flask was charged with 5-bromo-4-chlorothieno[2,3-*d*]pyrimidine (172 mg, 0.689 mmol) and 4-(methylsulfonyl)aniline (130 mg, 0.758 mmol), then dissolved in IPA (4.6 mL) and the reaction mixture was heated under MW irradiation at 120 °C for 2 hrs. Upon completion the mixture was diluted with EtOAc (75 mL), washed with $NaHCO_3$ (2 × 50 mL) and brine (50 mL), dried (hydrophobic frit) and concentrated in vacuo. The crude material was triturated with MeOH (5 mL) to give 5-bromo-*N*-(4-(methylsulfonyl)phenyl)thieno[2,3-*d*]pyrimidin-4-amine **47** (221 mg, 0.575 mmol, 83% yield) as a yellow solid. MS (ESI+) *m/z* calcd. for $C_{13}H_{11}BrN_3O_2S_2^+$ $[M + H]^+$ 385.9; found 386.1. ^1H NMR (300 MHz, $CDCl_3$) δ 8.95 (s, 1H), 8.72 (s, 1H), 8.11 – 7.95 (m, 4H), 7.48 (s, 1H), 3.10 (s, 3H). UPLC analysis (method A), 2.81 min, >95%.

5-Cyclopropyl-*N*-(4-(methylsulfonyl)phenyl)thieno[2,3-*d*]pyrimidin-4-amine (33)

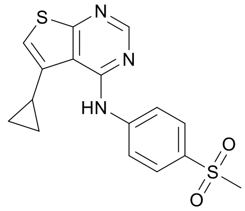

A 2-5 mL microwave flask was charged with 5-bromo-*N*-(4-(methylsulfonyl)phenyl)thieno[2,3-*d*]pyrimidin-4-amine **47** (20.0 mg, 0.052 mmol), cyclopropylboronic acid MIDA ester (15.4 mg, 0.078 mmol) and potassium carbonate (36.0 mg, 0.260 mmol). Toluene (0.4 mL) and water (0.1 mL) were added and the solution was deoxygenated by applying a vacuum and backfilling with N_2. Palladium (II) acetate (1.2 mg, 0.005 mmol) and {2-[2,6-bis(propan-2-yloxy)phenyl]phenyl}dicyclohexylphosphane (RuPhos) (4.9 mg, 0.010 mmol) were added,

the flask sealed under N_2 and the reaction mixture heated at 100 °C for 15 hrs. Upon completion the reaction mixture was diluted with DCM (Caution! Carcinogenic; 20 mL), passed through a hydrophobic frit, loaded onto an SCX-II column and washed with DCM (Caution! Carcinogenic) and MeOH. The product was eluted from the column with 0.5 M NH_3 in MeOH and the mixture was concentrated in vacuo. Purification by preparatory HPLC (elution gradient 20-95% of MeCN in H_2O with 0.1% NH_3) yielded 5-cyclopropyl-*N*-(4-methylsulfonylphenyl)thieno[2,3-*d*]pyrimidin-4-amine **33** (10.7 mg, 0.031 mmol, 60% yield) as a lyophilised white solid. MS (ESI+) *m/z* calcd. for $C_{16}H_{16}N_3O_2S_2^+$ [M + H]$^+$ 346.1; found 346.1. ^{1}H NMR (300 MHz, $CDCl_3$) δ 8.92 (s, 1H), 8.69 (s, 1H), 8.09 – 7.94 (m, 4H), 7.16 (d, *J* = 1.5 Hz, 1H), 3.10 (s, 3H), 2.35 – 2.20 (m, 1H), 1.33 – 1.22 (m, 2H), 1.13 – 1.02 (m, 2H). UPLC analysis (method D), 4.76 min, >98%.

N-Methyl-4-oxo-N-phenyl-3,4-dihydrothieno[2,3-d]pyrimidine-5-carboxamide (48)

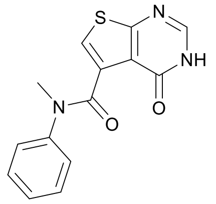

To a dry 2-5 mL MW flask containing 4-oxo-3,4-dihydrothieno[2,3-*d*]pyrimidine-5-carboxylic acid (100 mg, 0.510 mmol) in anhydrous DCM (Caution! Carcinogenic; 5.1 mL) was added *N*-methylaniline (0.066 mL, 0.612 mmol) and triethylamine (0.21 mL, 1.53 mmol). Propylphosphonic anhydride (50% w/w solution in EtOAc) (649 mg, 1.02 mmol) was added and the reaction mixture was stirred for 16 hrs. Upon completion the reaction was diluted with DCM (Caution! Carcinogenic; 25 mL), washed with sat. aq. NH_4Cl (20 mL) with water (20 mL) added, brine (25 mL), dried (hydrophobic frit) and concentrated in vacuo. Purification by silica gel chromatography (gradient elution 5 to 50% EtOAc in petroleum ether) followed by asiotroping with $CHCl_3$ (2 x 20 mL) yielded *N*-methyl-4-oxo-*N*-phenyl-3,4-dihydrothieno[2,3-*d*]pyrimidine-5-carboxamide **48** (86.0 mg, 0.301 mmol, 59% yield) as a white solid. MS (ESI+) *m/z* calcd. for $C_{14}H_{12}N_3O_2S^+$ [M + H]$^+$ 286.1; found 286.2. ^{1}H NMR (300 MHz, $CDCl_3$) δ 8.01 (s, 1H), 7.17 – 7.02 (m, 5H), 6.93 (s, 1H), 3.47 (s, 3H), exchangeable N*H* signal not observed. UPLC analysis (method A), 2.02 min, 97%.

4-Chloro-*N*-methyl-*N*-phenylthieno[2,3-*d*]pyrimidine-5-carboxamide (49)

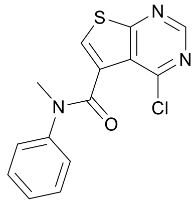

A 2-5 mL MW flask was charged with *N*-methyl-4-oxo-*N*-phenyl-3,4-dihydrothieno[2,3-*d*]pyrimidine-5-carboxamide **48** (85.0 mg, 0.298 mmol) and phosphorus oxychloride (Caution! Toxic; 0.28 mL, 2.98 mmol) and the reaction mixture heated at 100 °C for 1.5 hr. Upon completion the reaction was cooled, quenched by pouring onto ice water (20 mL) and extracted with DCM (Caution! Carcinogenic; 3 × 25 mL). The combined organic layers were dried (hydrophobic frit) and concentrated in vacuo to give 4-chloro-*N*-methyl-*N*-phenylthieno[2,3-*d*]pyrimidine-5-carboxamide **49** (82.0 mg, 0.270 mmol, 91% yield). LCMS indicated that some starting material was still present but with 80% purity the material was carried forward without further purification. MS (ESI+) *m/z* calcd. for $C_{14}H_{11}ClN_3OS^+$ $[M + H]^+$ 304.0; found 304.1. UPLC analysis (method A), 2.68 min, 80%.

***N*-Methyl-4-((4-(methylsulfonyl)phenyl)amino)-*N*-phenylthieno[2,3-*d*]pyrimidine-5-carboxamide (34)**

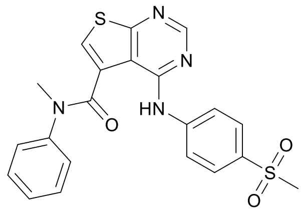

A dry 2-5 mL MW flask was charged with 4-chloro-*N*-methyl-*N*-phenyl-thieno[2,3-*d*]pyrimidine-5-carboxamide **49** (41.0 mg, 0.135 mmol), 4-(methylsulfonyl)aniline (24.2 mg, 0.142 mmol), 4,5-bis(diphenylphosphino)-9,9-dimethylxanthene (7.8 mg, 0.013 mmol) and cesium carbonate (132 mg, 0.405 mmol). Toluene (2.7 mL) was added and the reaction degassed by passing N_2 through for 5 min. Tris(dibenzylideneacetone)dipalladium(0) chloroform adduct (7.0 mg, 0.007 mmol) was added and the reaction mixture degassed again before heating under MW irradiation at 100 °C for 1.5 hrs. Upon completion the mixture was loaded onto an SCX-II column and washed with MeOH. The product was eluted with 0.5 M NH_3 in MeOH and concentrated in vacuo. LCMS indicated that product was present in both the MeOH wash and the NH_3 elution so both fractions were purified by preparatory HPLC (elution gradient 5-95% of MeCN in H_2O with 0.1% NH_3) yielding *N*-methyl-4-((4-

(methylsulfonyl)phenyl)amino)-*N*-phenylthieno[2,3-*d*]pyrimidine-5-carboxamide **34** (15.0 mg, 0.034 mmol, 25% yield) as an off white solid. MS (ESI+) *m/z* calcd. for $C_{21}H_{19}N_4O_3S_2^+$ [M + H]$^+$ 439.1; found 439.3. ^{1}H NMR (300 MHz, $CDCl_3$) δ 11.25 (s, 1H), 8.69 (s, 1H), 8.10 (d, *J* = 8.6 Hz, 2H), 7.99 (d, *J* = 8.7 Hz, 2H), 7.43 – 7.28 (m, 3H), 7.14 (d, *J* = 6.7 Hz, 2H), 6.99 (s, 1H), 3.64 (s, 3H), 3.10 (s, 3H). UPLC analysis (method D), 5.12 min, 96%.

5,6-dimethyl-*N*-(4-methylsulfonylphenyl)thieno[2,3-*d*]pyrimidin-4-amine (35)

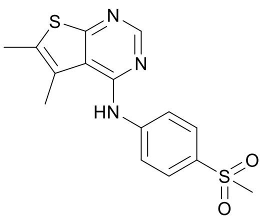

4-Chloro-5,6-dimethylthieno[2,3-*d*]pyrimidine (100 mg, 0.500 mmol) and 4-(methylsulfonyl)aniline hydrochloride (115 mg, 0.550 mmol) were suspended in 4N HCl in 1,4-dioxane (Caution! Carcinogenic; 3.0 mL) in a sealed tube and heated to 100 °C for 48 hours. The reaction was cooled and 7N methanolic ammonia added to basify the reaction before concentrating under reduced pressure. The crude material was purified by preparative HPLC (elution gradient 20-60% of MeCN in H_2O with 0.1% NH_3) to yield 5,6-dimethyl-*N*-(4-methylsulfonylphenyl)thieno[2,3-*d*]pyrimidin-4-amine **35** (32.9 mg, 0.147 mmol, 27% yield). MS (ESI+) *m/z* calcd for $C_{15}H_{15}N_3O_2S_2^+$ [M + H]$^+$ 334.0, found 334.1. ^{1}H NMR (300 MHz, Chloroform-*d*) δ 8.60 (s, 1H), 7.96 (s, 4H), 7.56 (s, 1H), 3.09 (s, 3H), 2.71 – 2.64 (m, 3H), 2.53 (d, *J* = 0.9 Hz, 3H). UPLC analysis (method C), 4.37 min, >98% purity.

***N*-(4-(Methylsulfonyl)phenyl)thieno[3,2-*d*]pyrimidin-4-amine (36)**

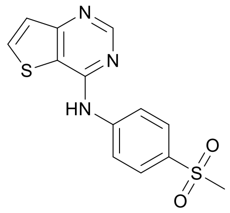

4-(Methylsulfonyl)aniline hydrochloride (50.0 mg, 0.240 mmol) was added to a suspension of 4-chlorothieno[3,2-*d*]pyrimidine (45.2 mg, 0.265 mmol) in 4M HCl in 1,4-dioxane (Caution! Carcinogenic; 4 mL) and the reaction was heated at 100 °C overnight. Upon cooling to rt, 0.5 M NH_3 was added and the reaction was concentrated in vacuo. Purification by preparatory HPLC (elution gradient 10-50% of MeCN in H_2O with 0.1% NH_3) yielded *N*-(4-(methylsulfonyl)phenyl)thieno[3,2-*d*]pyrimidin-4-amine **36** (36.2 mg, 0.119 mmol, 49% yield) as a white solid. MS (ESI+) *m/z* calcd. for $C_{13}H_{12}N_3O_2S_2^+$ $[M + H]^+$ 306.0; found 306.1. ^1H NMR (300 MHz, Chloroform-*d*) δ 8.87 (s, 1H), 8.07 – 7.94 (m, 4H), 7.89 (d, J = 5.4 Hz, 1H), 7.57 (d, J = 5.4 Hz, 1H), 6.99 (s, 1H), 3.10 (s, 3H). UPLC analysis (method C), 3.18 min, >98%.

6-Bromo-*N*-(4-(methylsulfonyl)phenyl)thieno[3,2-*d*]pyrimidin-4-amine (50)

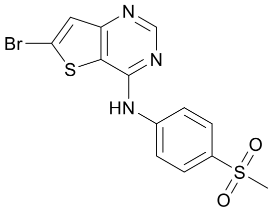

A 2-5 mL MW flask was charged with 6-bromo-4-chloro-thieno[3,2-*d*]pyrimidine (125 mg, 0.501 mmol) and 4-(methylsulfonyl)aniline (94.4 mg, 0.551 mmol), followed by IPA (5 mL). The reaction mixture was heated under MW irradiation at 120 °C for 2 hrs. Upon completion the reaction was diluted with EtOAc (50 mL), washed with sat. aq. $NaHCO_3$ (2 × 50 mL) and brine (50 mL), dried (hydrophobic frit) and concentrated in vacuo. Purification by trituration with MeOH (20 mL) gave 6-bromo-*N*-(4-(methylsulfonyl)phenyl)thieno[3,2-*d*]pyrimidin-4-amine **50** (144 mg, 0.375 mmol, 75% yield) as a white solid. MS (ESI+) *m/z* calcd. for $C_{13}H_{11}BrN_3O_2S_2^+$ $[M + H]^+$ 385.9; found 386.0. ^1H NMR (300 MHz, DMSO) δ 10.19 (s, 1H), 8.67 (s, 1H), 8.16 – 8.05 (m, 2H), 7.97 – 7.86 (m, 2H), 7.78 (s, 1H), 3.20 (s, 3H). UPLC analysis (method E), 2.66 min, >95%.

6-(2-Chloropyridin-3-yl)-*N*-(4-(methylsulfonyl)phenyl)thieno[3,2-*d*]pyrimidin-4-amine (37)

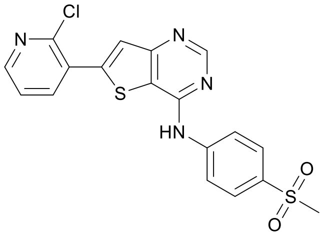

A 0.5-2 mL MW flask was charged with 6-bromo-*N*-(4-methylsulfonylphenyl)thieno[3,2}]pyrimidin-4-amine **50** (40.0 mg, 0.104 mmol), (2-chloropyridin-3-yl)boronic acid (24.6 mg, 0.156 mmol) and potassium carbonate (57.6 mg, 0.416 mmol) followed by 1,2-dimethoxyethane (0.8 mL) and water (0.2 mL). The reaction was degassed by bubbling N_2 through for 5 min, then dichloro[1,1'-bis(diphenylphosphino)ferrocene]palladium(II), complex with dichloromethane (1:1) (8.5 mg, 0.010 mmol) was added and the flask sealed under N_2. The reaction was heated at 120 °C under microwave irradiation for 30 min. Upon completion the reaction was diluted with DCM (Caution! Carcinogenic), passed through a hydrophobic frit onto an SCX-II column and washed with DCM (Caution! Carcinogenic) then MeOH. The column was eluted with 0.5 M NH_3 in MeOH and concentrated in vacuo. Purification by preparatory HPLC (elution gradient 10-52% of MeCN in H_2O with 0.1% NH_3) yielded 6-(2-chloropyridin-3-yl)-*N*-(4-(methylsulfonyl)phenyl)thieno[3,2-*d*]pyrimidin-4-amine **37** (14.0 mg, 0.034 mmol, 32% yield) as a white solid. MS (ESI+) *m/z* calcd. for $C_{18}H_{14}ClN_4O_2S_2^+$ $[M + H]^+$ 417.0; found 417.2. ^1H NMR (300 MHz, DMSO) δ 10.30 (s, 1H), 8.76 (s, 1H), 8.57 (dd, J = 4.7, 1.9 Hz, 1H), 8.33 (dd, J = 7.7, 1.9 Hz, 1H), 8.22 – 8.11 (m, 2H), 7.98 – 7.86 (m, 3H), 7.65 (dd, J = 7.7, 4.7 Hz, 1H), 3.21 (s, 3H). UPLC analysis (method F), 4.16 min, 96%.

4-Chloro-6-(2-methylpyridin-3-yl)thieno[3,2-*d*]pyrimidine (51)

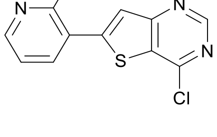

A 5 mL MW flask was charged with 6-bromo-4-chloro-thieno[3,2-*d*]pyrimidine (200 mg, 0.802 mmol) and (2-methylpyridin-3-yl)boronic acid (121 mg, 0.882 mmol), followed by 1,2-dimethoxyethane (4 mL) and water (0.75 mL). The reaction was degassed by bubbling N_2 through for 5 min before dichloro[1,1'-bis(diphenylphosphino)ferrocene]palladium(II), complex with dichloromethane (1:1) (66 mg, 0.080 mmol) was

added and the flask sealed under N_2. The reaction was heated at 120 °C under microwave irradiation for 30 min. Upon completion the reaction was diluted with EtOAc (80 mL) and IPA (15 mL), washed with sat. aq. $NaHCO_3$ (100 mL) and brine (100 mL). The aqueous layer was extracted with EtOAc (50 mL) and the combined organics were dried ($MgSO_4$), filtered and concentrated in vacuo to give 4-chloro-6-(2-methylpyridin-3-yl)thieno[3,2-*d*]pyrimidine **51** (134 mg, 0.512 mmol, 64% yield) as a pale brown solid of 88% purity which was used without further purification. MS (ESI+) *m/z* calcd. for $C_{12}H_9ClN_3S^+$ $[M + H]^+$ 262.0; found 262.1. ^1H NMR (300 MHz, $CDCl_3$) δ 9.05 (s, 1H), 8.68 (s, 1H), 7.87 (d, *J* = 7.2 Hz, 1H), 7.63 (s, 1H), 7.48 – 7.32 (m, 1H), 2.80 (s, 3H). UPLC analysis (method B), 2.39 min, 88%.

***N*-(4-Cyclopropylphenyl)-6-(2-methylpyridin-3-yl)thieno[3,2-*d*]pyrimidin-4-amine (38)**

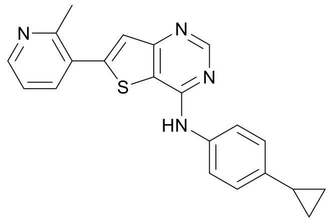

A 2-5 mL MW flask was charged with 4-cyclopropylaniline (20.8mg, 0.156 mmol) and a solution of 4-chloro-6-(2-methyl-3-pyridyl)thieno[3,2-*d*]pyrimidine **51** (34.0 mg, 0.130 mmol) in IPA (2 mL) and DMSO (0.2 mL) was added. The reaction mixture was heated under MW irradiation at 120 °C for 2 hrs. Upon completion the reaction was concentrated in vacuo to give a crude material that still contained DMSO. Purification by preparatory HPLC (elution gradient 20-70% of MeCN in H_2O with 0.1% NH_3) yielded *N*-(4-cyclopropylphenyl)-6-(2-methylpyridin-3-yl)thieno[3,2-*d*]pyrimidin-4-amine **38** (24.4 mg, 0.068 mmol, 52% yield) as a pale brown solid. MS (ESI+) *m/z* calcd. for $C_{21}H_{19}N_4S^+$ $[M + H]^+$ 359.1; found 359.2. ^1H NMR (300 MHz, MeOD) δ 8.47 – 8.37 (m, 2H), 7.87 – 7.78 (m, 1H), 7.43 (d, *J* = 8.2 Hz, 2H), 7.38 – 7.26 (m, 2H), 7.03 (d, *J* = 8.4 Hz, 2H), 2.57 (s, 3H), 1.84 (td, *J* = 8.5, 4.3 Hz, 1H), 0.94 – 0.82 (m, 2H), 0.60 (dt, *J* = 6.7, 4.6 Hz, 2H), exchangeable N*H* signal not observed. UPLC analysis (method F), 5.12 min, >98%.

6-Bromo-*N*-(4-(methylthio)phenyl)thieno[3,2-*d*]pyrimidin-4-amine (52)

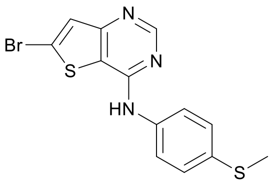

A 2-5 mL MW flask was charged with 6-bromo-4-chloro-thieno[3,2-*d*]pyrimidine (125 mg, 0.501 mmol) and 4-(methylthio)aniline (0.074 mL, 0.60 mmol) and IPA (5 mL). The reaction mixture was heated under MW irradiation at 120 °C for 2 hrs. Upon completion the reaction was diluted with EtOAc (50 mL), washed with sat. aq. $NaHCO_3$ (2 × 50 mL) and brine (50 mL), dried (hydrophobic frit) and concentrated in vacuo. Purification by trituration with MeOH (5 mL) gave 6-bromo-*N*-(4-(methylthio)phenyl)thieno[3,2-*d*]pyrimidin-4-amine **52** (145 mg, 0.412 mmol, 82% yield) as a white solid. MS (ESI+) *m/z* calcd. for $C_{13}H_{11}BrN_3S_2^+$ $[M + H]^+$ 354.0; found 354.0. ^1H NMR (300 MHz, DMSO) δ 9.76 (s, 1H), 8.53 (s, 1H), 7.74 – 7.63 (m, 3H), 7.35 – 7.25 (m, 2H), 2.49 (s, 3H). UPLC analysis (method E), 3.09 min, 92%.

6-(2-Chloropyridin-3-yl)-*N*-(4-(methylthio)phenyl)thieno[3,2-*d*]pyrimidin-4-amine (39)

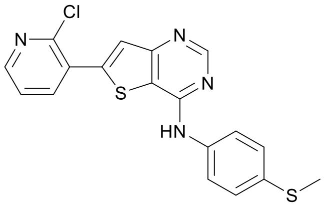

A 2-5 mL MW flask was charged with 6-bromo-*N*-(4-methylsulfanylphenyl)thieno[3,2-*d*]pyrimidin-4-amine **52** (45.0 mg, 0.128 mmol) and (2-chloropyridin-3-yl)boronic acid (40.2 mg, 0.255 mmol) and 1,2-dimethoxyethane (2 mL) and water (0.5 mL) were added. The reaction was degassed by bubbling N_2 through for 5 min before dichloro[1,1'-bis(diphenylphosphino)ferrocene]palladium(II), complex with dichloromethane (1:1) (10.5 mg, 0.013 mmol) was added and the flask sealed under N_2. The reaction was heated at 120 °C under microwave irradiation for 30 min. Upon completion the reaction was diluted with DCM (Caution! Carcinogenic), passed through a hydrophobic frit onto an SCX-II column and washed with DCM (Caution! Carcinogenic) and MeOH. The column was eluted with 0.5 M NH_3 in MeOH then concentrated in vacuo. Purification by preparatory HPLC (elution gradient 25-70% of MeCN in H_2O with 0.1% NH_3) yielded 6-(2-chloropyridin-3-yl)-*N*-(4-(methylthio)phenyl)thieno[3,2-*d*]pyrimidin-4-amine **39** (7.0 mg, 0.018 mmol, 14% yield) as a pale yellow solid.

MS (ESI+) *m/z* calcd. for $C_{18}H_{14}ClN_4S_2^+$ $[M + H]^+$ 385.0; found 385.1. ^{1}H NMR (300 MHz, MeOD) δ 8.58 (s, 1H), 8.50 (dd, *J* = 4.8, 1.9 Hz, 1H), 8.18 (dd, *J* = 7.7, 1.9 Hz, 1H), 7.75 – 7.64 (m, 3H), 7.57 (dd, *J* = 7.7, 4.8 Hz, 1H), 7.40 – 7.30 (m, 2H), 2.52 (s, 3H), exchangeable N*H* signal not observed. ^{13}C NMR (126 MHz, DMSO) δ 160.2, 155.2, 155.0, 150.7, 148.3, 144.3, 141.3, 136.8, 133.0, 129.1, 127.3, 126.5, 124.2, 123.3, 117.0, 16.0. UPLC analysis (method F), 5.15 min, 97%.

6-(2-Methylpyridin-3-yl)-*N*-(4-(pentafluoro-l6-sulfaneyl)phenyl)thieno[3,2-*d*]pyrimidin-4-amine (40)

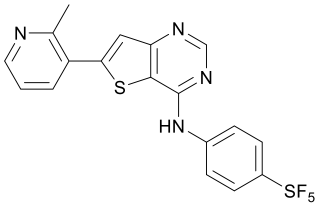

A 0.5-2 mL MW flask was charged with 4-aminophenylsulphur pentafluoride (25.1 mg, 0.115 mmol) and a solution of 4-chloro-6-(2-methyl-3-pyridyl)thieno[3,2-*d*]pyrimidine **51** (25.0 mg, 0.096 mmol) in IPA (1.9 mL) was added. The reaction mixture was heated under MW irradiation at 120 °C for 2 hrs. Upon completion the reaction was concentrated in vacuo. Purification by preparatory HPLC (elution gradient 30-75% of MeCN in H_2O with 0.1% NH_3) yielded 6-(2-methylpyridin-3-yl)-*N*-(4-(pentafluoro-l6-sulfaneyl)phenyl)thieno[3,2-*d*]pyrimidin-4-amine **40** (17.0 mg, 0.038 mmol, 40% yield) as a white lyophilised solid. MS (ESI+) *m/z* calcd. for $C_{18}H_{14}F_5N_4S_2^+$ $[M + H]^+$ 445.1; found 445.2. ^{1}H NMR (300 MHz, MeOD) δ 8.71 (s, 1H), 8.56 (dd, *J* = 5.0, 1.7 Hz, 1H), 8.14 – 8.05 (m, 2H), 8.00 (dd, *J* = 7.8, 1.7 Hz, 1H), 7.89 – 7.78 (m, 2H), 7.55 (s, 1H), 7.45 (ddd, *J* = 7.8, 5.0, 0.7 Hz, 1H), 2.72 (s, 3H), exchangeable N*H* signal not observed. ^{19}F NMR (282 MHz, MeOD) δ 85.70 – 82.87 (m, 1F), 62.17 (d, *J* = 147.8 Hz, 4F). UPLC analysis (method F), 5.76 min, >98%.

***N*-(6-(2-Methylpyridin-3-yl)thieno[3,2-*d*]pyrimidin-4-yl)benzo[d]oxazol-6-amine (41)**

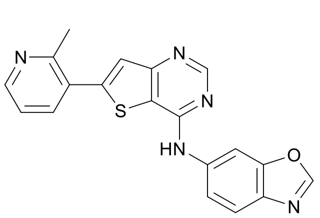

A 2-5 mL MW flask was charged with 6-aminobenzoxazole (20.9 mg, 0.156 mmol) and a solution of 4-chloro-6-(2-methyl-3-pyridyl)thieno[3,2-*d*]pyrimidine **51** (34.0 mg, 0.130 mmol) in IPA (2 mL) and DMSO (0.2 mL) was

added. The reaction mixture was heated under MW irradiation at 120 °C for 2 hrs. Upon completion the reaction was concentrated in vacuo to give a crude mixture that contained DMSO. Purification by preparatory HPLC (elution gradient 5-95% of MeCN in H_2O with 0.1% NH_3) yielded *N*-(6-(2-methylpyridin-3-yl)thieno[3,2-*d*]pyrimidin-4-yl)benzo[d]oxazol-6-amine **41** (1.5 mg, 0.004 mmol, 3% yield) as a brown solid. MS (ESI+) *m/z* calcd. for $C_{19}H_{14}N_5OS^+$ $[M + H]^+$ 360.1; found 360.2. ^1H NMR (300 MHz, MeOD) δ 8.55 (s, 1H), 8.44 (d, *J* = 4.8 Hz, 1H), 8.38 (s, 1H), 8.30 (s, 1H), 7.88 (d, *J* = 7.6 Hz, 1H), 7.65 (d, *J* = 7.9 Hz, 1H), 7.61 – 7.54 (m, 1H), 7.42 (s, 1H), 7.38 – 7.29 (m, 1H), 2.60 (s, 3H), exchangeable N*H* signal not observed. UPLC analysis (method F), 3.89 min, 94%.

1-(4-((6-(2-Methylpyridin-3-yl)thieno[3,2-*d*]pyrimidin-4-yl)amino)phenyl)pyrrolidin-2-one 42

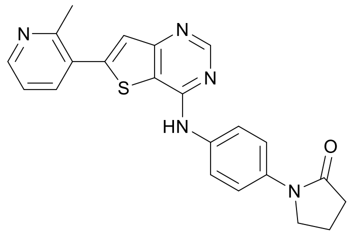

A 2-5 mL MW flask was charged with 1-(4-aminophenyl)-2-pyrrolidinone (27.5 mg, 0.156 mmol) and a solution of 4-chloro-6-(2-methyl-3-pyridyl)thieno[3,2-*d*]pyrimidine **51** (34.0 mg, 0.130 mmol) in IPA (2 mL) was added. The reaction mixture was heated under MW irradiation at 120 °C for 2 hrs. Upon completion the reaction was concentrated in vacuo. Purification by preparatory HPLC (elution gradient 10-50% of MeCN in H_2O with 0.1% NH_3) yielded 1-(4-((6-(2-methylpyridin-3-yl)thieno[3,2-*d*]pyrimidin-4-yl)amino)phenyl)pyrrolidin-2-one **42** (33.3 mg, 0.083 mmol, 64% yield) as a pale yellow solid. MS (ESI+) *m/z* calcd. for $C_{22}H_{20}N_5OS^+$ $[M + H]^+$ 402.1; found 402.3. ^1H NMR (300 MHz, MeOD) δ 8.47 (s, 1H), 8.43 (dd, *J* = 5.0, 1.7 Hz, 1H), 7.85 (dd, *J* = 7.8, 1.7 Hz, 1H), 7.69 – 7.61 (m, 2H), 7.59 – 7.51 (m, 2H), 7.38 (s, 1H), 7.32 (ddd, *J* = 7.8, 5.0, 0.7 Hz, 1H), 3.92 – 3.81 (m, 2H), 2.59 (s, 3H), 2.55 – 2.48 (m, 2H), 2.18 – 2.02 (m, 2H), exchangeable N*H* signal not observed. UPLC analysis (method F), 3.90 min, >98%.

Spectra for key compounds 8, 10, 14, 15, 17, 26, 32, 37, 38, 39 and 42

6-(1-methylpyrazol-4-yl)-*N*-(4-methylsulfonylphenyl)thieno[2,3-*d*]pyrimidin-4-amine **8**

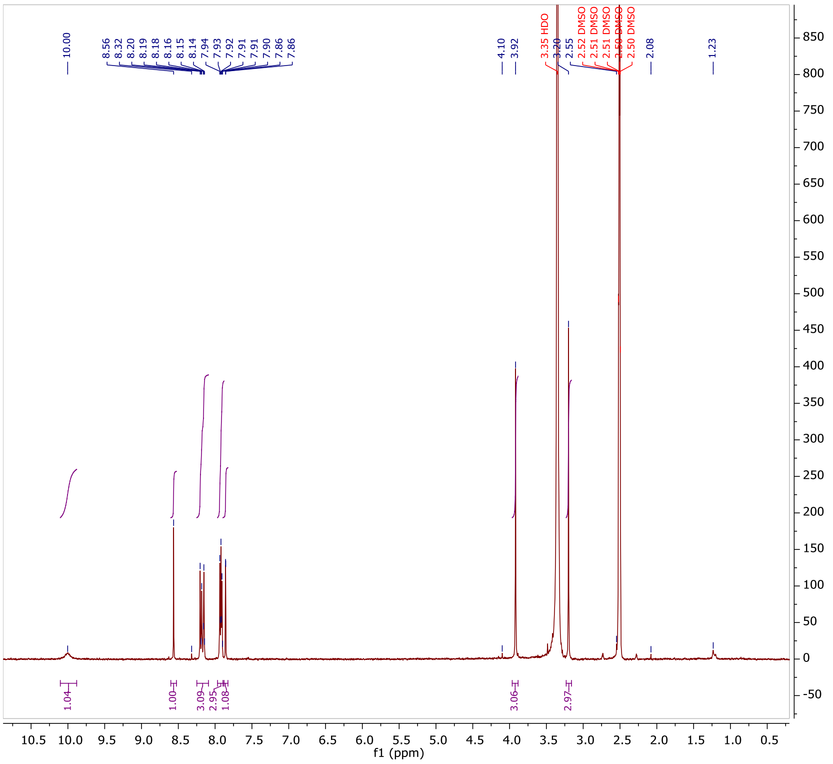

Parameter	Value
Title	GGA2-23-2.10.fid
Origin	Bruker BioSpin GmbH
Instrument	spect
Solvent	DMSO
Temperature	297.6
Pulse Sequence	zg30
Experiment	1D
Probe	5 mm PABBO BB-1H/ D Z-GRD Z104275/ 0242
Number of Scans	16
Receiver Gain	191.9
Relaxation Delay	1.0000
Pulse Width	14.0000
Spectrometer Frequency	300.13
Spectral Width	6009.6
Lowest Frequency	-1151.4
Nucleus	1H
Acquired Size	32768
Spectral Size	65536

1H NMR (300 MHz, DMSO-d_6) δ 10.00 (s, 1H), 8.56 (s, 1H), 8.25 – 8.09 (m, 3H), 7.97 – 7.88 (m, 3H), 7.86 (d, J = 0.8 Hz, 1H), 3.92 (s, 3H), 3.20 (s, 3H).

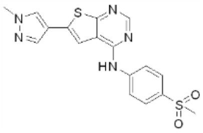

6-(3-methyl-4-pyridyl)-*N*-(4-methylsulfonylphenyl)thieno[2,3-*d*]pyrimidin-4-amine **10**

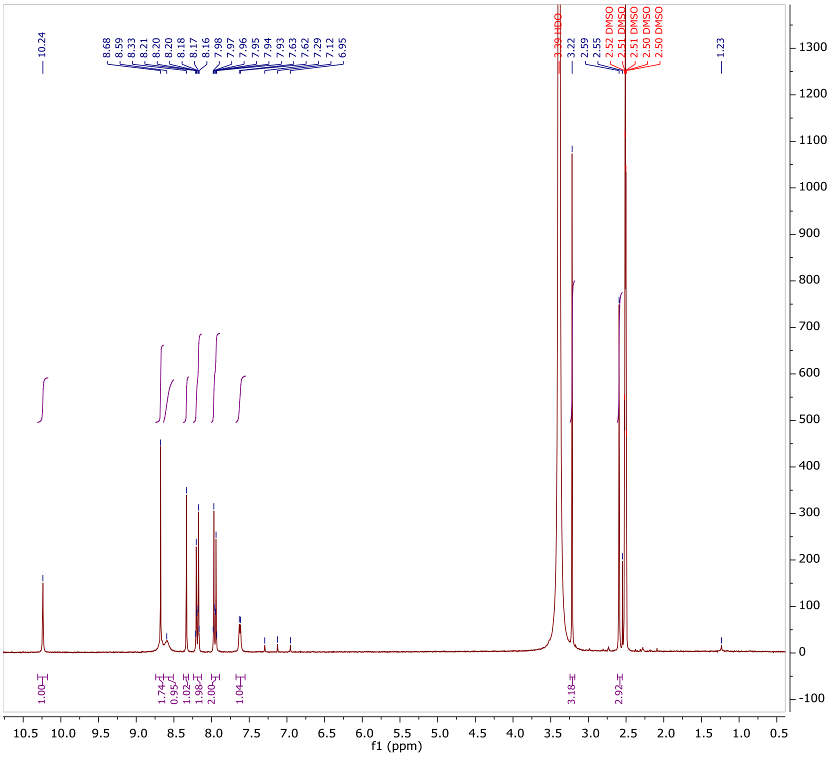

Parameter	Value
Title	GGA2-40-2-conc. 10.fid
Origin	Bruker BioSpin GmbH
Instrument	spect
Solvent	DMSO
Temperature	295.1
Pulse Sequence	zg30
Experiment	1D
Probe	5 mm PABBO BB-1H/ D Z-GRD Z104275/ 0242
Number of Scans	16
Receiver Gain	191.9
Relaxation Delay	1.0000
Pulse Width	14.0000
Spectrometer Frequency	300.13
Spectral Width	6009.6
Lowest Frequency	-1151.4
Nucleus	1H
Acquired Size	32768
Spectral Size	65536

1H NMR (300 MHz, DMSO-d_6) δ 10.24 (s, 1H), 8.68 (s, 2H), 8.59 (s, 1H), 8.33 (s, 1H), 8.19 (d, J = 8.9 Hz, 2H), 7.96 (d, J = 8.9 Hz, 2H), 7.62 (d, J = 5.0 Hz, 1H), 3.22 (s, 3H), 2.5 (s, 3H).

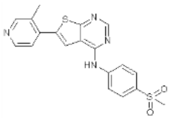

6-(4-methyl-3-pyridyl)-*N*-(4-methylsulfonylphenyl)thieno[2,3-*d*]pyrimidin-4-amine **14**

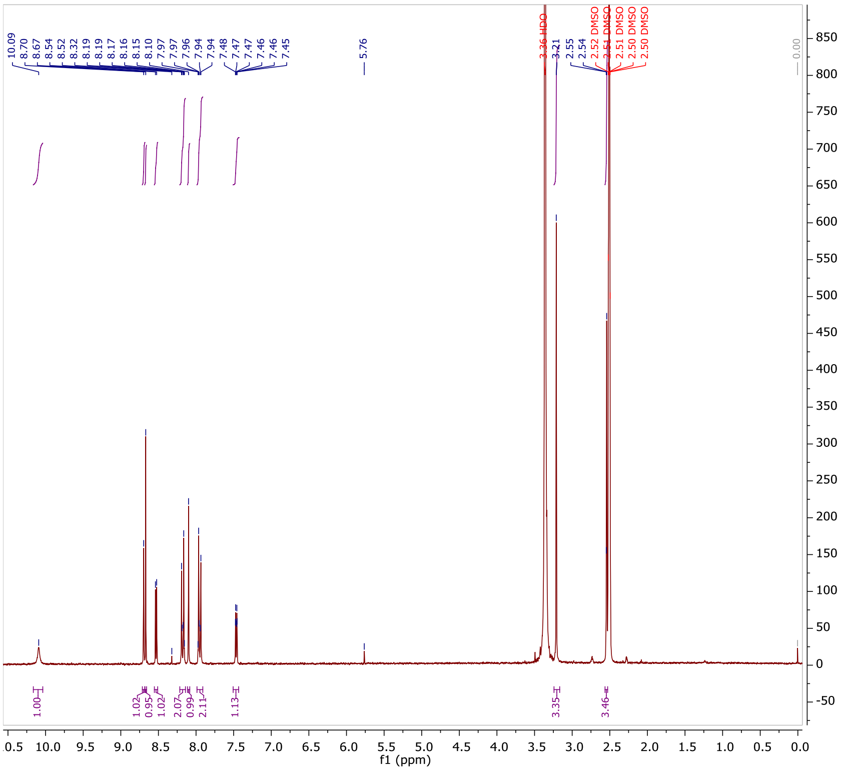

Parameter	Value
Title	GGA2-72-2.10.fid
Origin	Bruker BioSpin GmbH
Instrument	spect
Solvent	DMSO
Temperature	295.0
Pulse Sequence	zg30
Experiment	1D
Probe	5 mm PABBO BB-1H/ D Z-GRD Z104275/ 0242
Number of Scans	16
Receiver Gain	191.9
Relaxation Delay	1.0000
Pulse Width	14.0000
Spectrometer Frequency	300.13
Spectral Width	6009.6
Lowest Frequency	-1151.4
Nucleus	1H
Acquired Size	32768
Spectral Size	65536

1H NMR (300 MHz, DMSO-d_6) δ 10.09 (s, 1H), 8.70 (s, 1H), 8.67 (s, 1H), 8.53 ([illegible] *J* = 5.0 Hz, 1H), 8.22 – 8.14 (m, 2H), 8.1[illegible] (s, 1H), 7.99 – 7.91 (m, 2H), 7.47 (dt, *J* = 5.0, 0.7 Hz, 1H), 3.21 (s, 3H), 2.54 (d, *J* = 1.7 Hz, 3H).

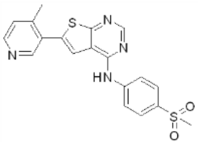

6-(2-chloro-3-pyridyl)-*N*-(4-methylsulfonylphenyl)thieno[2,3-*d*]pyrimidin-4-amine **15**

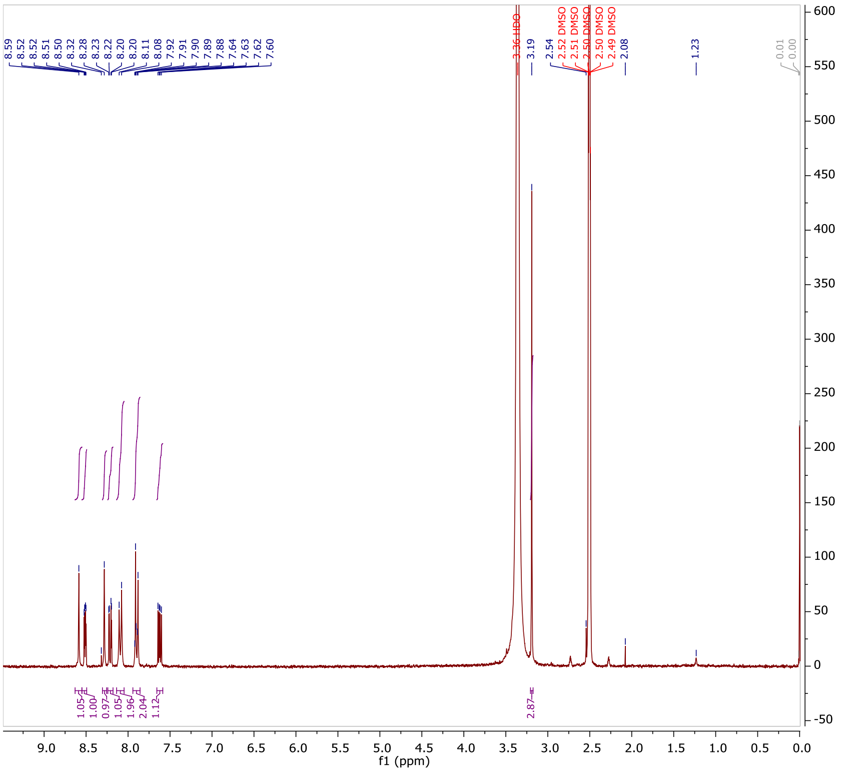

Parameter	Value
Title	GGA2-47-2.10.fid
Origin	Bruker BioSpin GmbH
Instrument	spect
Solvent	DMSO
Temperature	294.9
Pulse Sequence	zg30
Experiment	1D
Probe	5 mm PABBO BB-1H/ D Z-GRD Z104275/ 0242
Number of Scans	16
Receiver Gain	191.9
Relaxation Delay	1.0000
Pulse Width	14.0000
Spectrometer Frequency	300.13
Spectral Width	6009.6
Lowest Frequency	-1152.4
Nucleus	1H
Acquired Size	32768
Spectral Size	65536

1H NMR (300 MHz, DMSO-d_6) δ 8.59 (s, 1H), 8.51 (dd, *J* = 4.7, 1.9 Hz, 1H), 8.28 (s, 1H), 8.21 (dd, *J* = 7.7, 1.9 Hz, 1H), 8.09 (d, *J* = 8.7 Hz, 2H), 7.94 – 7.86 (m, 2H), 7.62 (dd, *J* = 7.7, 4.7 Hz, 1H), 3.19 (s, 3H).

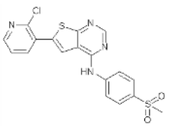

6-(2-methyl-3-pyridyl)-*N*-(4-methylsulfonylphenyl)thieno[2,3-*d*]pyrimidin-4-amine **17**

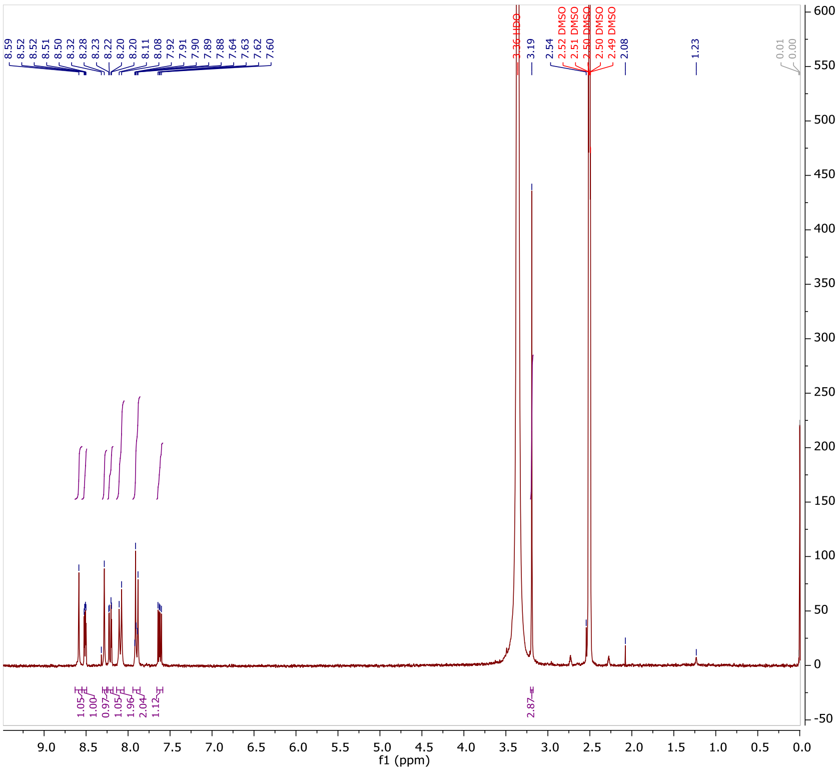

Parameter	Value
Title	GGA2-47-2.10.fid
Origin	Bruker BioSpin GmbH
Instrument	spect
Solvent	DMSO
Temperature	294.9
Pulse Sequence	zg30
Experiment	1D
Probe	5 mm PABBO BB-1H/ D Z-GRD Z104275/ 0242
Number of Scans	16
Receiver Gain	191.9
Relaxation Delay	1.0000
Pulse Width	14.0000
Spectrometer Frequency	300.13
Spectral Width	6009.6
Lowest Frequency	-1152.4
Nucleus	1H
Acquired Size	32768
Spectral Size	65536

1H NMR (300 MHz, DMSO-d_6) δ 8.59 (s, 1H), 8.51 (dd, *J* = 4.7, 1.9 Hz, 1H), 8.28 (s, 1H), 8.21 (dd, *J* = 7.7, 1.9 Hz, 1H), 8.09 (d, *J* = 8.7 Hz, 2H), 7.94 – 7.86 (m, 2H), 7.62 (dd, *J* = 7.7, 4.7 Hz, 1H), 3.19 (s, 3H).

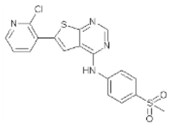

6-(3-methyl-4-pyridyl)-*N*-(4-methylsulfanylphenyl)thieno[2,3-*d*]pyrimidin-4-amine **26**

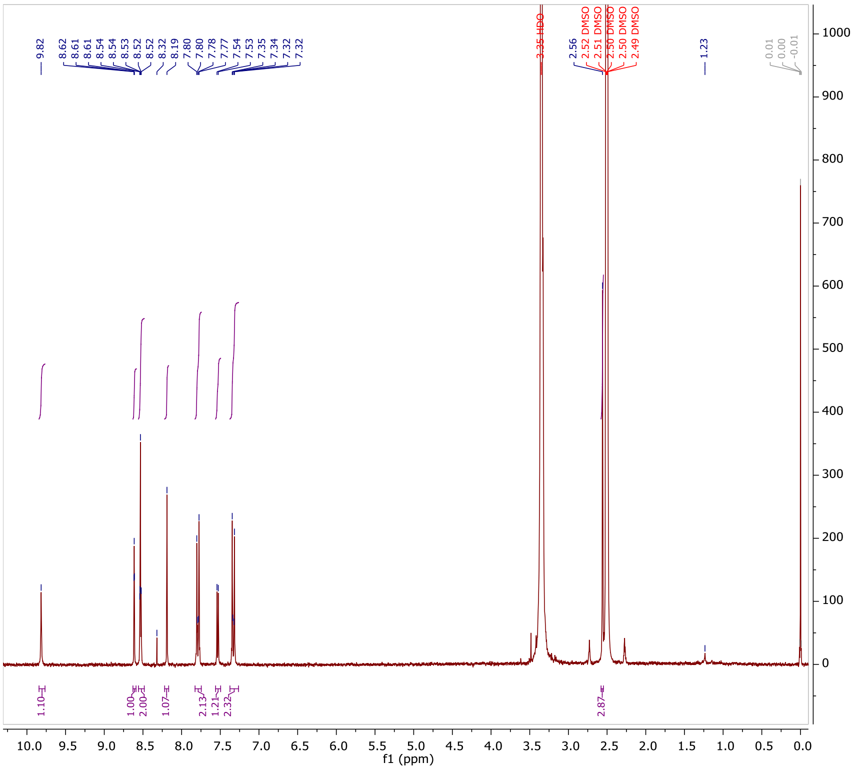

Parameter	Value
Title	GGA2-61-2.10.fid
Origin	Bruker BioSpin GmbH
Instrument	spect
Solvent	DMSO
Temperature	294.6
Pulse Sequence	zg30
Experiment	1D
Probe	5 mm PABBO BB-1H/ D Z-GRD Z104275/ 0242
Number of Scans	64
Receiver Gain	191.9
Relaxation Delay	1.0000
Pulse Width	14.0000
Spectrometer Frequency	300.13
Spectral Width	6009.6
Lowest Frequency	-1152.7
Nucleus	1H
Acquired Size	32768
Spectral Size	65536

1H NMR (300 MHz, DMSO-d_6) δ 9.82 (s, 1H), 8.61 (t, *J* = 0.7 Hz, 1H), 8.56 – 8.48 (m, 2H), 8.19 (s, 1H), 7.83 – 7.75 (m, 2H 7.53 (d, *J* = 5.1 Hz, 1H), 7.38 – 7.26 (m, 2H), 2.56 (s, 3H).

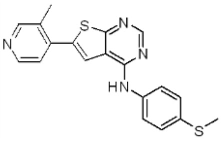

5-Chloro-*N*-(4-(methylsulfonyl)phenyl)thieno[2,3-*d*]pyrimidin-4-amine **32**

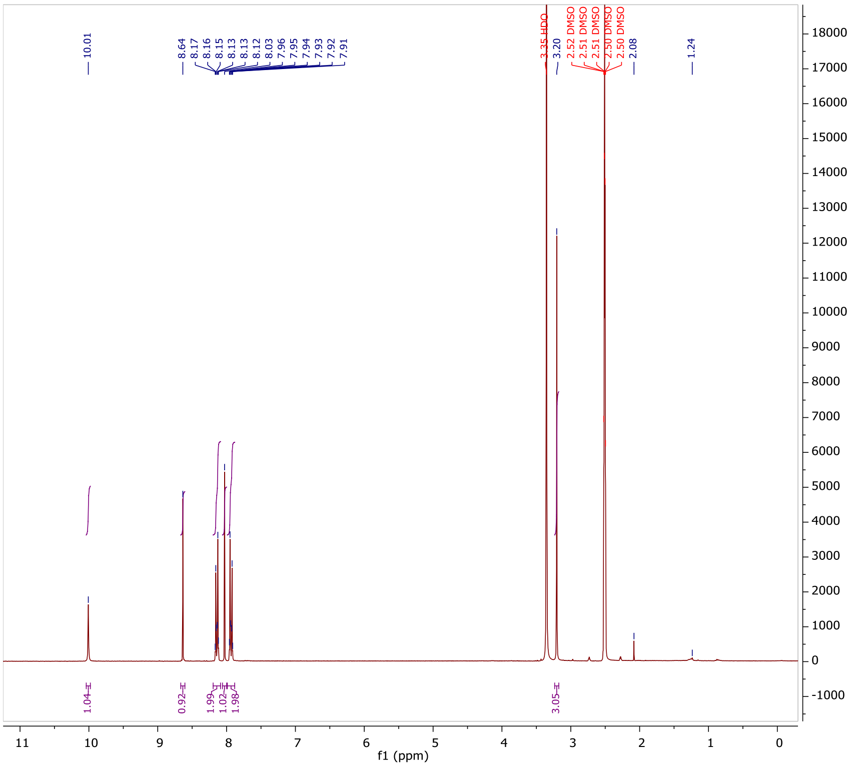

Parameter	Value
Title	tpcr5-075-3.10.fid
Origin	Bruker BioSpin GmbH
Instrument	spect
Solvent	DMSO
Temperature	294.5
Pulse Sequence	zg30
Experiment	1D
Probe	5 mm PABBO BB-1H/ D Z-GRD Z104275/ 0242
Number of Scans	128
Receiver Gain	191.9
Relaxation Delay	1.0000
Pulse Width	14.0000
Spectrometer Frequency	300.13
Spectral Width	6009.6
Lowest Frequency	-1151.4
Nucleus	1H
Acquired Size	32768
Spectral Size	65536

1H NMR (300 MHz, DMSO-d_6) δ 10.01 (s, 1H), 8.64 (s, 1H), 8.20 – 8.09 (m, 2H), 8.03 (s, 1H), 7.99 – 7.88 (m, 2H), 3.20 (s, 3H).

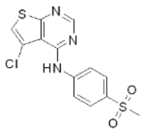

6-(2-Chloropyridin-3-yl)-*N*-(4-(methylsulfonyl)phenyl)thieno[3,2-*d*]pyrimidin-4-amine **37**

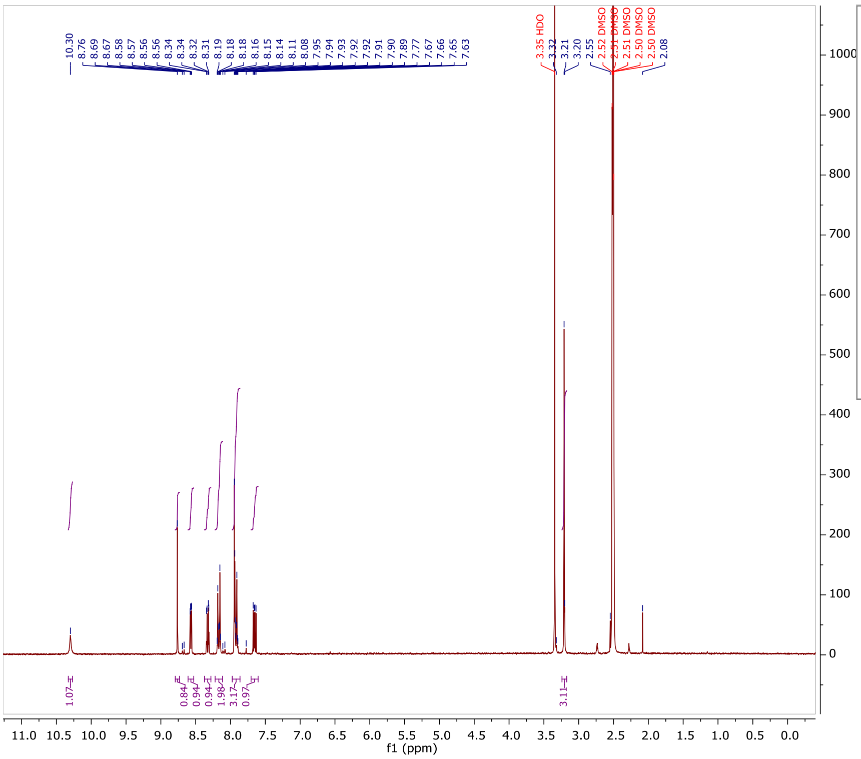

Parameter	Value
Title	tpcr5-092-3 rep. 10.fid
Origin	Bruker BioSpin GmbH
Instrument	spect
Solvent	DMSO
Temperature	293.8
Pulse Sequence	zg30
Experiment	1D
Probe	5 mm PABBO BB-1H/ D Z-GRD Z104275/ 0242
Number of Scans	16
Receiver Gain	191.9
Relaxation Delay	1.0000
Pulse Width	14.0000
Spectrometer Frequency	300.13
Spectral Width	6009.6
Lowest Frequency	-1151.4
Nucleus	1H
Acquired Size	32768
Spectral Size	65536

1H NMR (300 MHz, DMSO-d_6) δ 10.30 (s, 1H), 8.76 (s, 1H), 8.57 (dd, *J* = 4.7, 1.9 Hz, 1H), 8.33 (dd, *J* = 7.7, 1.9 Hz, 1H), 8.22 – 8.11 (m, 2H), 7.98 – 7.86 (m, 3H), 7.65 (dd, *J* = 7.7, 4.7 Hz, 1H), 3.21 (s, 3H).

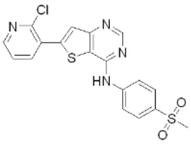

N-(4-Cyclopropylphenyl)-6-(2-methylpyridin-3-yl)thieno[3,2-*d*]pyrimidin-4-amine **38**

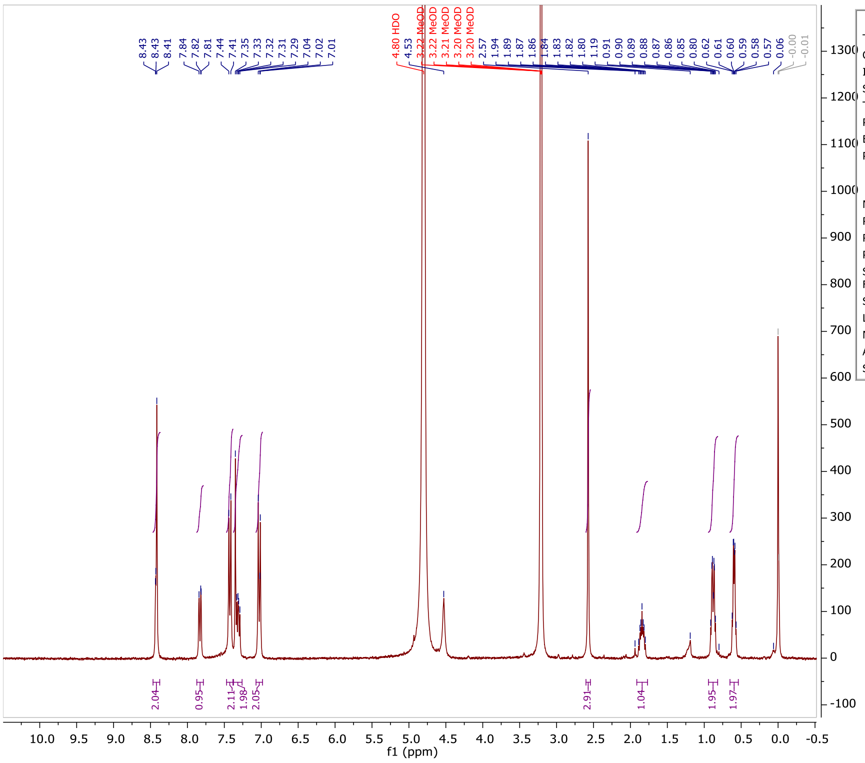

Parameter	Value
Title	tpcr6-009-3.10.fid
Origin	Bruker BioSpin GmbH
Instrument	spect
Solvent	MeOD
Temperature	294.2
Pulse Sequence	zg30
Experiment	1D
Probe	5 mm PABBO BB-1H/ D Z-GRD Z104275/ 0242
Number of Scans	64
Receiver Gain	191.9
Relaxation Delay	1.0000
Pulse Width	14.0000
Spectrometer Frequency	300.13
Spectral Width	6009.6
Lowest Frequency	-1187.1
Nucleus	1H
Acquired Size	32768
Spectral Size	65536

1H NMR (300 MHz, Methanol-d_4) δ 8.47 – 8.37 (m, 2H), 7.87 – 7.78 (m, 1H), 7.43 (d, *J* = 8.2 Hz, 2H), 7.38 – 7.26 (m, 2H), 7.03 (d, *J* = 8.4 Hz, 2H), 2.57 (s, 3H), 1.84 (td, *J* = 8.5, 4.3 Hz, 1H), 0.94 – 0.82 (m, 2H), 0.60 (dt, *J* = 6.7, 4.6 Hz, 2H).

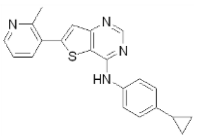

6-(2-Chloropyridin-3-yl)-*N*-(4-(methylthio)phenyl)thieno[3,2-*d*]pyrimidin-4-amine **39**

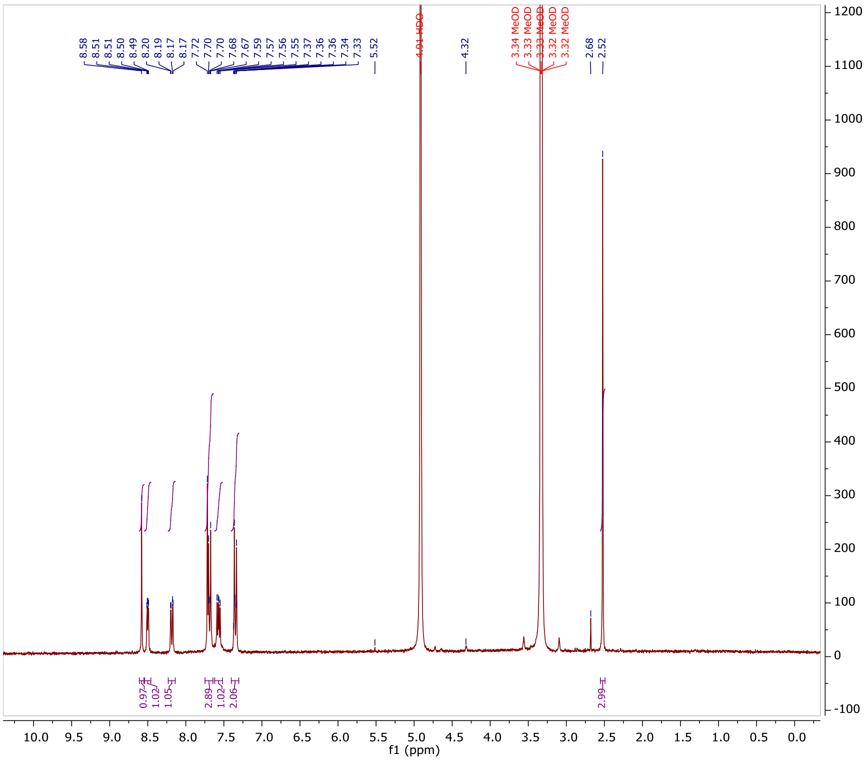

Parameter	Value
Title	tpcr6-001-3_meod.10.fid
Origin	Bruker BioSpin GmbH
Instrument	spect
Solvent	MeOD
Temperature	294.3
Pulse Sequence	zg30
Experiment	1D
Probe	5 mm PABBO BB-1H/ D Z-GRD Z104275/ 0242
Number of Scans	64
Receiver Gain	191.9
Relaxation Delay	1.0000
Pulse Width	14.0000
Spectrometer Frequency	300.13
Spectral Width	6009.6
Lowest Frequency	-1151.4
Nucleus	1H
Acquired Size	32768
Spectral Size	65536

1H NMR (300 MHz, Methanol-d_4) δ 8.58 (s, 1H), 8.50 (dd, *J* = 4.8, 1.9 Hz, 1H), 8.18 (dd, *J* = 7.7, 1.9 Hz, 1H), 7.75 – 7.64 (m, 3H), 7.57 (dd, *J* = 7.7, 4.8 Hz, 1H), 7.40 – 7.30 (m, 2H), 2.52 (s, 3H).

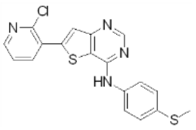

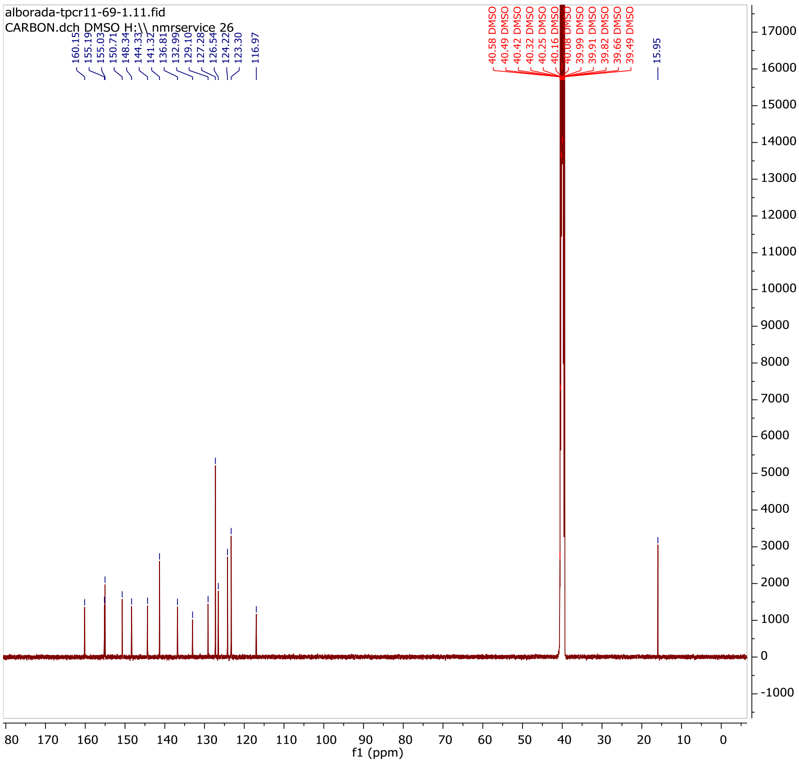

Parameter	Value
Title	alborada-tpcr11-69-1.11.fid
Origin	Bruker BioSpin GmbH
Instrument	aberlour
Solvent	DMSO
Temperature	298.0
Pulse Sequence	zgpg30
Experiment	1D
Probe	Z115434_0002 (CP DCH 500S2 C/ H-D-05 Z ET)
Number of Scans	2048
Receiver Gain	1820.0
Relaxation Delay	2.0000
Pulse Width	9.5000
Spectrometer Frequency	125.75
Spectral Width	34722.2
Lowest Frequency	-3530.0
Nucleus	13C
Acquired Size	104893
Spectral Size	262144

13C NMR (126 MHz, DMSO) δ 160.2, 155.2, 155.0, 150.7, 148.3, 144.3, 141.3, 136.8, 133.0 129.1, 127.3, 126.5, 124.2, 123.3, 117.0, 16.0.

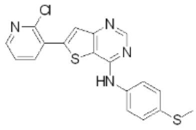

1-(4-((6-(2-Methylpyridin-3-yl)thieno[3,2-*d*]pyrimidin-4-yl)amino)phenyl)pyrrolidin-2-one **42**

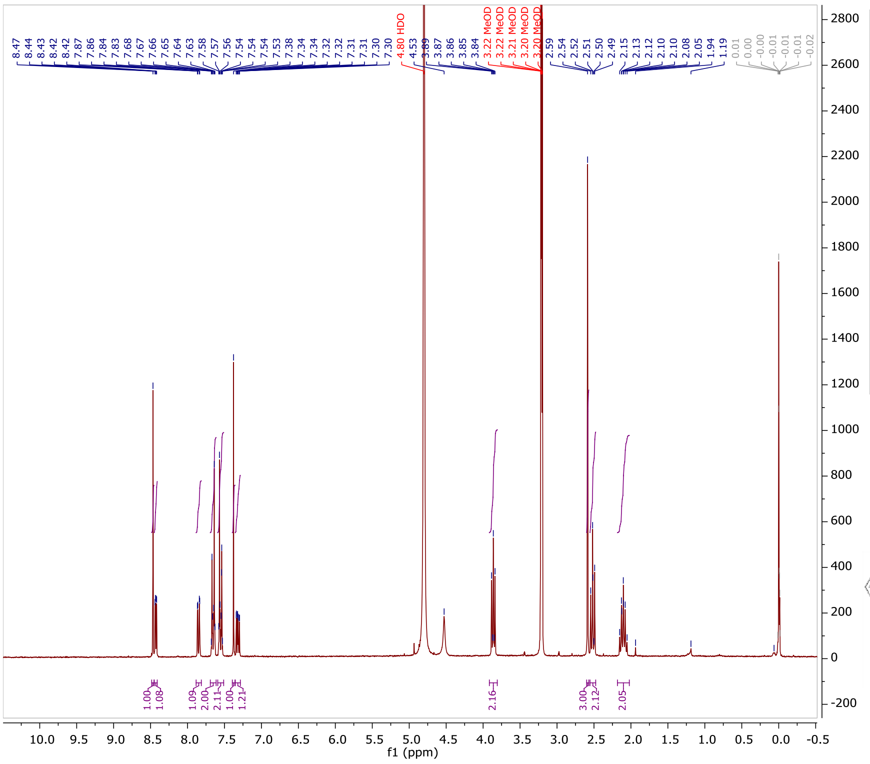

Parameter	Value
Title	tpcr6-010-3.10.fid
Origin	Bruker BioSpin GmbH
Instrument	spect
Solvent	MeOD
Temperature	294.2
Pulse Sequence	zg30
Experiment	1D
Probe	5 mm PABBO BB-1H/ D Z-GRD Z104275/ 0242
Number of Scans	64
Receiver Gain	191.9
Relaxation Delay	1.0000
Pulse Width	14.0000
Spectrometer Frequency	300.13
Spectral Width	6009.6
Lowest Frequency	-1187.3
Nucleus	1H
Acquired Size	32768
Spectral Size	65536

1H NMR (300 MHz, Methanol-d_4) δ 8.47 (s, 1H), 8.43 (dd, *J* = 5.0, 1.7 Hz, 1H), 7.85 (dd, *J* = 7.8, 1.7 Hz, 1H), 7.69 – 7.61 (m, 2H), 7.59 – 7.51 (m, 2H), 7.38 (s, 1H 7.32 (ddd, *J* = 7.8, 5.0, 0.7 Hz, 1H), 3.92 – 3.81 (m, 2H), 2.59 (s, 3H), 2.55 – 2.48 (r 2H), 2.18 – 2.02 (m, 2H).

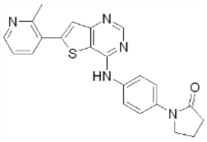

Table S1. Docking scores obtained using 6YM4, 8C8C or 8BQ4 and Glide SP (release 2022-3, Schrodinger, www.schrodinger.com). No constraints were used for docking. For 6YM4 docking the Lys209 conformation was changed to the rotamer that created the largest binding pocket using the MOE protein builder (release 22.02, Chemical Computing Group, www.chemcomp.com). Water molecules that are located in similar positions in 6YM4 and 8C8C were left in the site during docking.

**Manuscript Compound ID**	**Glide Score**			**ADP-Glo pIC$_{50}$**		**SMILES**
	**6YM4**	**8C8C**	**8BQ4**	**PI5P4Kα**	**PI5P4Kγ+**	
	-6.20	-10.52	-7.84	7.65	7.49	CSc1ccc(cc1)Nc1ncnc2cc(sc12)-c1c(noc1C)C
**39**	-8.22	-10.56	-9.98	7.33	8.06	CSc1ccc(cc1)Nc1ncnc2cc(sc12)-c1cccnc1Cl
	-8.01	-10.54	-6.96	7.11	7.77	CSc1ccc(cc1)Nc1ncnc2cc(sc12)-c1cccnc1C
**38**	-8.06	-10.54	-10.23	7.01	7.28	Cc1ncccc1-c1cc2ncnc(c2s1)Nc1ccc(cc1)C1CC1
**42**	-9.25	-7.58	-7.49	6.95	8.08	Cc1ncccc1-c1cc2ncnc(c2s1)Nc1ccc(cc1)N1CCCC1=O
	-7.18	-9.72	-9.05	6.89	7.82	COc1cnccc1-c1cc2c(ncnc2s1)Nc1ccc(cc1)SC
**26**	-7.82	-9.95	-9.20	6.87	*7.71	CSc1ccc(cc1)Nc1ncnc2sc(cc12)-c1ccncc1C
**30**	-6.59	-10.04	-9.45	6.81	7.61	CSc1ccc(cc1)Nc1ncnc2sc(cc12)-c1c(noc1C)C
	-7.98	-10.00	-8.91	6.71	7.17	Clc1ncccc1-c1cc2c(ncnc2s1)Nc1ccc(cc1)C1CC1
**28**	-7.89	-9.86	-9.21	6.64	7.73	CSc1ccc(cc1)Nc1ncnc2sc(cc12)-c1cccnc1Cl
	-8.20	-9.58	-9.66	*6.63	7.53	CSc1ccc(cc1)Nc1ncnc2sc(cc12)-c1cncnc1
	-8.63	-10.40	-10.11	6.62	7.07	COc1cnccc1-c1cc2c(ncnc2[nH]1)Nc1ccc(cc1)C1CC1
**24**	-7.80	-10.03	-8.35	6.62	6.96	Cc1ncccc1-c1cc2c(ncnc2s1)Nc1ccc(cc1)C1CC1
**29**	-8.05	-9.64	-8.46	6.59	7.37	CSc1ccc(cc1)Nc1ncnc2sc(cc12)-c1cnoc1
	-7.89	-9.76	-8.24	6.58	6.37	Cc1ccc(cc1)Nc1ncnc2sc(cc12)-c1cccnc1Cl
	-6.09	-9.63	-9.21	6.57	7.20	COc1cnccc1-c1cc2c(ncnc2s1)Nc1ccc(cc1)C1CC1
	-5.74	-9.53	-7.90	6.56	6.20	COc1cnccc1-c1cc2c(ncnc2s1)Nc1ccc(c(c1)C)C
**25**	-7.71	-9.90	-8.41	*6.55	7.33	CSc1ccc(cc1)Nc1ncnc2sc(cc12)-c1cccnc1C
	-7.55	-9.60	-9.02	6.49	6.82	CCc1ccc(cc1)Nc1ncnc2sc(cc12)-c1cccnc1Cl
**18**	-8.31	-9.85	-8.19	6.48	5.87	Cc1ccc(cc1C)Nc1ncnc2sc(cc12)-c1cccnc1C
**37**	-7.67	-8.98	-10.37	6.45	7.93	CS(=O)(=O)c1ccc(cc1)Nc1ncnc2cc(sc12)-c1cccnc1Cl
	-8.07	-9.84	-8.45	6.31	5.07	Cc1ccc(cc1C)Nc1ncnc2sc(cc12)-c1cccnc1Cl
	-6.04	-10.38	-7.26	6.31	7.27	COc1cnccc1-c1cc2ncnc(c2s1)Nc1ccc(cc1)SC
**23**	-7.49	-9.91	-8.32	6.22	6.41	CCc1ccc(cc1)Nc1ncnc2sc(cc12)-c1cccnc1C
**22**	-7.29	-9.80	-8.41	6.22	5.81	Cc1ccc(cc1)Nc1ncnc2sc(cc12)-c1cccnc1C
**10**	-7.32	-7.08	-9.61	6.15	7.89	Cc1cnccc1-c1cc2c(ncnc2s1)Nc1ccc(cc1)S(=O)(=O)C
**15**	-8.17	-7.51	-10.00	6.15	7.89	CS(=O)(=O)c1ccc(cc1)Nc1ncnc2sc(cc12)-c1cccnc1Cl
**27**	-7.15	-10.16	-8.53	6.15	6.18	COc1ccc(cc1)Nc1ncnc2sc(cc12)-c1ccncc1C
**14**	-8.54	-7.59	-10.28	6.00	*7.91	Cc1ccncc1-c1cc2c(ncnc2s1)Nc1ccc(cc1)S(=O)(=O)C
	-7.06	-7.11	-9.68	*5.96	7.07	CS(=O)(=O)c1ccc(cc1)Nc1ncnc2sc(cc12)-c1ccc(cc1F)F
	-8.32	-8.88	-7.97	*5.95		CN(C(=O)C)c1ccc(cc1)Nc1ncnc2sc(cc12)C
**17**	-8.31	-7.38	-9.57	*5.95	7.68	Cc1ncccc1-c1cc2c(ncnc2s1)Nc1ccc(cc1)S(=O)(=O)C
	-6.74	-6.80	-8.35	*5.93	6.88	Cc1cc2c(ncnc2s1)Nc1ccc(cc1)N1CC(C)(C)CC1=O
**20**	-7.97	-9.74	-8.67	5.88	5.72	COc1ccc(cc1)Nc1ncnc2sc(cc12)-c1cccnc1C
	-7.12	-9.81	-8.54	5.88	7.20	COc1cnccc1-c1cc2c(ncnc2s1)Nc1ccc(cc1)Cl

**7**	-8.26	-7.33	-9.71	5.88	7.81	CS(=O)(=O)c1ccc(cc1)Nc1ncnc2sc(cc12)-c1cn[nH]c1
**6**	-8.03	-7.36	-9.45	5.85	7.65	CS(=O)(=O)c1ccc(cc1)Nc1ncnc2sc(cc12)-c1ccncc1
	-7.79	-9.51	-8.36	5.84	5.63	Cc1cccc(c1)Nc1ncnc2sc(cc12)-c1cccnc1Cl
**16**	-8.42	-7.18	-9.80	*5.83	7.50	CS(=O)(=O)c1ccc(cc1)Nc1ncnc2sc(cc12)-c1cccnc1F
	-8.64	-7.00	-9.77	5.66	7.44	CS(=O)(=O)c1ccc(cc1)Nc1ncnc2sc(cc12)-c1cnoc1
	-8.35	-7.59	-9.75	5.64	7.54	COc1ncccc1-c1cc2c(ncnc2s1)Nc1ccc(cc1)S(=O)(=O)C
	-7.79	-8.73	-9.36	5.63	6.75	CSc1ccc(cc1)Nc1ncnc2sc(cc12)C
**11**	-4.52	-7.64	-9.68	*5.58	*7.49	COc1cnccc1-c1cc2c(ncnc2s1)Nc1ccc(cc1)S(=O)(=O)C
	-7.29	-7.33	-9.55	5.56		CC(C)c1ccc(cc1)Nc1ncnc2sc(c(c12)C)C(=O)O
	-7.52	-10.02	-7.96	*5.53	6.41	Cc1cc(ccc1Cl)Nc1ncnc2sc(cc12)-c1cccnc1Cl
	-7.76	-10.16	-8.52	5.53	5.08	Cc1ccc(c(c1)C)Nc1ncnc2sc(cc12)-c1cccnc1Cl
**19**	-8.01	-10.13	-9.46	5.48	5.36	Cc1ccc(c(c1)C)Nc1ncnc2sc(cc12)-c1cccnc1C
**41**	-8.45	-10.56	-9.30	5.48	6.25	Cc1ncccc1-c1cc2ncnc(c2s1)Nc1ccc2ncoc2c1
	-5.31	-7.44	-9.59	5.47	7.28	COc1cnccc1-<br>c1cc2c(ncnc2s1)Nc1ccc(cc1)S(=O)(=O)C1CCCC1
**21**	-8.02	-9.06	-8.19	5.45	5.28	Cc1cccc(c1)Nc1ncnc2sc(cc12)-c1cccnc1C
	-8.99	-8.10	-10.04	5.45	7.01	CS(=O)(=O)c1ccc(cc1)Nc1ncnc2sc(cc12)C#N
	-7.75	-8.63	-9.79	*5.43	*6.75	CS(=O)(=O)c1ccc(cc1)Nc1ncnc2sc(cc12)C(=O)O
	-6.09	-7.50	-9.20	5.36	7.90	CC(=O)NCc1cc2c(ncnc2s1)Nc1ccc(cc1)S(=O)(=O)C1CCCC1
**9**	-8.55	-7.15	-9.88	*5.34	7.46	CS(=O)(=O)c1ccc(cc1)Nc1ncnc2sc(cc12)-c1cccnc1
	-8.64	-8.57	-8.35	*5.34	6.79	Cc1cc2c(ncnc2s1)Nc1ccc(cc1)N1CCCC1=O
	-4.84	-7.93	-10.14	5.33	7.31	O=S(=O)(C1CCCC1)c1ccc(cc1)Nc1ncnc2sc(cc12)C#N
	-7.53	-10.22	-6.71	5.30	5.13	COc1cnccc1-c1cc2c(ncnc2s1)Nc1ccc(cc1C)C
**5**	-5.92	-7.35	-9.36	5.29	7.39	CS(=O)(=O)c1ccc(cc1)Nc1ncnc2sc(cc12)-c1ccccc1
	-7.84	-9.93	-8.40	5.28	5.10	Cc1cc(ccc1Nc1ncnc2sc(cc12)-c1cccnc1Cl)Cl
	-7.67	-6.55	-9.70	5.28	7.40	FC(F)(F)C1(N=N1)c1ccc(cc1)C(=O)NCc1cc2c(ncnc2s1)<br>Nc1ccc(cc1)S(=O)(=O)C1CCCC1
	-8.78	-7.35	-8.26	5.27	6.70	Cc1cc2c(ncnc2s1)Nc1ccc(c(c1)C)N1CCCC1=O
**8**	-5.71	-7.16	-9.85	5.20	7.44	Cn1cc(cn1)-c1cc2c(ncnc2s1)Nc1ccc(cc1)S(=O)(=O)C
	-6.70	-7.01	-10.16	5.16	6.25	CN(C)C(=O)c1cc2c(ncnc2s1)Nc1ccc(cc1)S(=O)(=O)C
	-7.48	-8.37	-8.91	5.16		Cc1cc2c(ncnc2s1)Nc1ccc(cc1)Br
	-6.25	-7.19	-8.36	5.14	6.67	COc1cc(ccc1N1CCCC1=O)Nc1ncnc2sc(cc12)C
	-7.72	-7.63	-9.77	5.11	7.12	Cc1cc2c(ncnc2s1)Nc1ccc(cc1)S(=O)(=O)C1CC1
**13**	-6.41	-7.11	-9.51	5.08	7.19	CS(=O)(=O)c1ccc(cc1)Nc1ncnc2sc(cc12)-c1ccnc(c1)Cl
	-8.18	-7.90	-10.08	5.06	6.78	CS(=O)(=O)c1ccc(cc1)Nc1ncnc2sc(cc12)C(F)F
	-7.00	-6.83	-9.76	5.05	6.12	COC(=O)c1cc2c(ncnc2s1)Nc1ccc(cc1)S(=O)(=O)C
	-8.34	-8.08	-9.92	5.04	7.06	CS(=O)(=O)c1ccc(cc1)Nc1ncnc2sc(cc12)C(F)(F)F
	-8.39	-7.62	-8.35	5.01	6.54	CCc1cc2c(ncnc2s1)Nc1ccc(c(c1)C)N1CCCC1=O
	-7.60	-8.56	-9.89	5.01	7.88	Cc1cc2c(ncnc2s1)Nc1ccc(cc1)S(=O)(=O)C1CCCC1
	-7.86	-7.14	-9.21	5.01	7.28	CS(=O)(=O)c1ccc(cc1)Nc1ncnc2sc(cc12)-c1ccc(cc1)F

ADMET experimental methods

MDR1-MDCK Permeability (bi-directional): was performed by Cyprotex Discovery. Briefly, compounds were administered at 10 µM (1% DMSO final) to the apical or basolateral side of a confluent monolayer of MDR1-MDCK cells, then incubated at 37 °C for 60 minutes before appearance on the opposite side of the monolayer was determined LC-MS/MS. The efflux ratio (ER) is calculated from the ratio of B-A to A-B permeabilities.

Turbidimetric aqueous solubility: analysis was performed by Cyprotex Discovery. Compound in DMSO at 10 mM was serially diluted to solutions of 0.1, 0.3, 1 and 3 mM in DMSO, then further diluted 1 in 100 in buffer (0.01 M PBS, pH 7.4, 1% DMSO final). 7 replicate wells of each dilution were equilibrated for 2 hours at 37 °C before absorbance is measured at 620 nm. The solubility is estimated from the concentration of test compound that produces an increase in absorbance above vehicle control (1% DMSO in buffer).

Mouse Microsomal stability: analysis was performed by Cyprotex Discovery. Briefly, test compounds in DMSO were incubated at a concentration of 1 µM (0.25% DMSO final) with mouse hepatic microsomes (0.5 mg protein/mL) in the presence of NADPH (1 mM) at 37 °C. Aliquots were taken at time intervals (0, 5, 15, 30 and 45 min) and stopped by transferring into acetonitrile, then analysed using generic LC-MS/MS conditions for compound remaining, allowing the determination of the half-life for the compound.
